# Supplementary material for: ERH regulates type II interferon immune signaling through post-transcriptional regulation of JAK2 mRNA
Source: Nucleic Acids Res. 2025 Jun 30;53(12):gkaf545. doi: 10.1093/nar/gkaf545 (PMC12207402; doi:10.1093/nar/gkaf545)

Table of Contents

Table of contents........ **0**

Souce_data_Fig_2B **1-10**

Souce_data_Fig_2E **11-32**

Souce_data_Fig_2G **33-45**

Souce_data_Fig_3B **46-53**

Souce_data_Fig_3E **54-66**

Souce_data_Fig_4D **67-79**

Souce_data_Fig_S2E **80-97**

Souce_data_Fig_S3D **98-102**

Souce_data_Fig_S4A **103-104**

RKO, ERH (20% Gel)

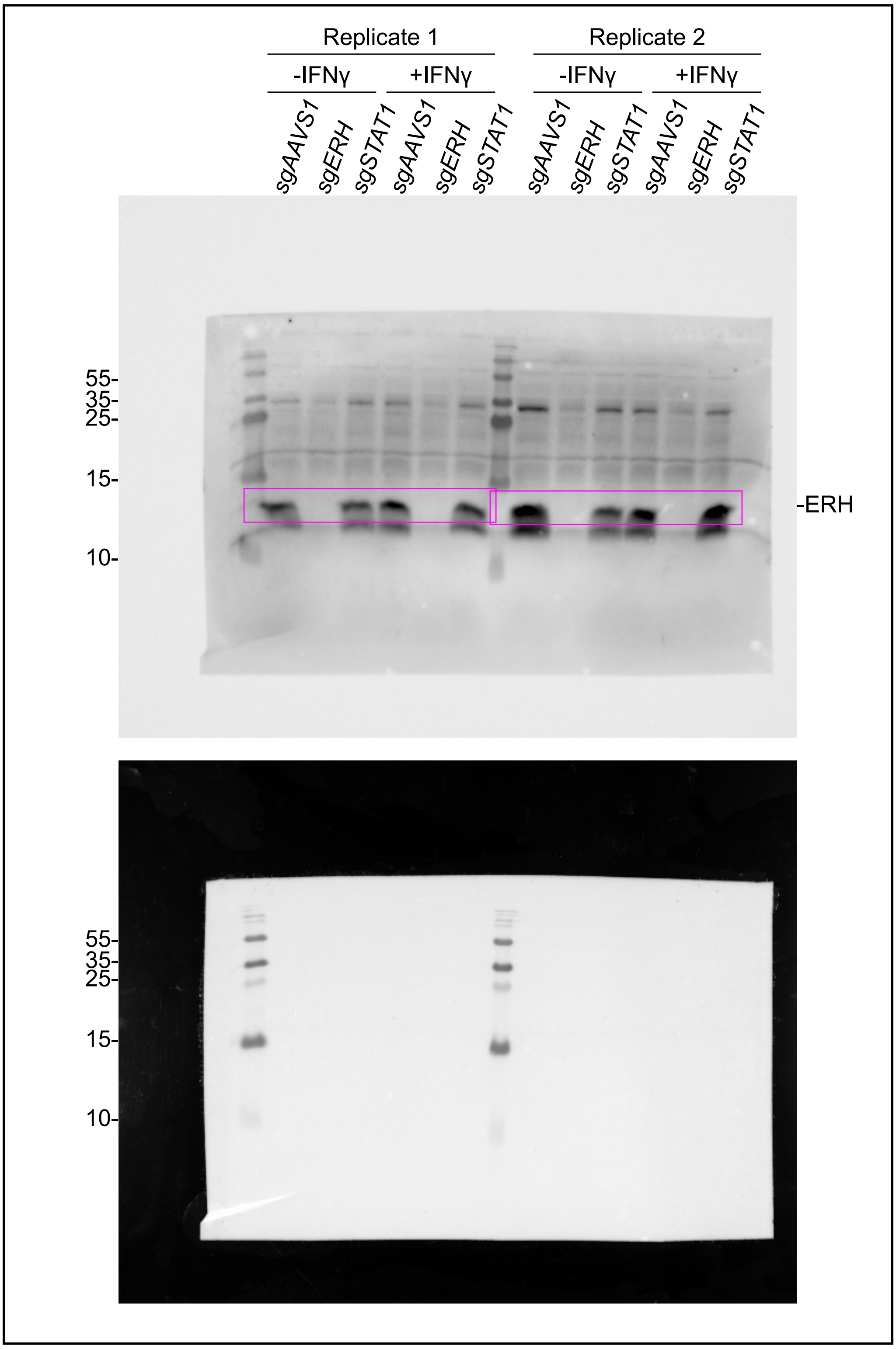

RKO, β-Actin (20% Gel)

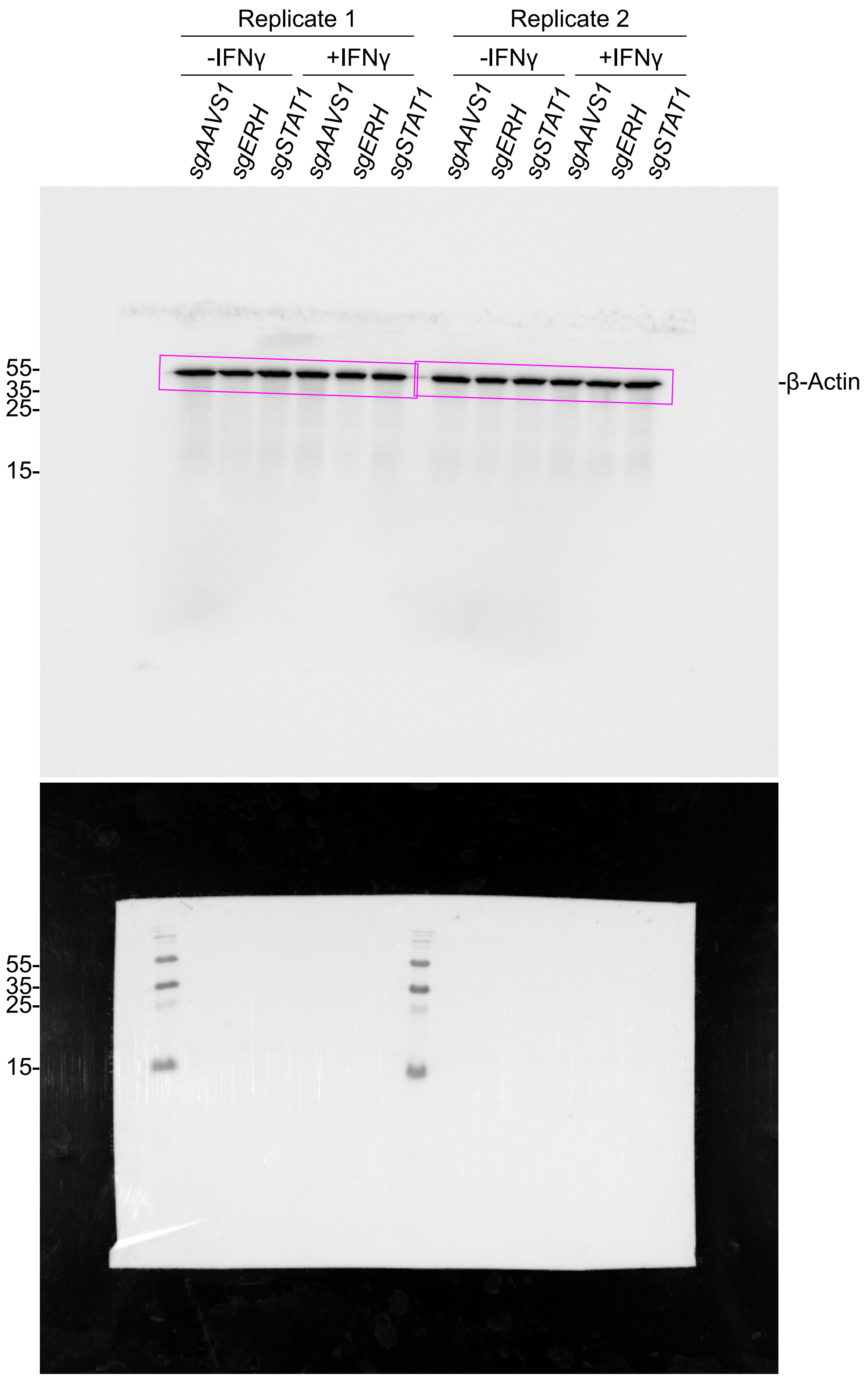

RKO, β-Actin (10% Gel)

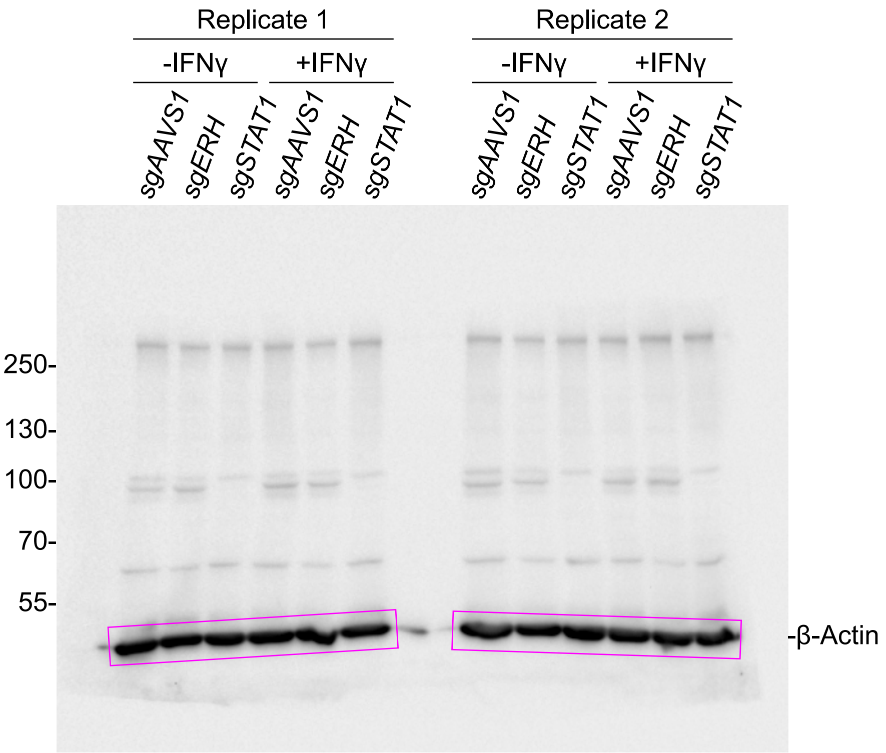

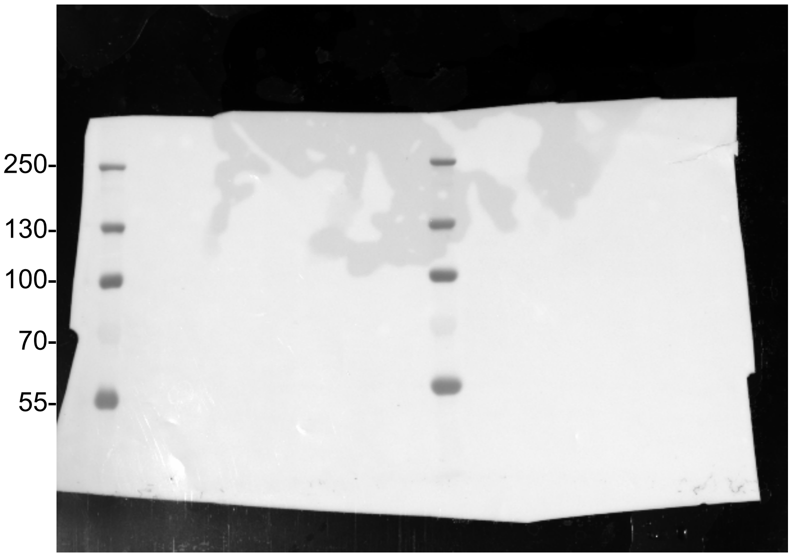

RKO, JAK1 (10% Gel)

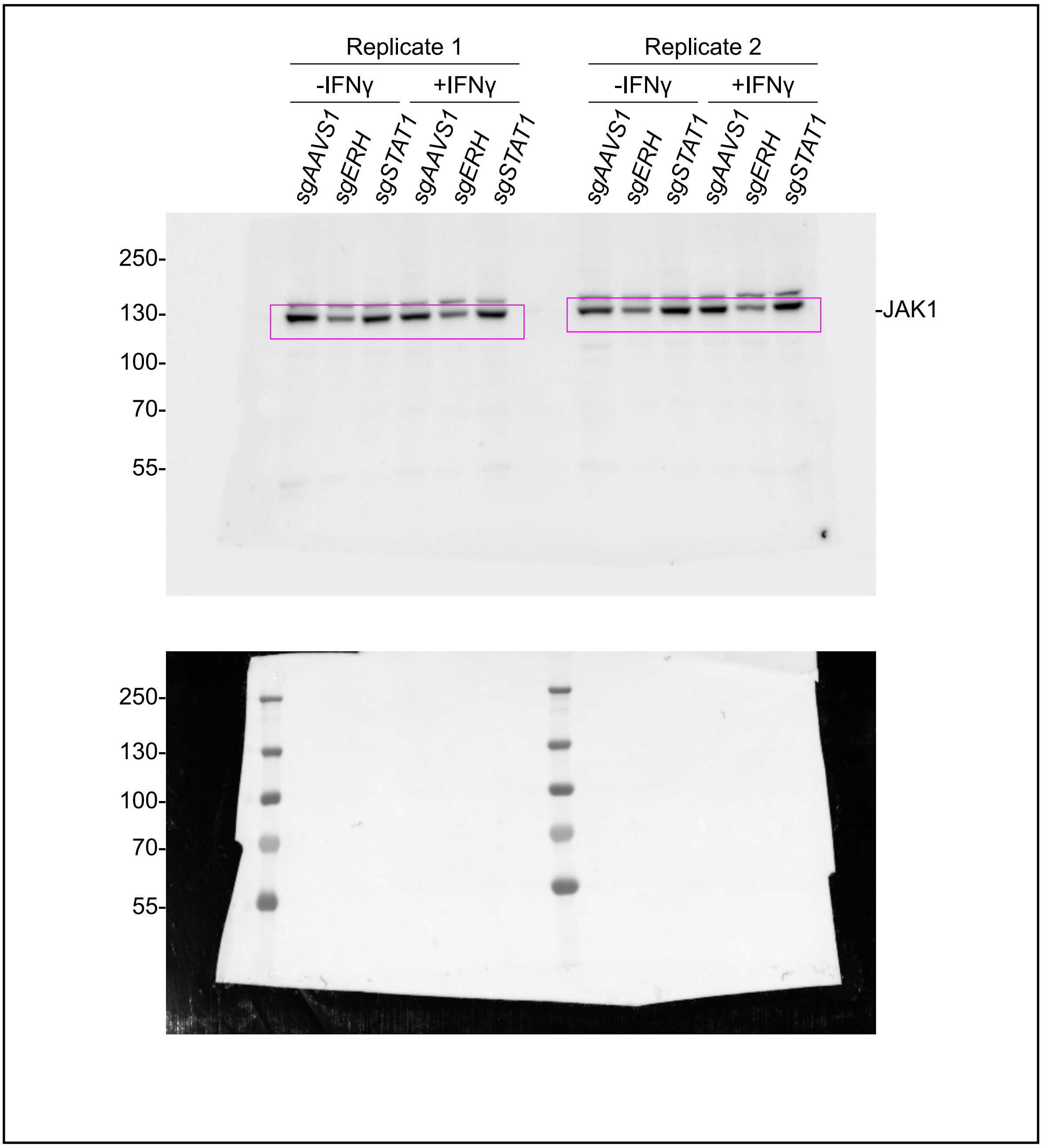

RKO, STAT1 (10% Gel)

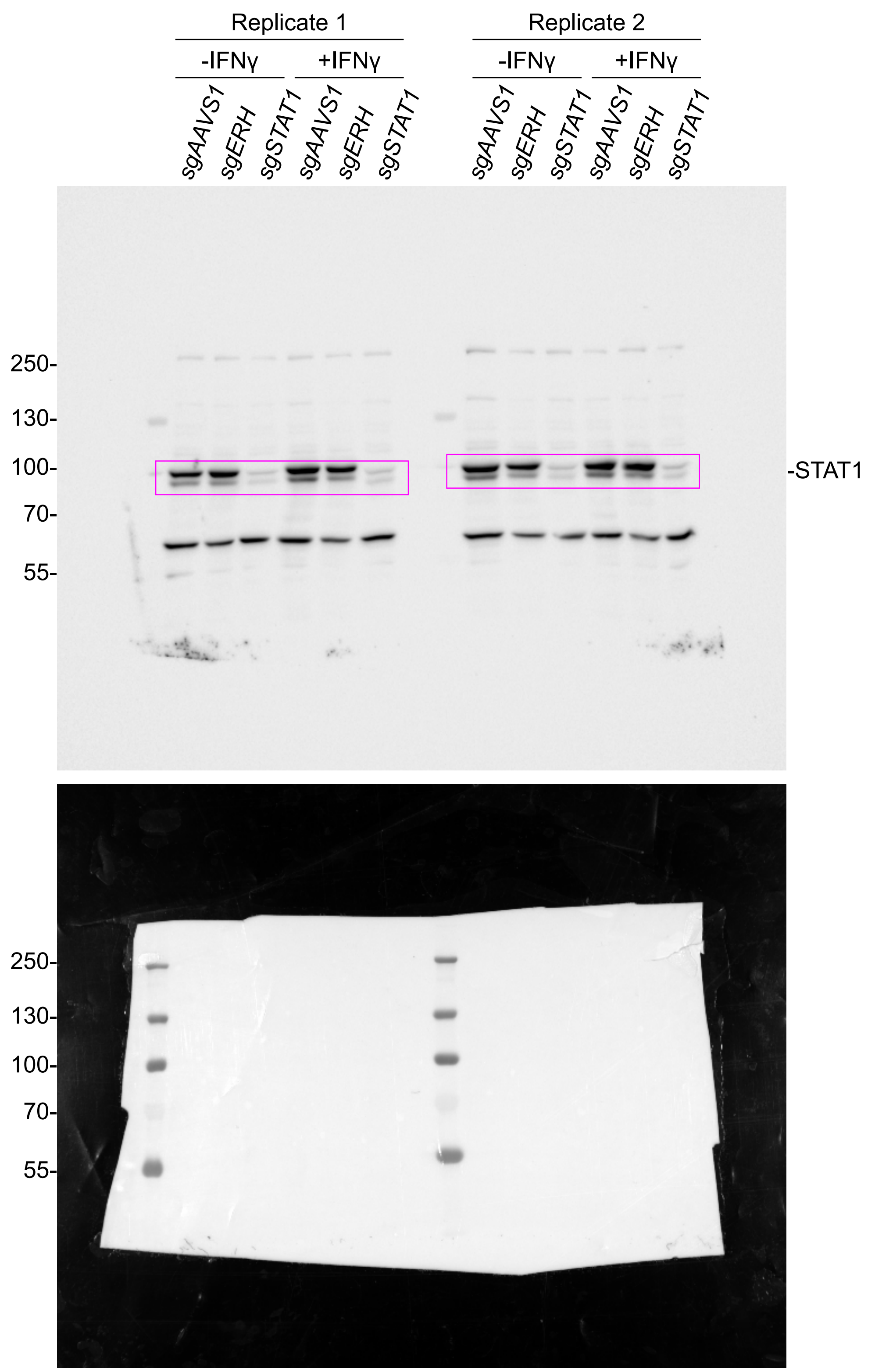

RKO, β-Actin (8% Gel)

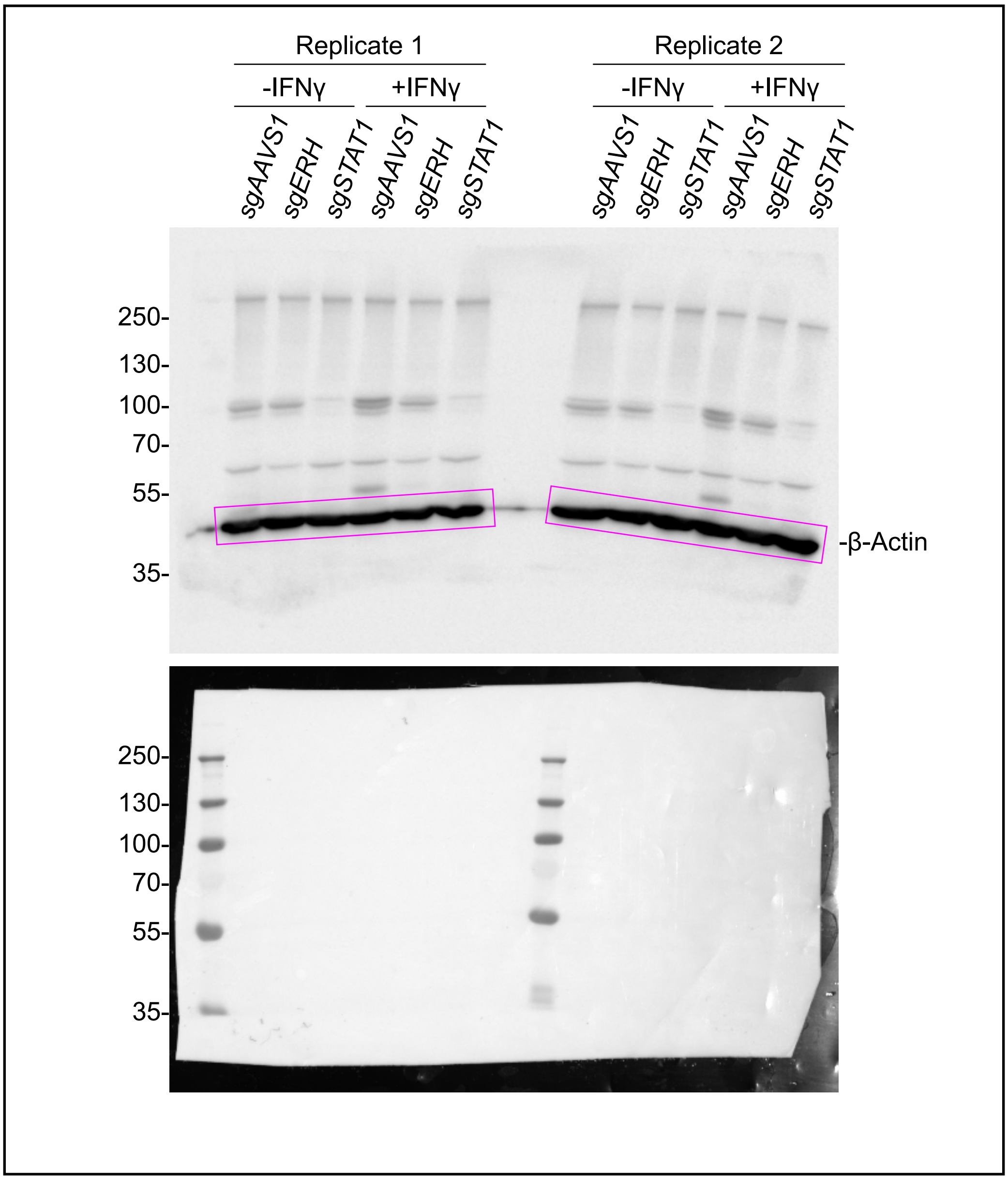

Souce_data_Fig_2B

RKO, IRF1 (8% Gel)

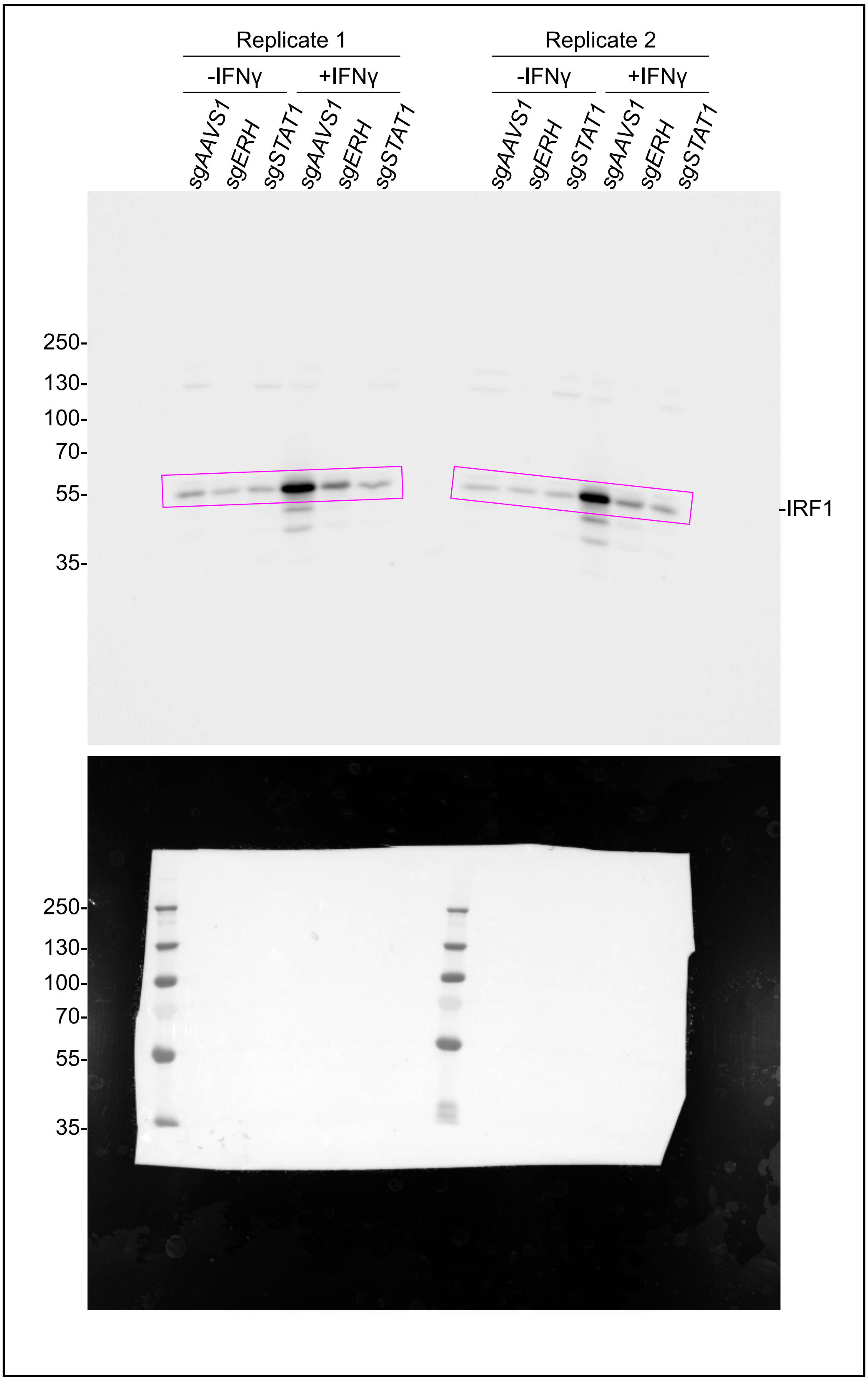

RKO, JAK2 (8% Gel)

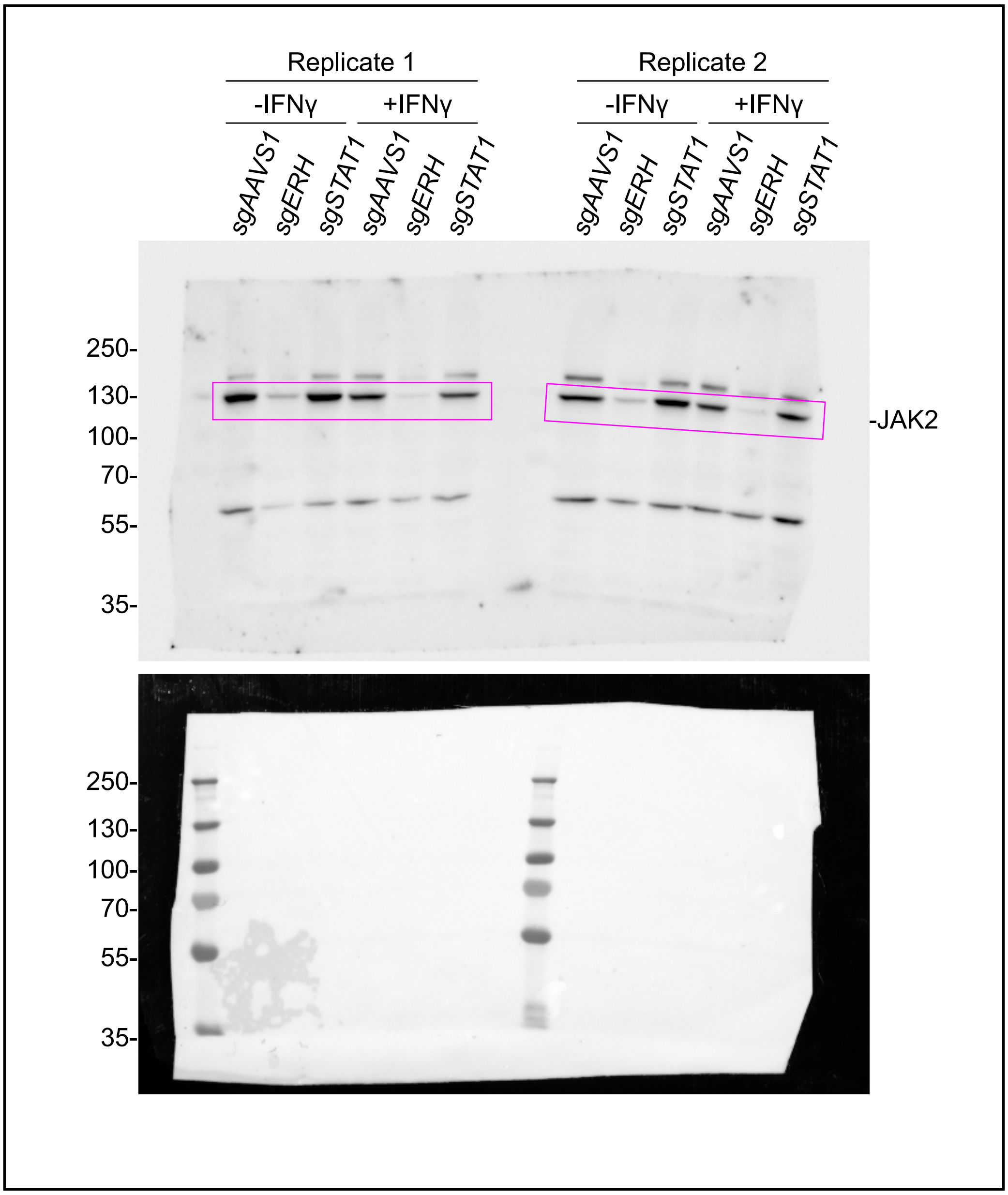

RKO, p-STAT1 (8% Gel)

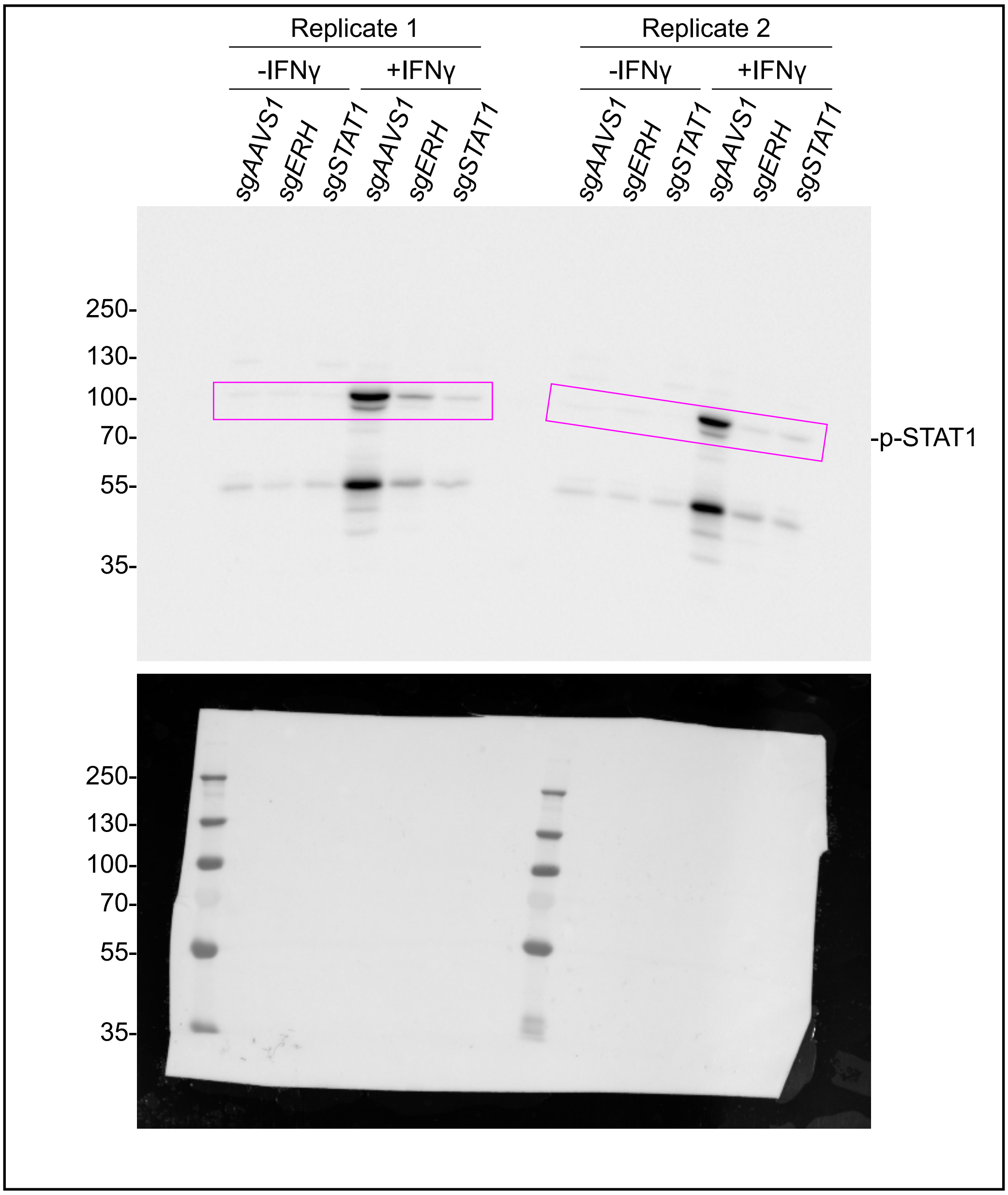

Souce_data_Fig_2B

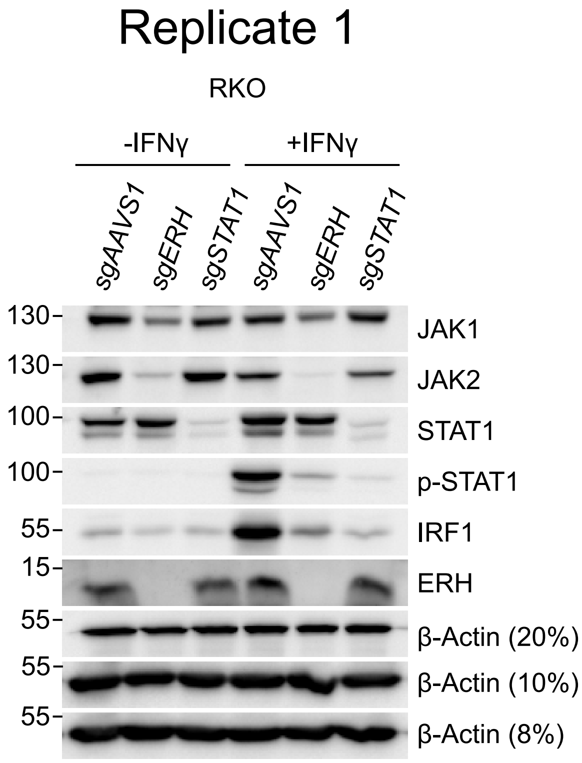

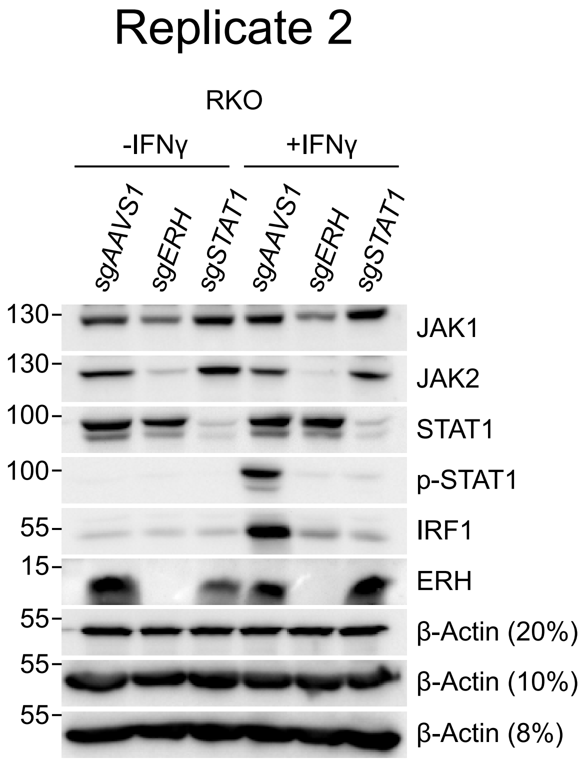

RKO, JAK1 (8% Gel 1), Replicate 1

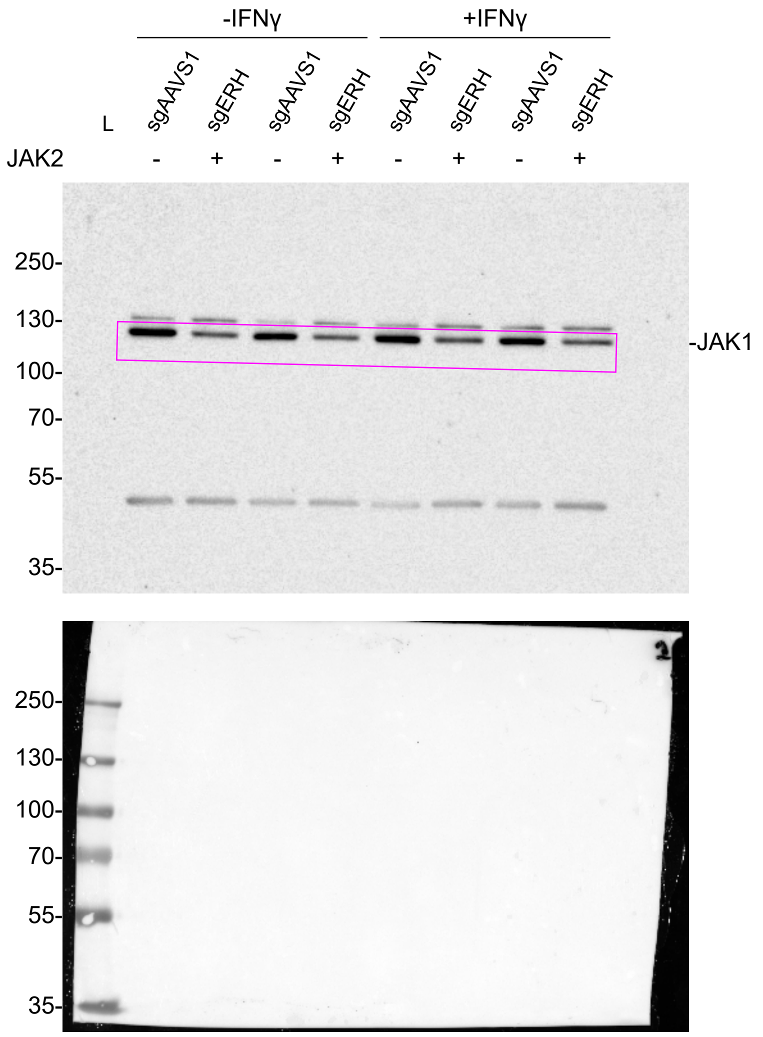

RKO, JAK1 (8% Gel 2), Replicate 2

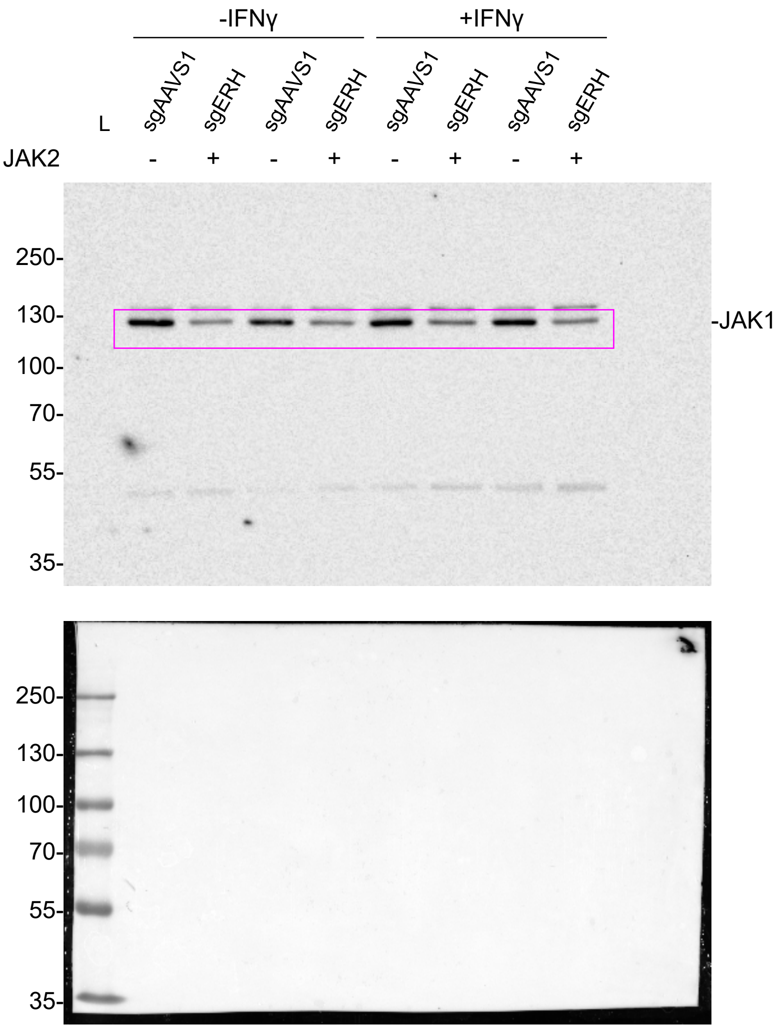

RKO, JAK1 (8% Gel 3), Replicate 3

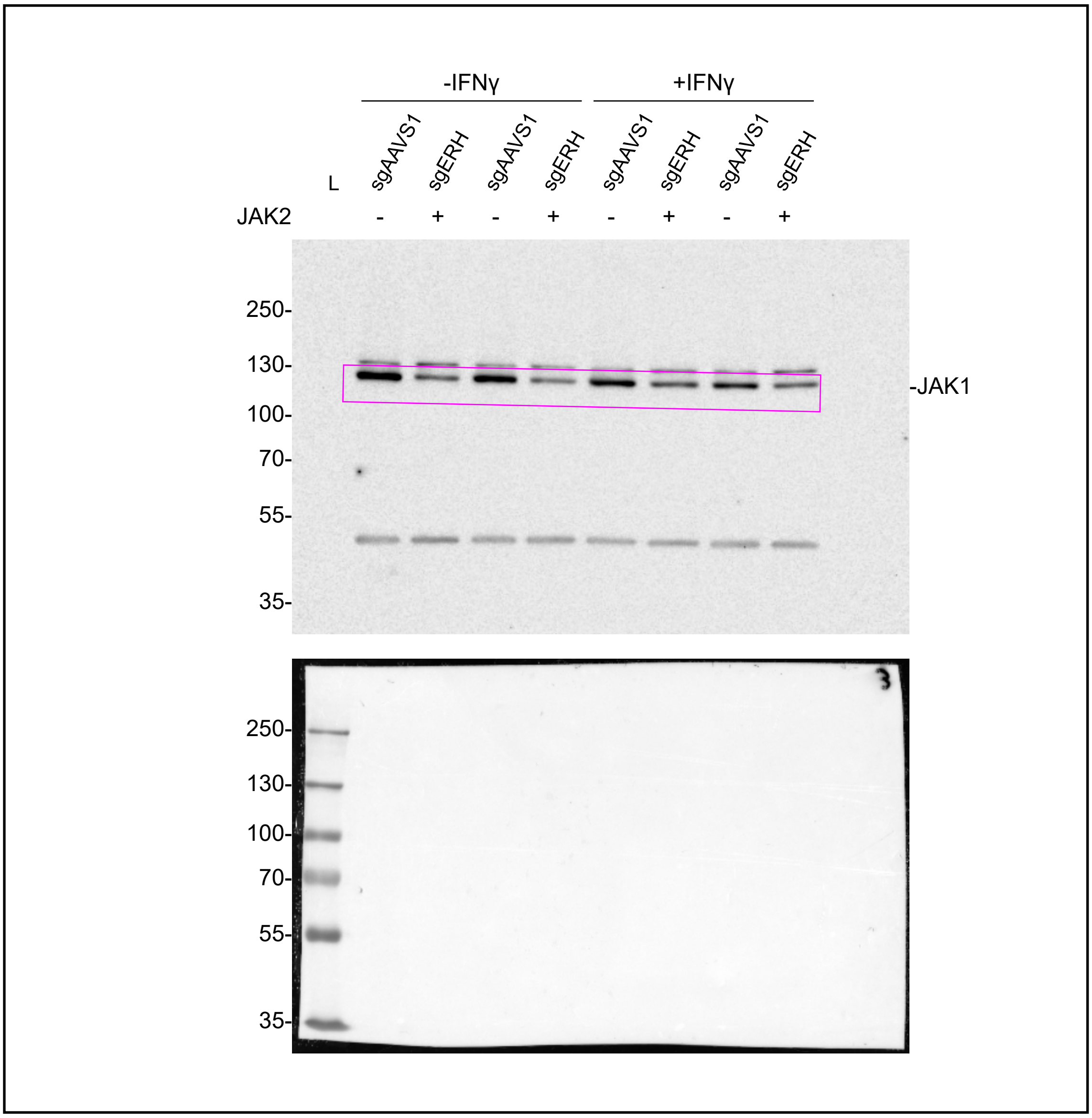

RKO, JAK2 (8% Gel 4), Replicate 1

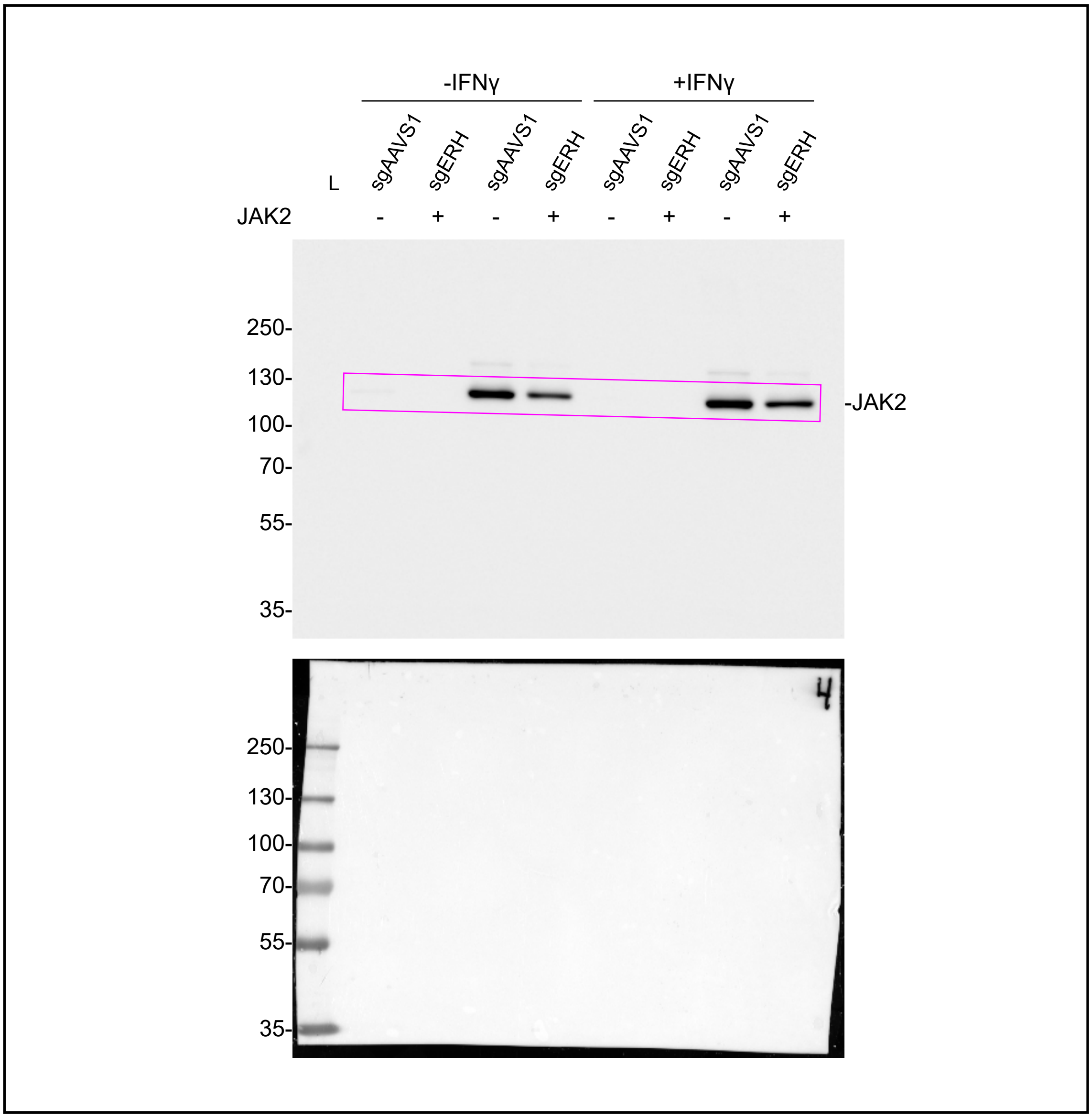

RKO, JAK2 (8% Gel 5), Replicate 2

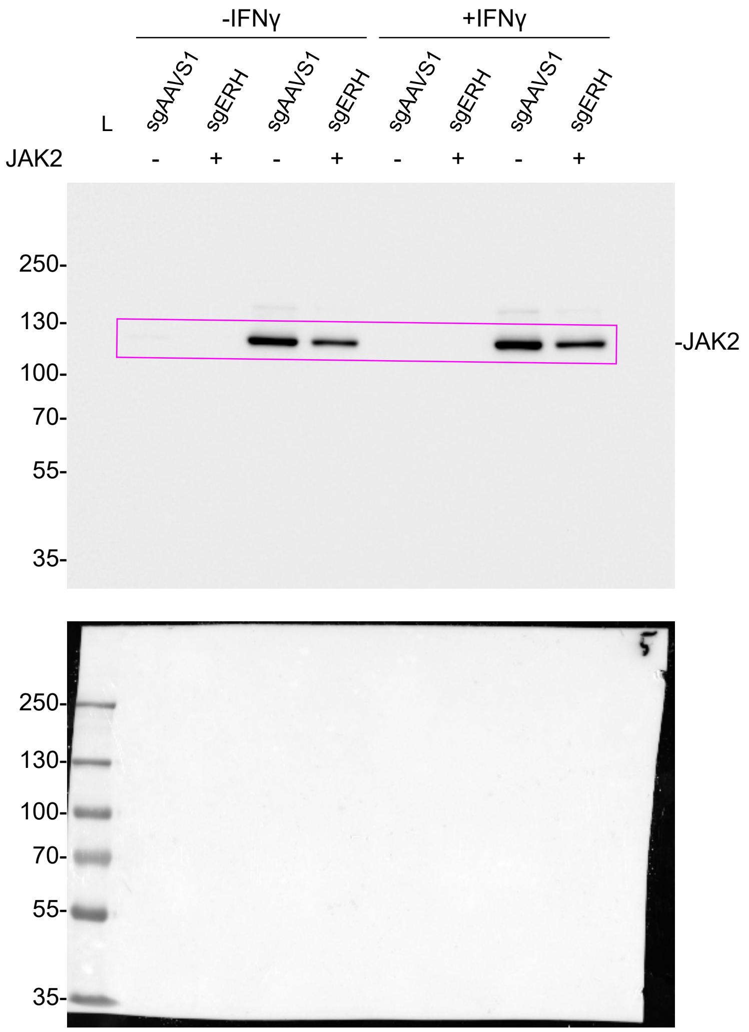

RKO, JAK2 (8% Gel 6), Replicate 3

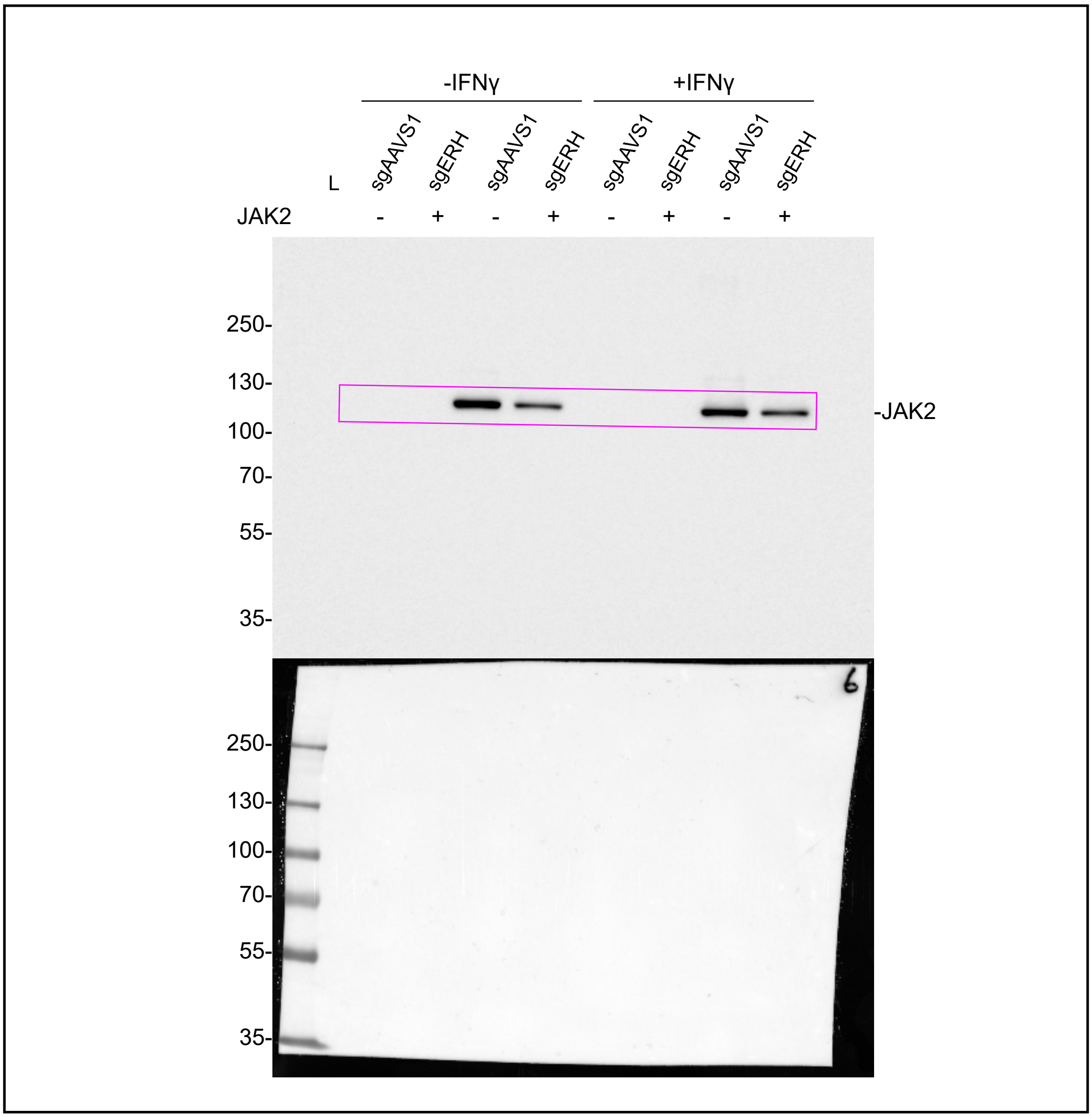

RKO, STAT1 (8% Gel 1), Replicate 1

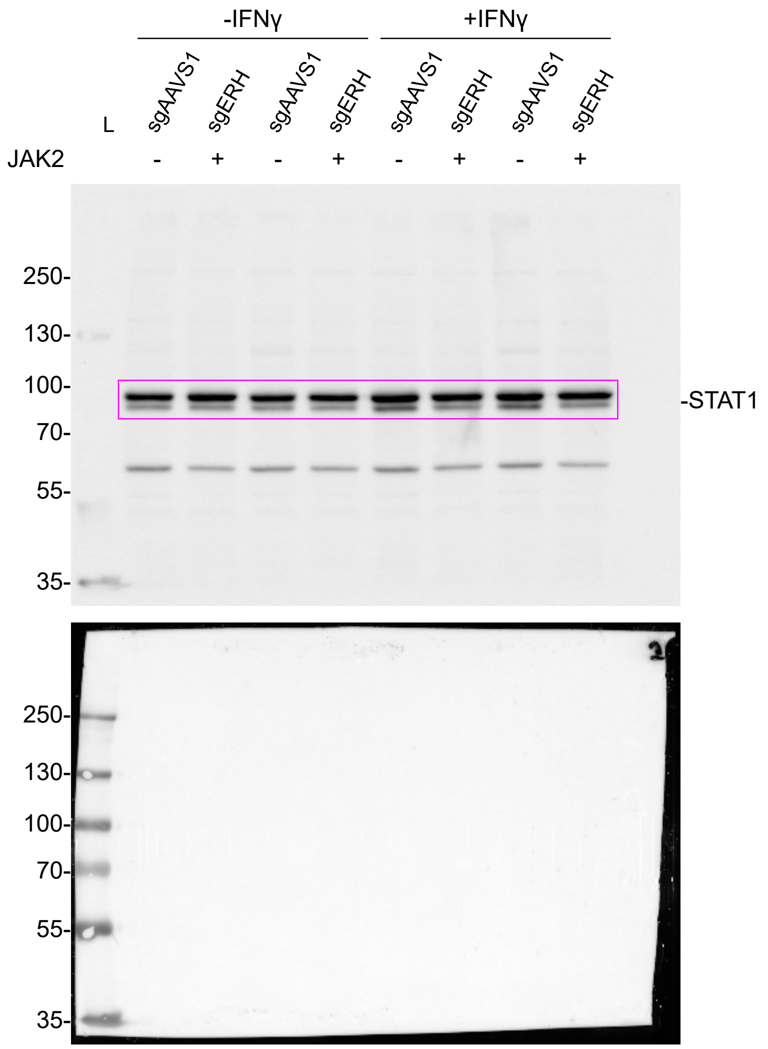

RKO, STAT1 (8% Gel 2), Replicate 2

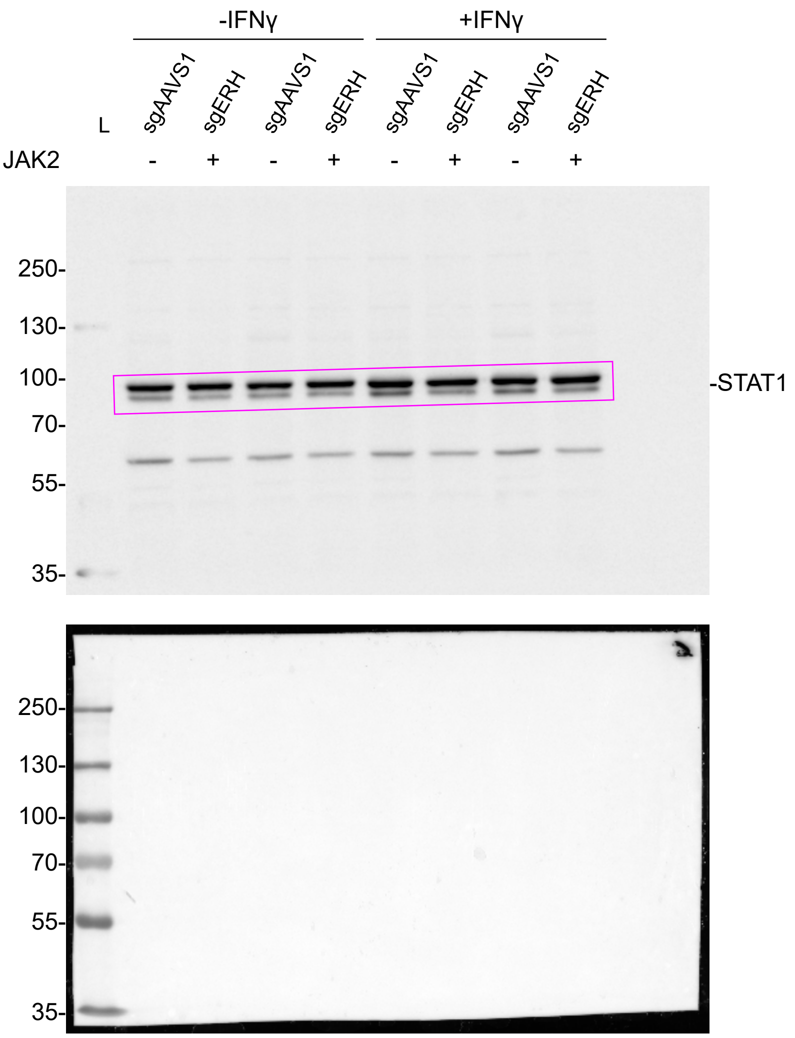

RKO, STAT1 (8% Gel 3), Replicate 3

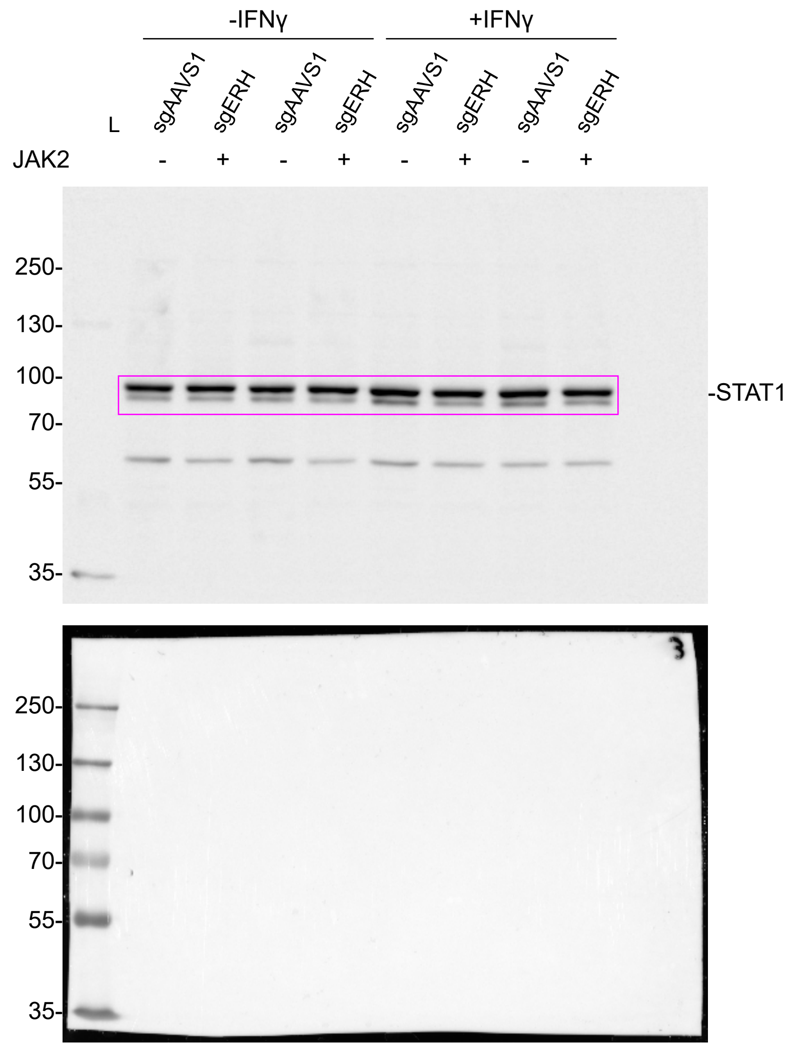

RKO, p-STAT1 (8% Gel 4), Replicate 1

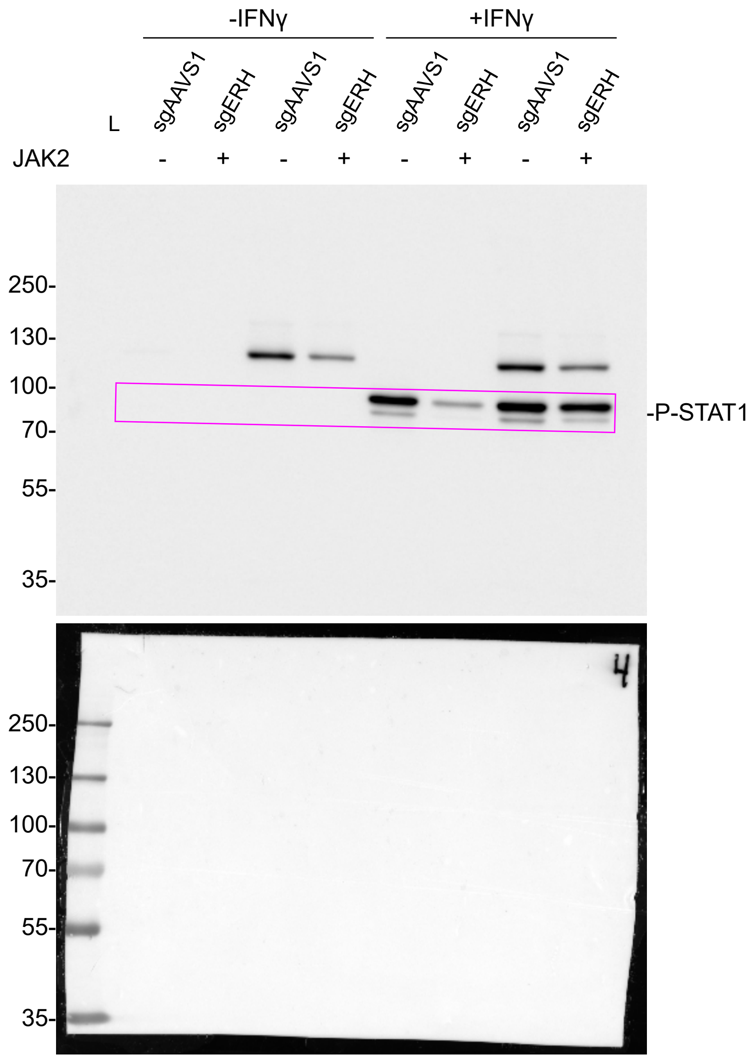

RKO, p-STAT1 (8% Gel 5), Replicate 2

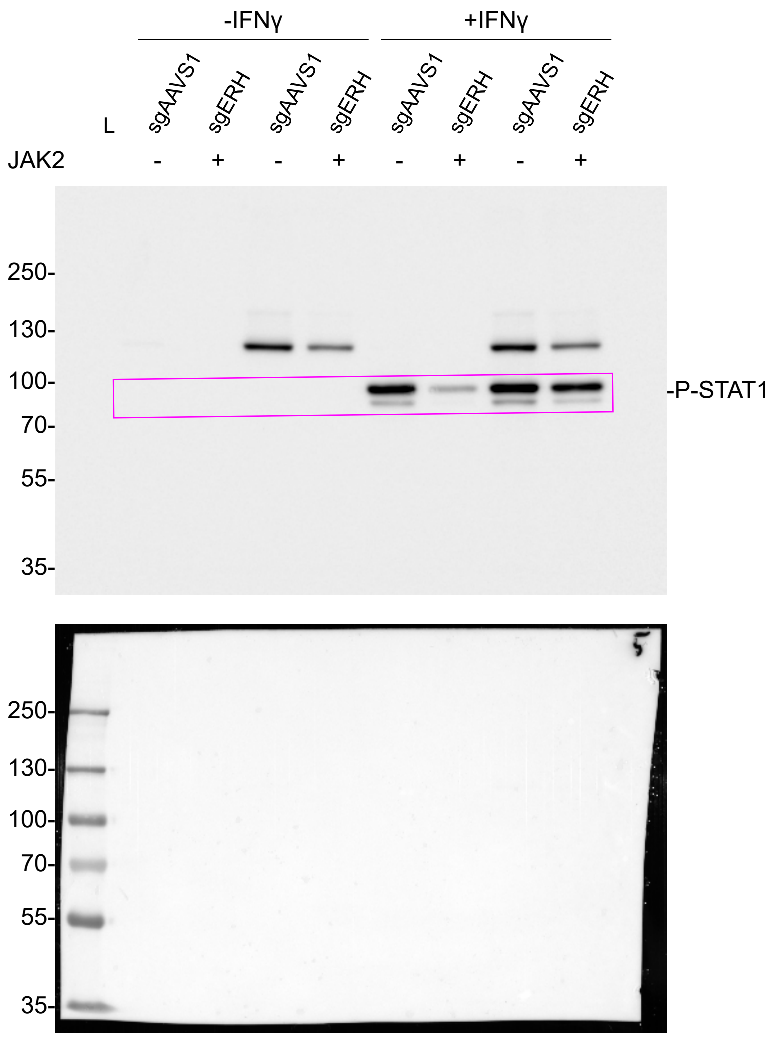

RKO, p-STAT1 (8% Gel 6), Replicate 3

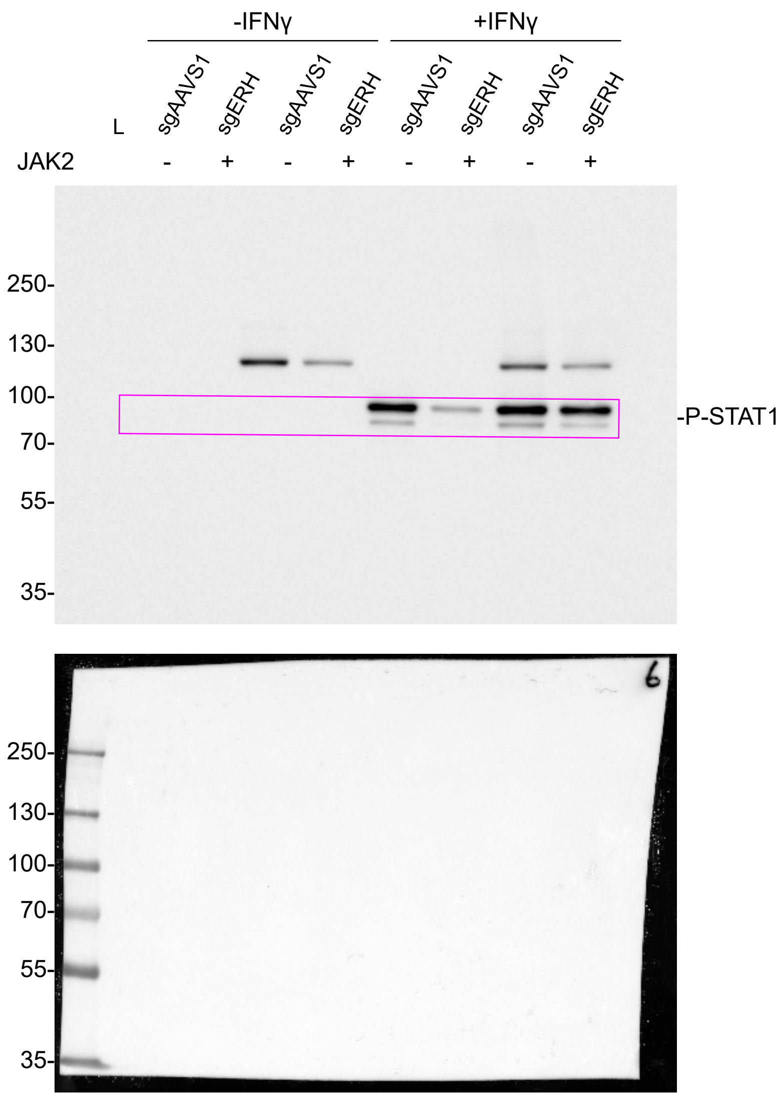

RKO, IRF1 (8% Gel 4), Replicate 1

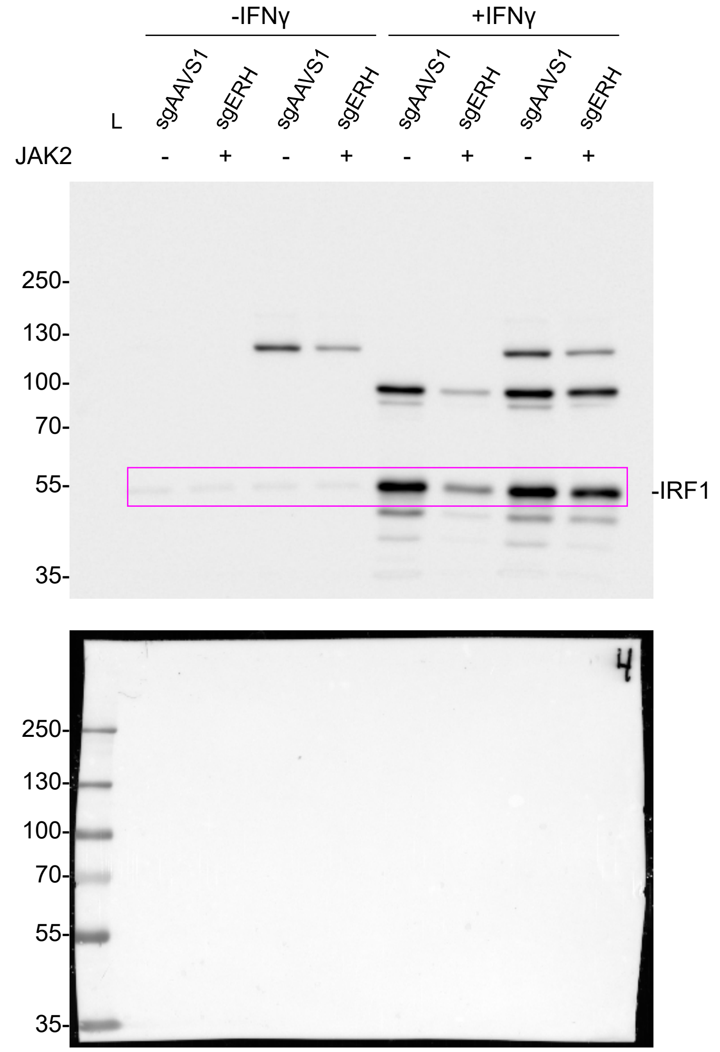

RKO, IRF1 (8% Gel 5), Replicate 2

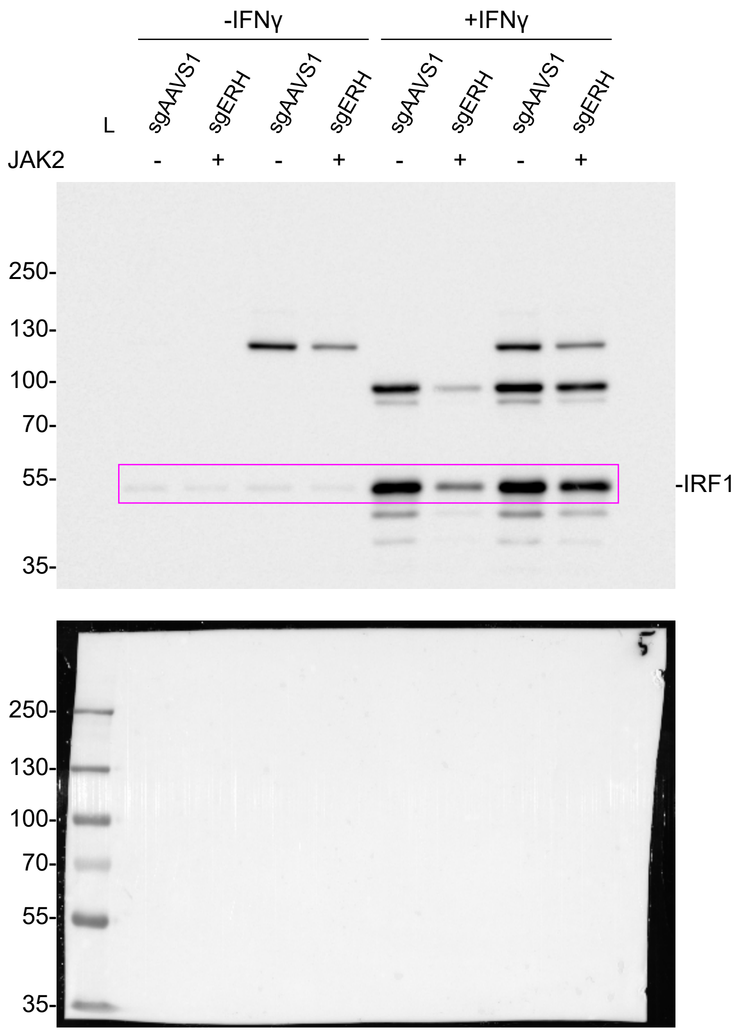

RKO, IRF1 (8% Gel 6), Replicate 3

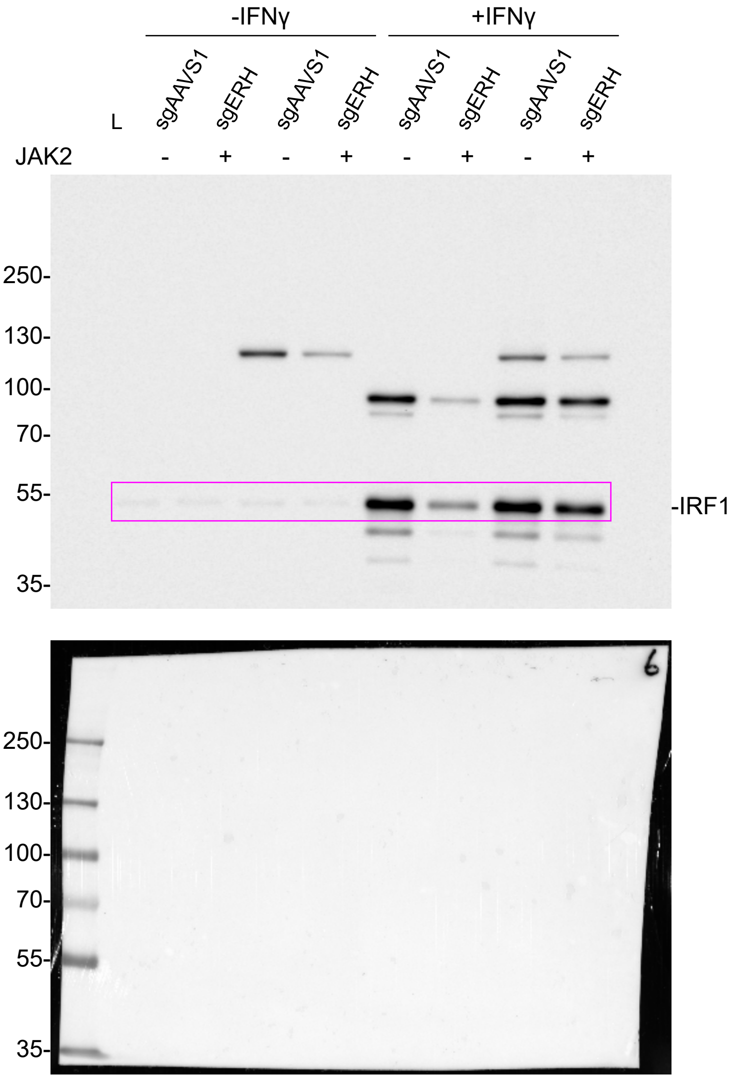

RKO, α-Tubulin (8% Gel 1), Replicate 1

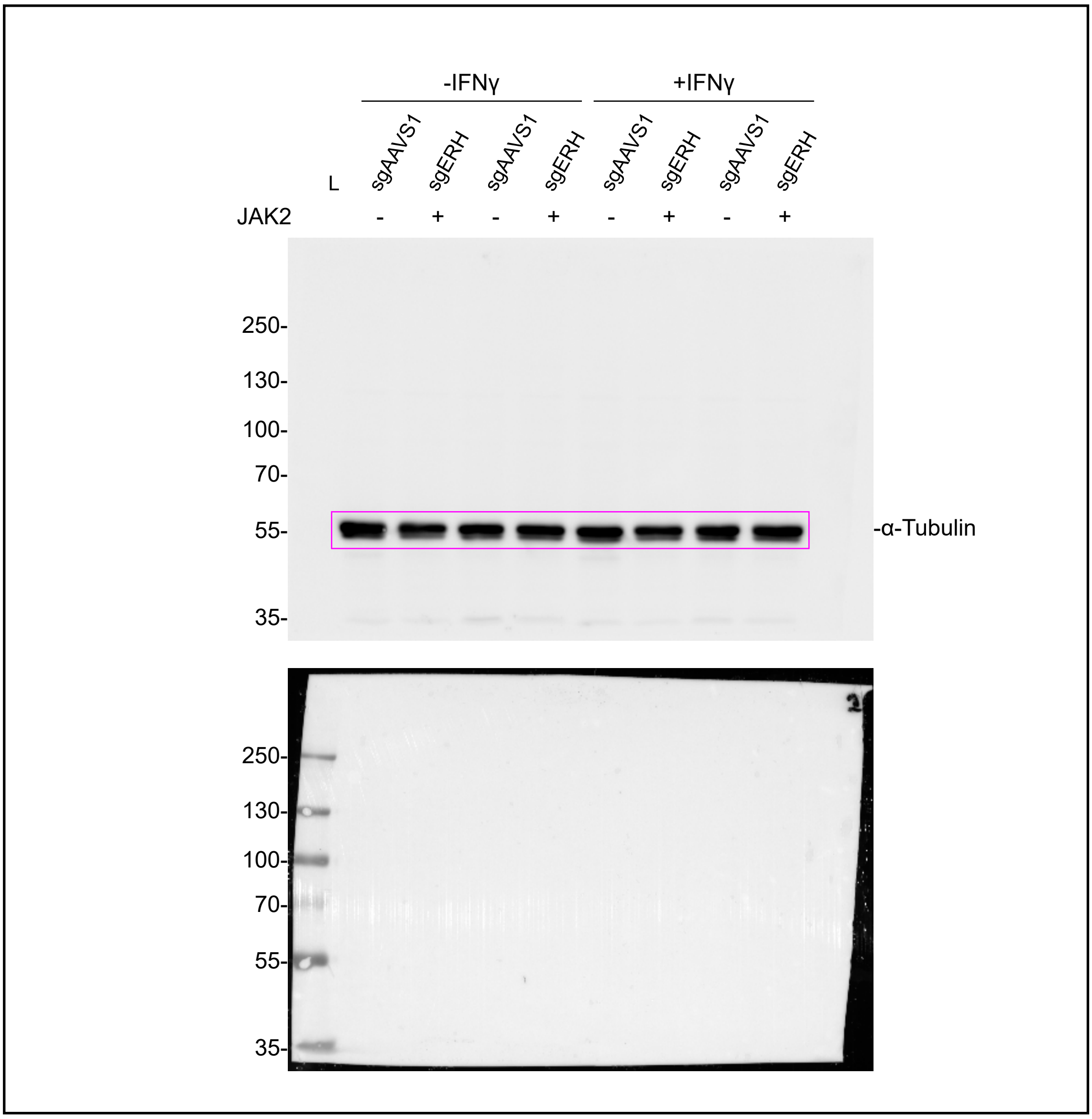

RKO, α-Tubulin (8% Gel 2), Replicate 2

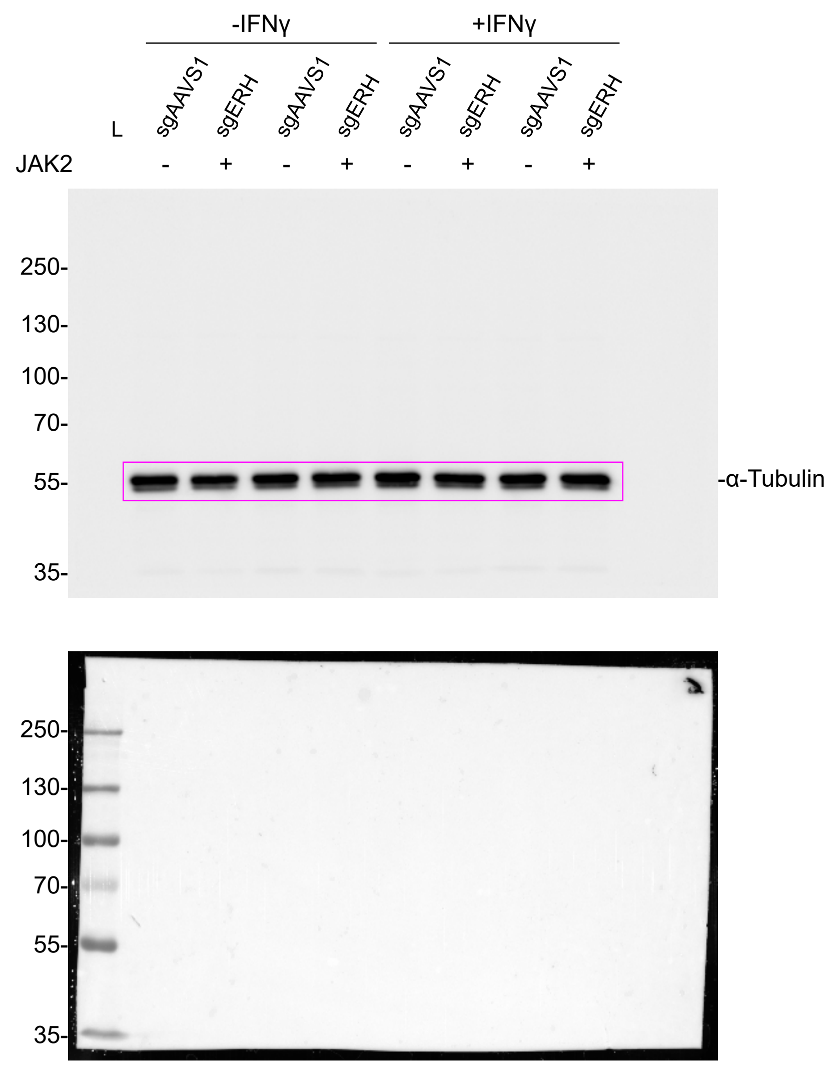

RKO, α-Tubulin (8% Gel 3), Replicate 3

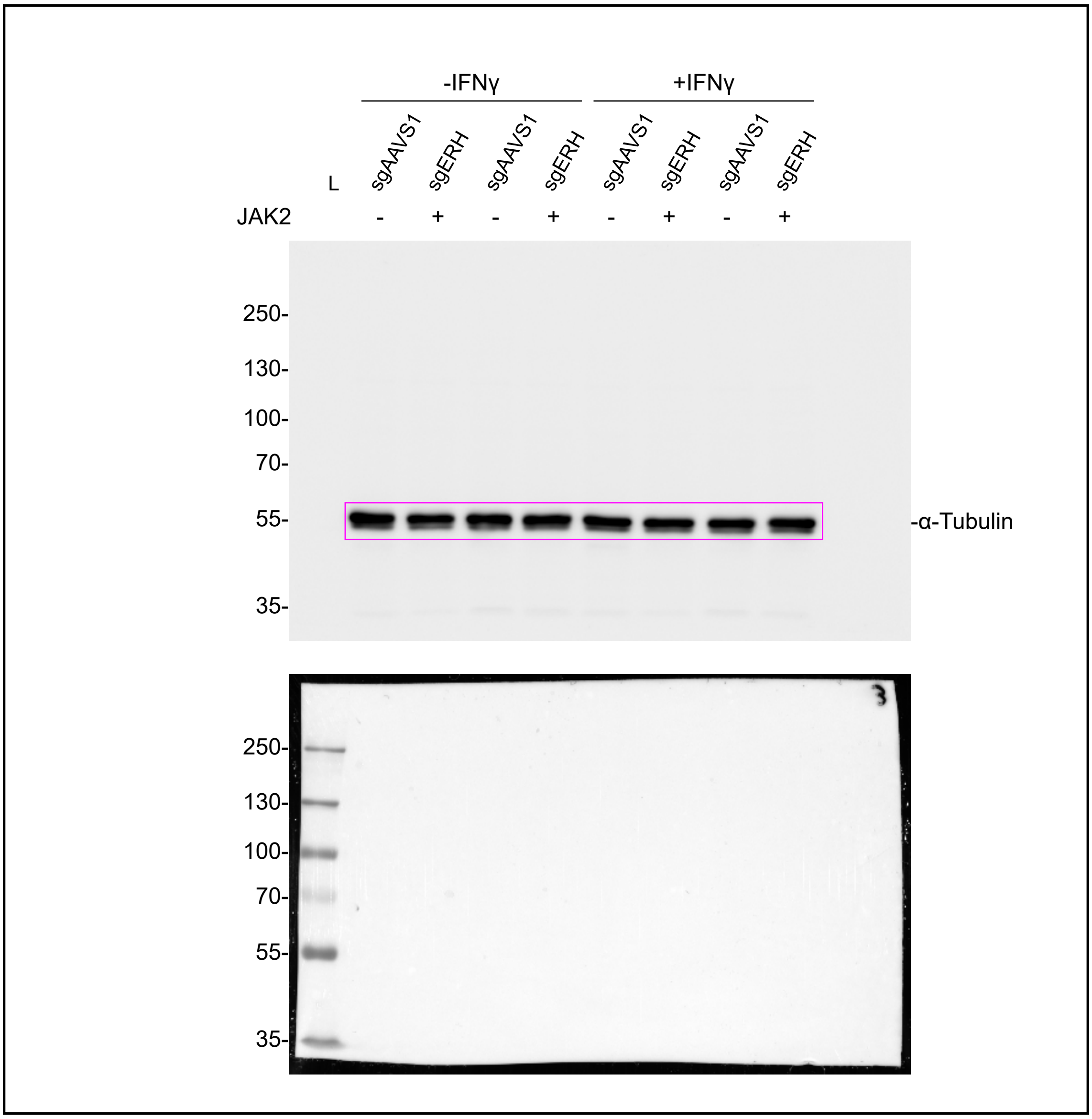

RKO, α-Tubulin (8% Gel 4), Replicate 1

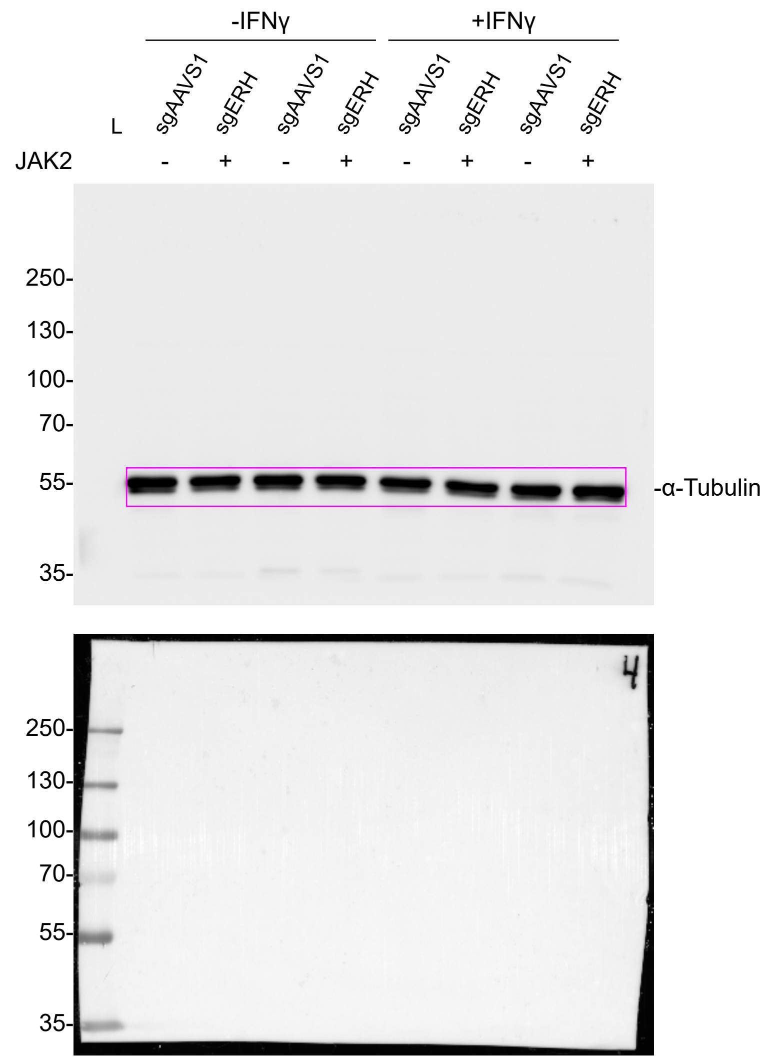

RKO, α-Tubulin (8% Gel 5), Replicate 2

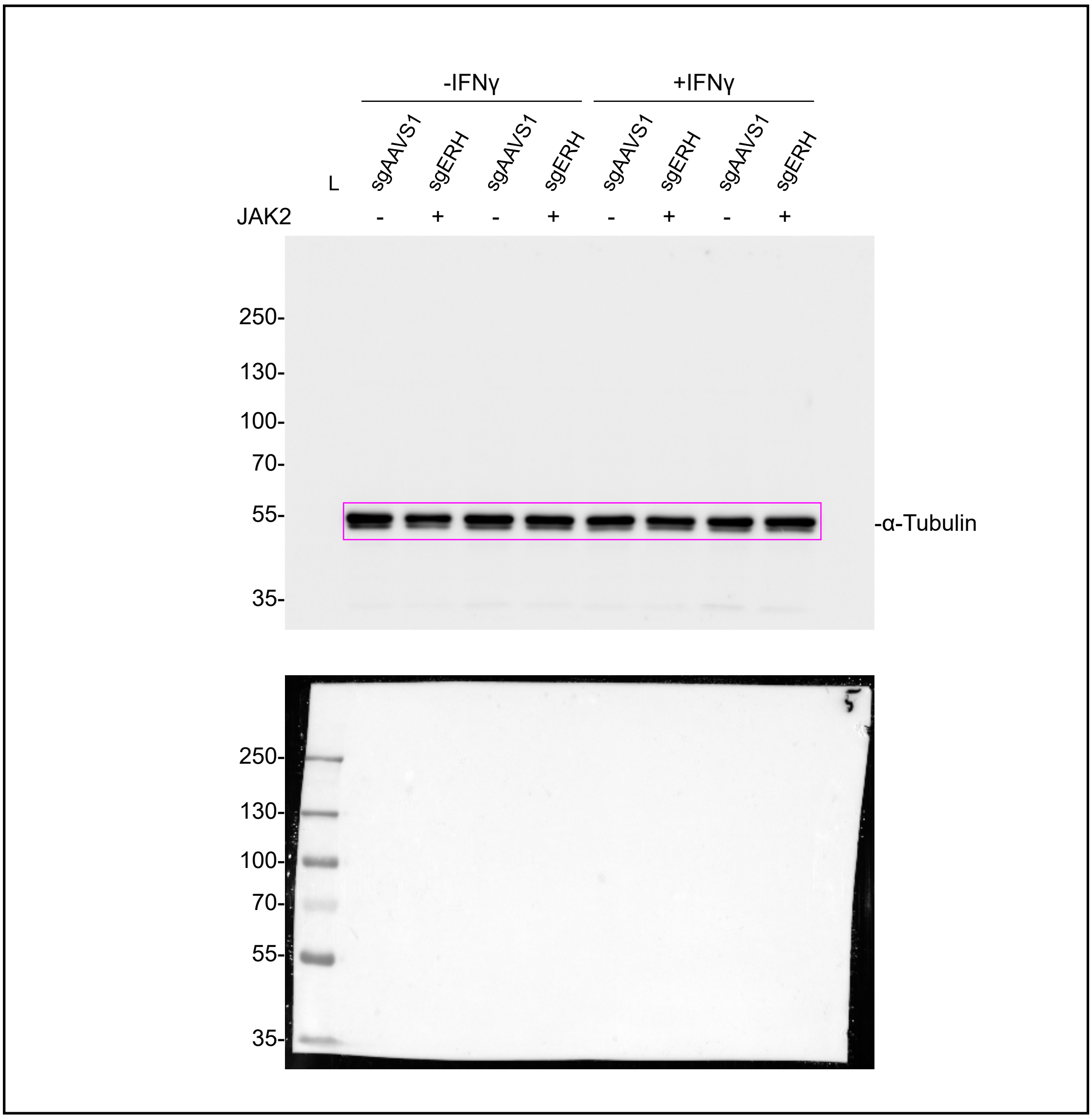

RKO, α-Tubulin (8% Gel 6), Replicate 3

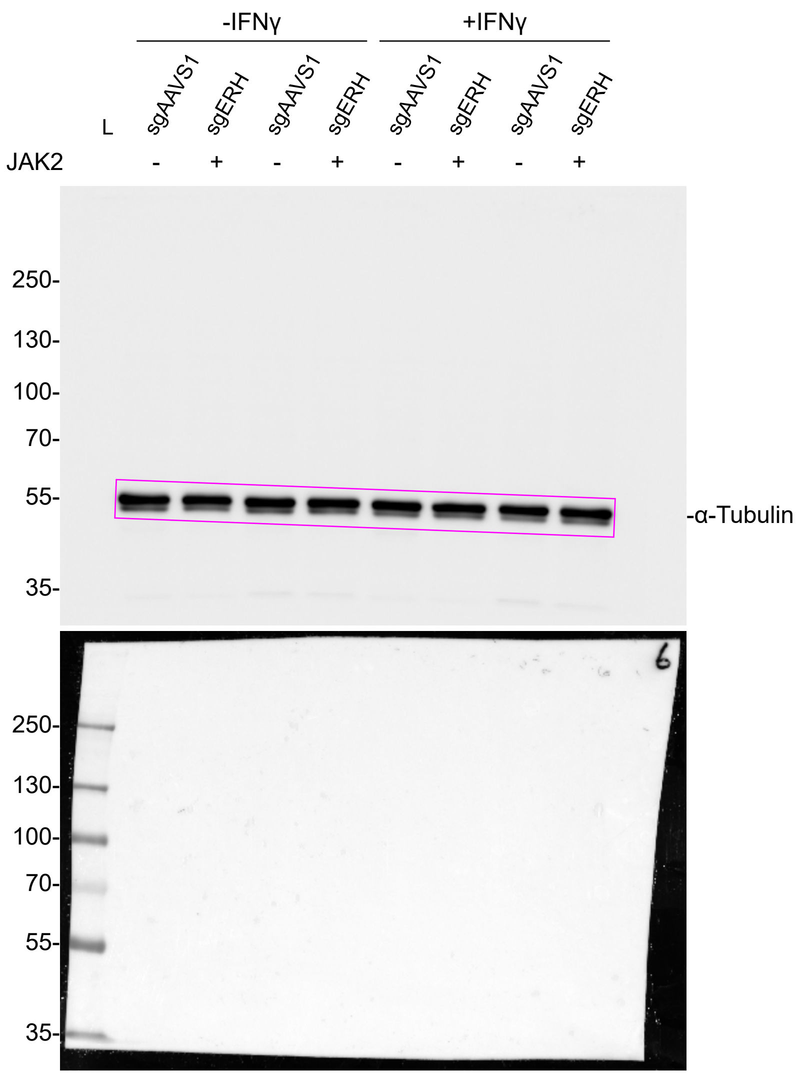

Souce_data_Fig_2E

Replicate 1

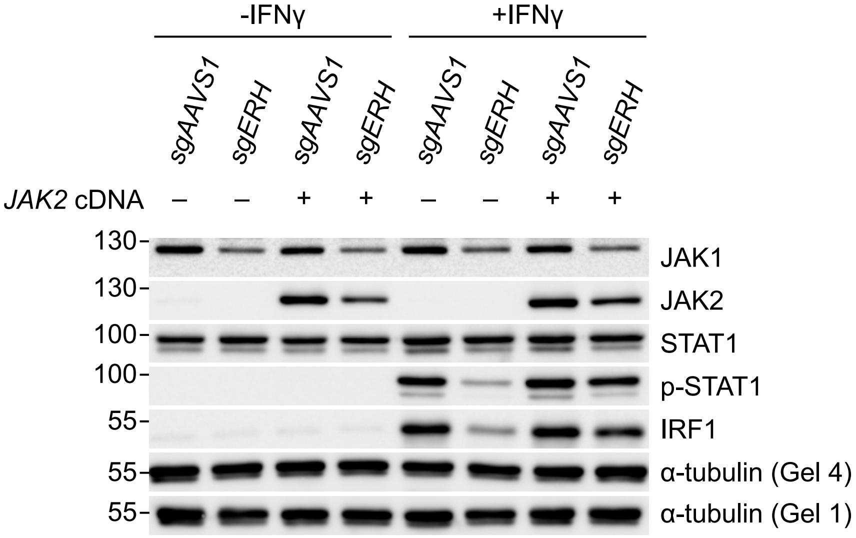

Replicate 2

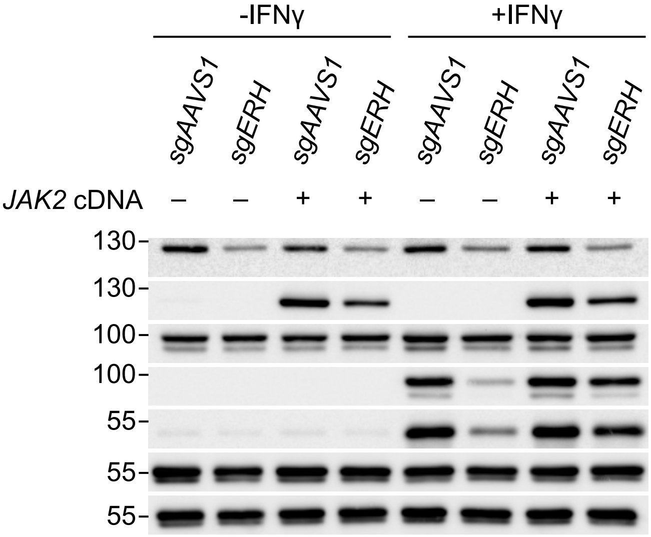

Replicate 3

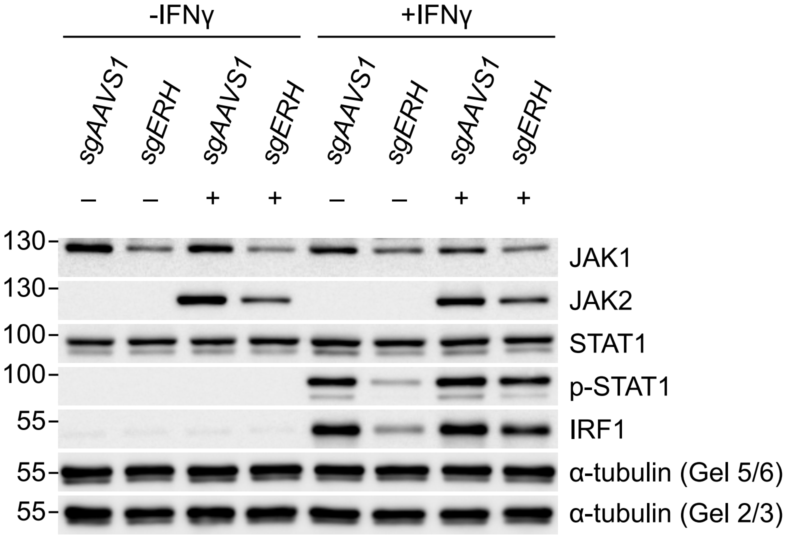

RKO, JAK2 (10% Gel 1)

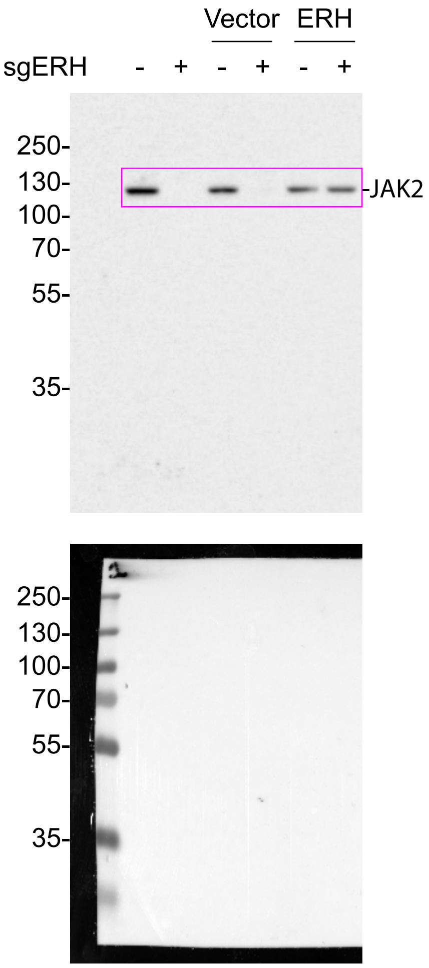

RKO, JAK2 (10% Gel 2)

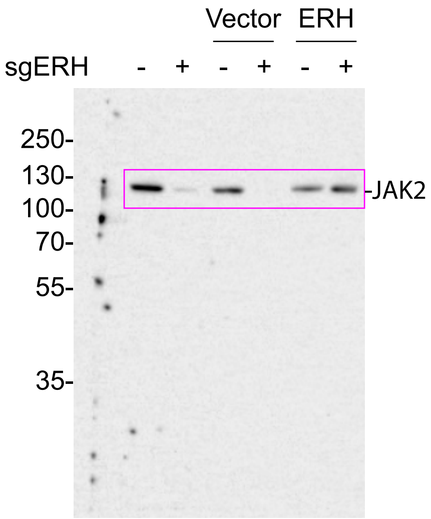

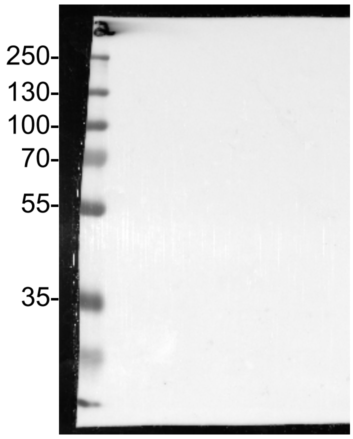

RKO, JAK2 (10% Gel 3)

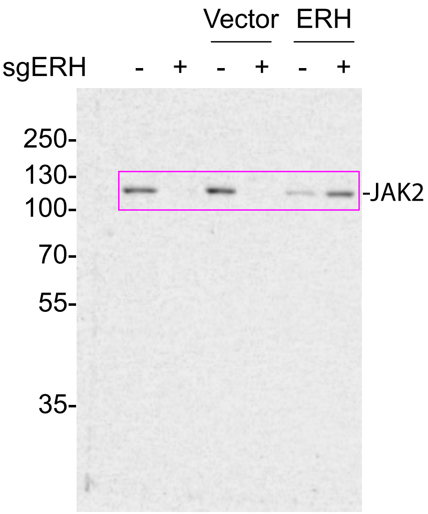

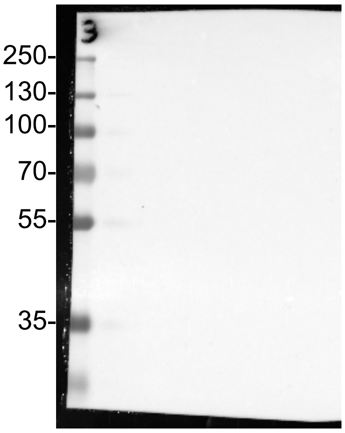

RKO, Vinculin (10% Gel 1)

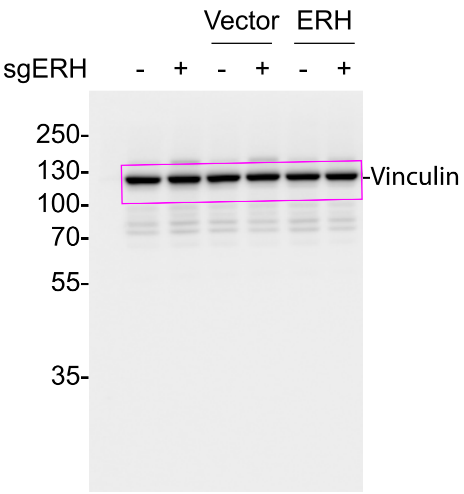

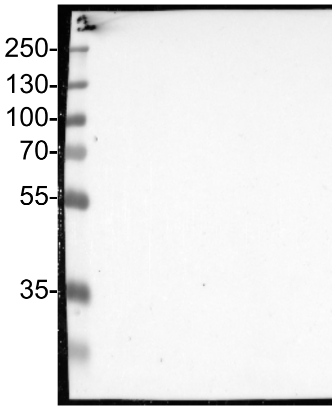

RKO, Vinculin (10% Gel 2)

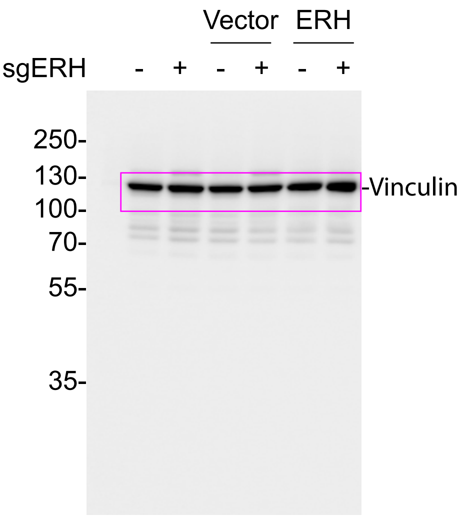

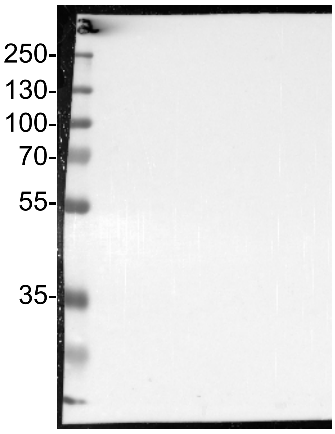

RKO, Vinculin (10% Gel 3)

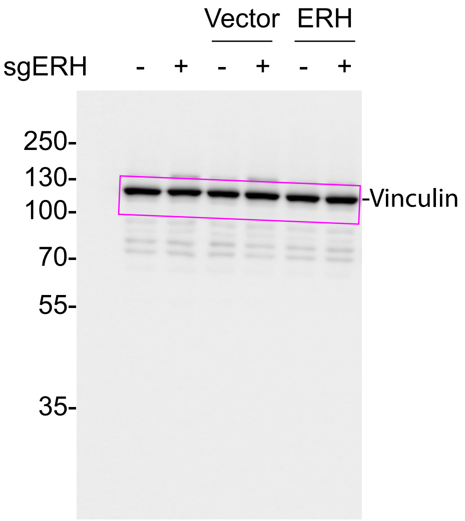

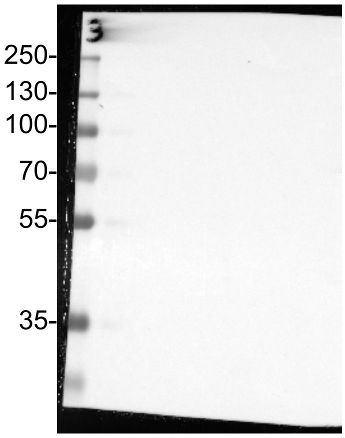

RKO, ERH (10% Gel 4)

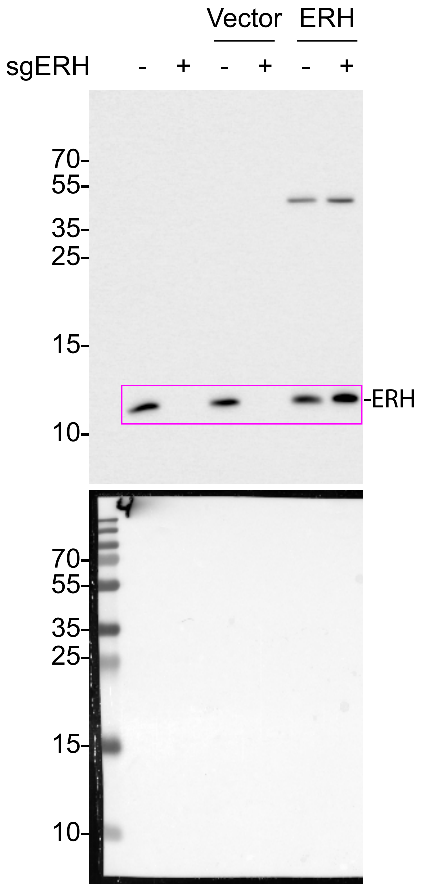

RKO, ERH (10% Gel 5)

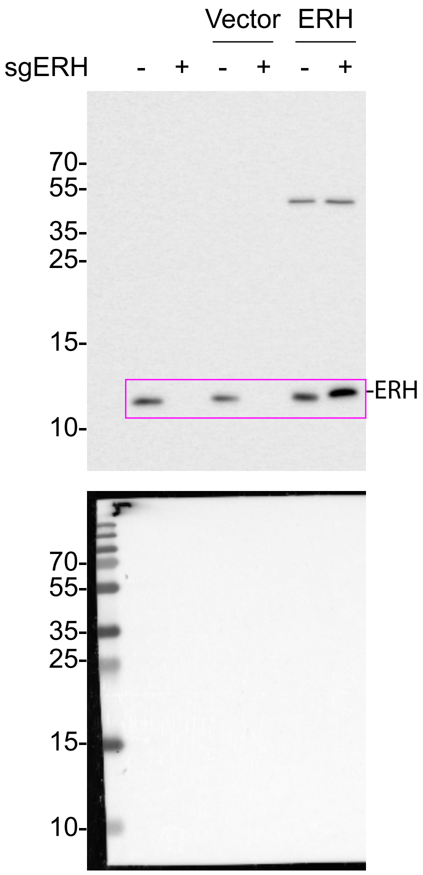

RKO, ERH (10% Gel 6)

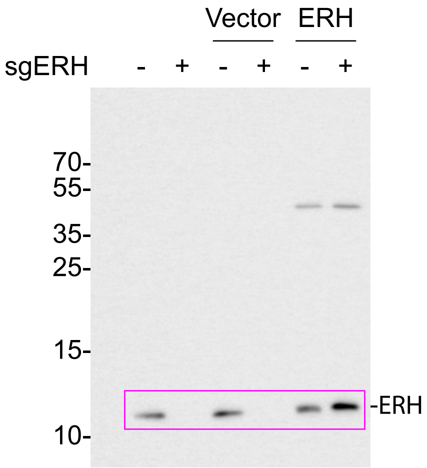

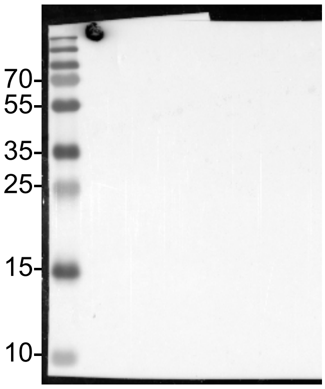

RKO, MYC-tag (10% Gel 4)

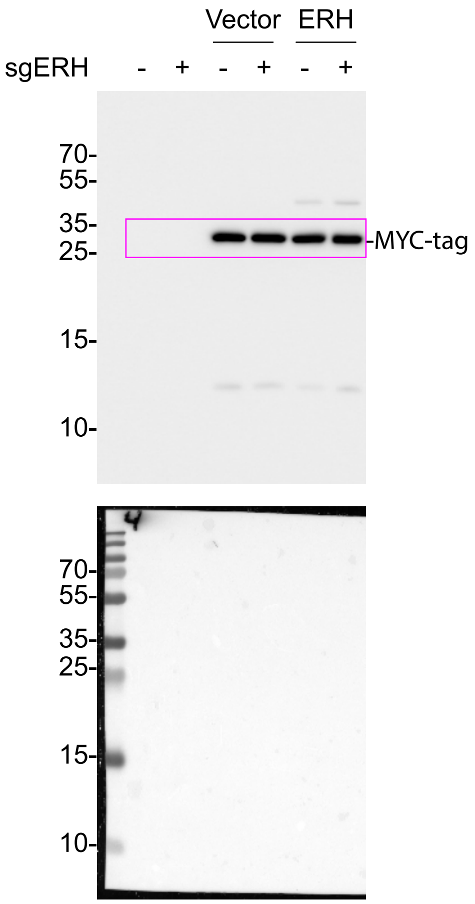

Souce_data_Fig_2G

RKO, MYC-tag (10% Gel 5)

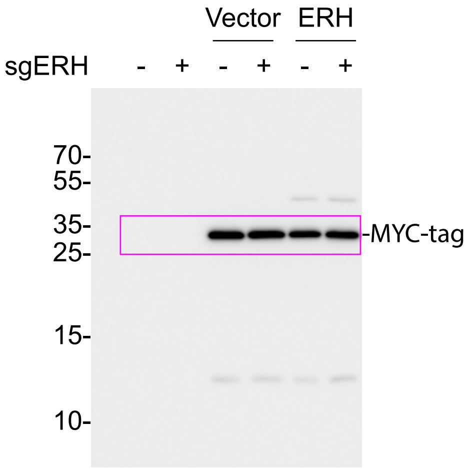

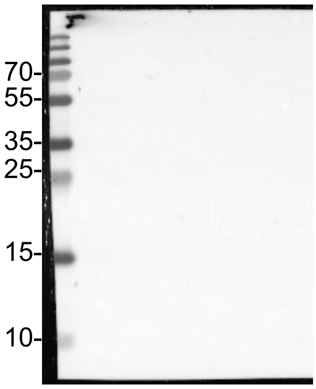

RKO, MYC-tag (10% Gel 6)

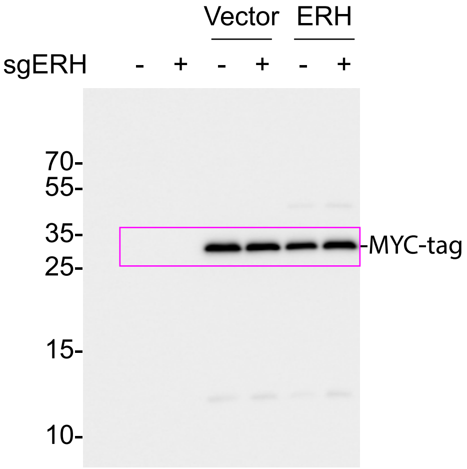

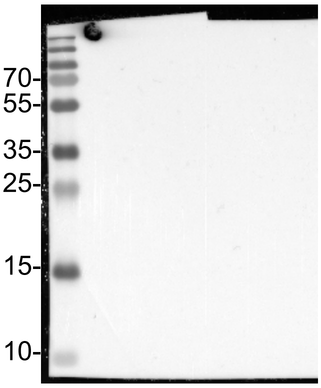

Replicate 1

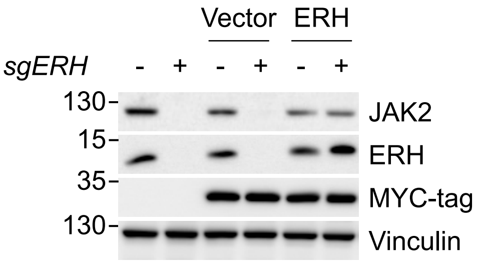

Replicate 2

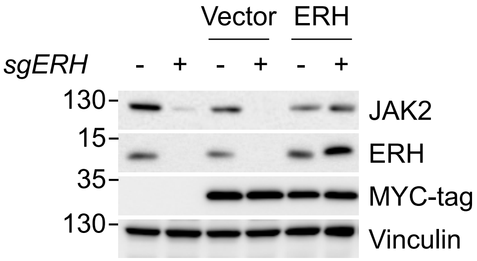

Replicate 3

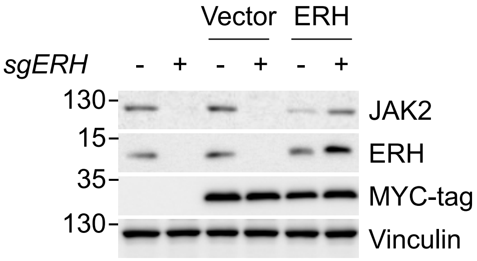

RKO, ERH (15% Gel)

Replicate 1

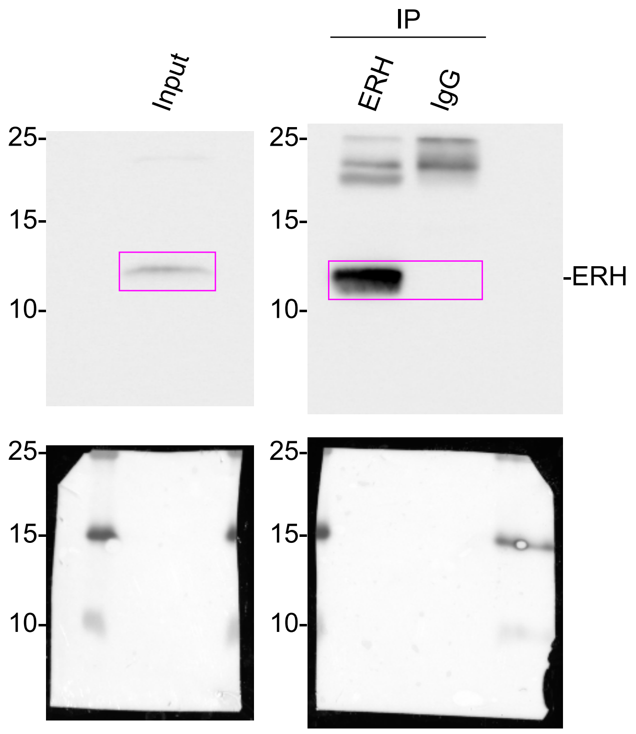

Replicate 2

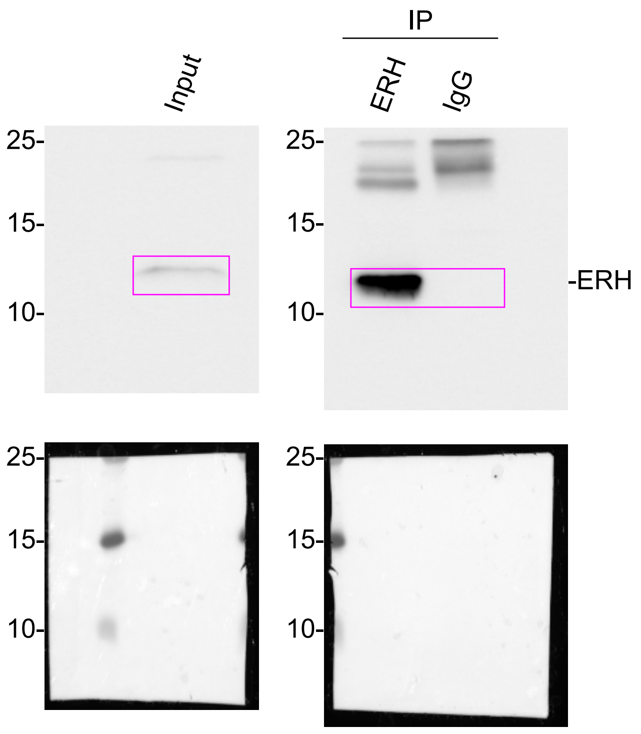

RKO, ALYREF (15% Gel)

Replicate 1

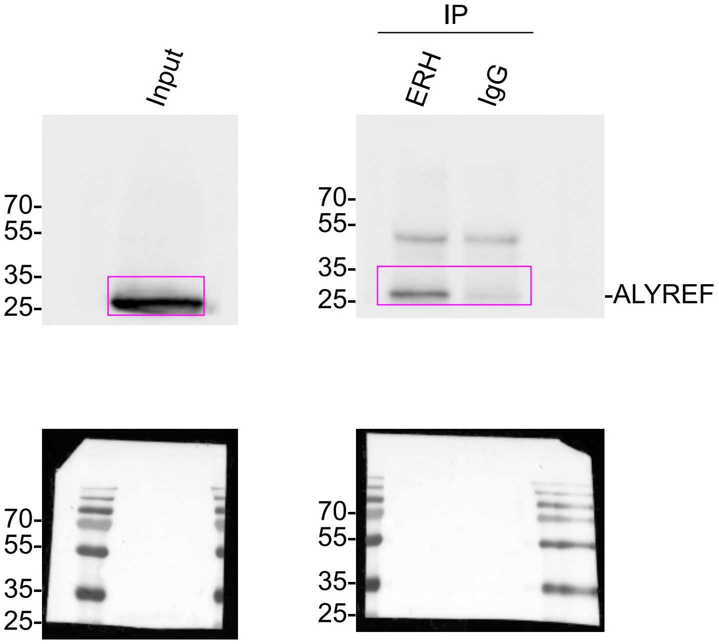

Replicate 2

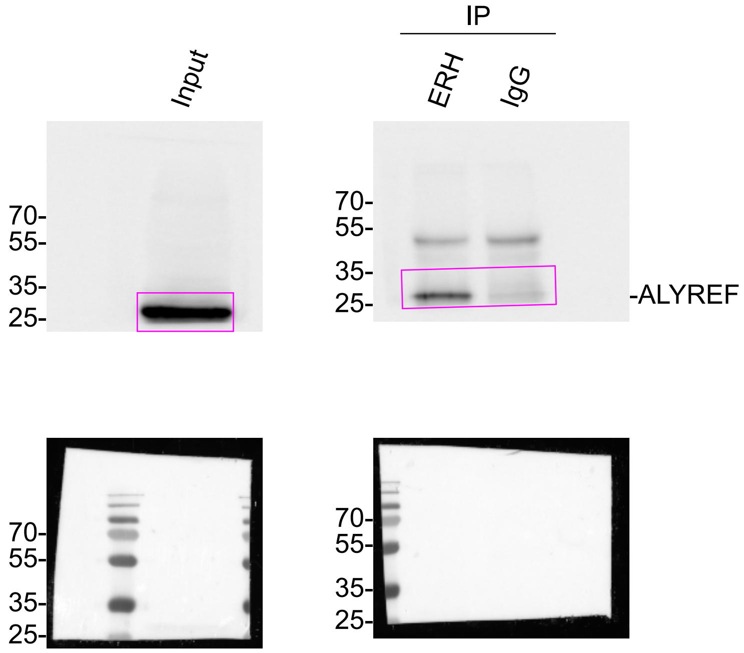

RKO, MAGOH (15% Gel)

Replicate 1

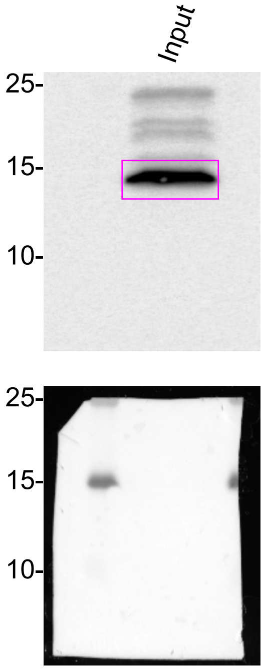

Replicate 2

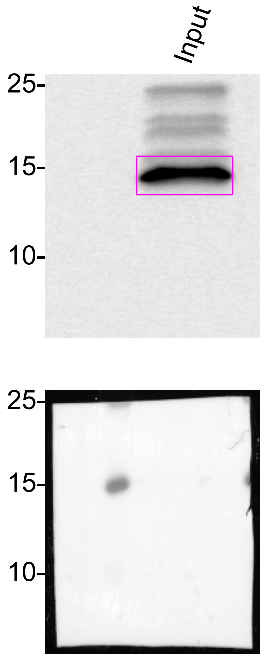

RKO, MAGOH (15% Gel)

Replicate 1

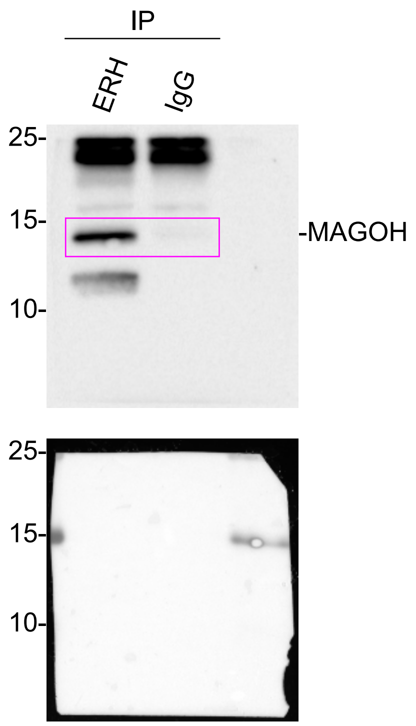

Replicate 2

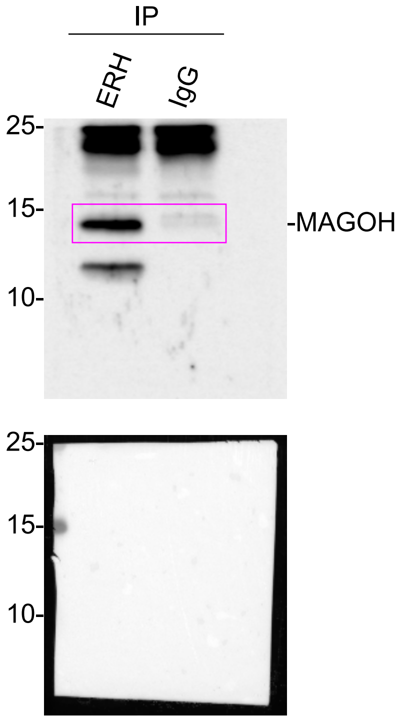

RKO, SRSF1 (15% Gel)

Replicate 1

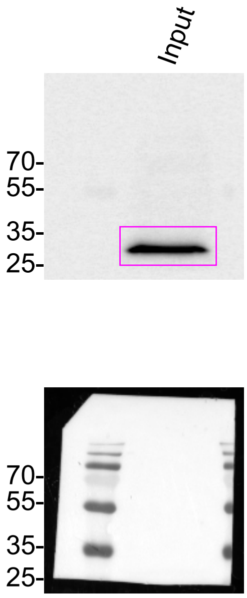

Replicate 2

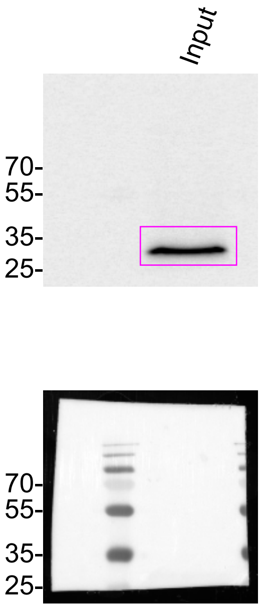

RKO, SRSF1 (15% Gel)

Replicate 1

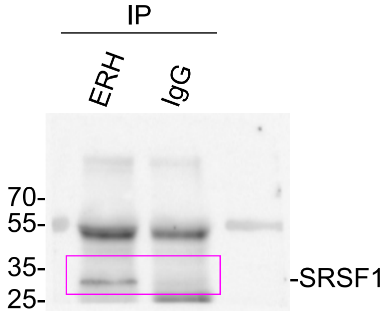

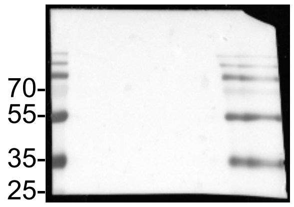

Replicate 2

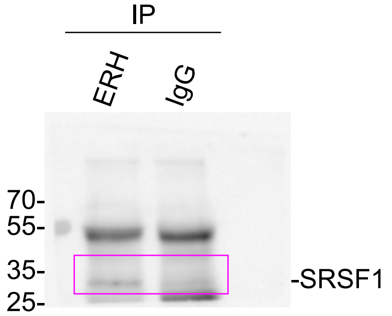

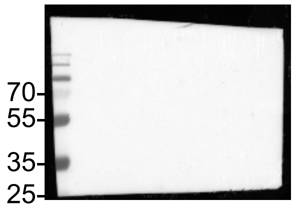

RKO, α-Tubulin (15% Gel)

Replicate 1

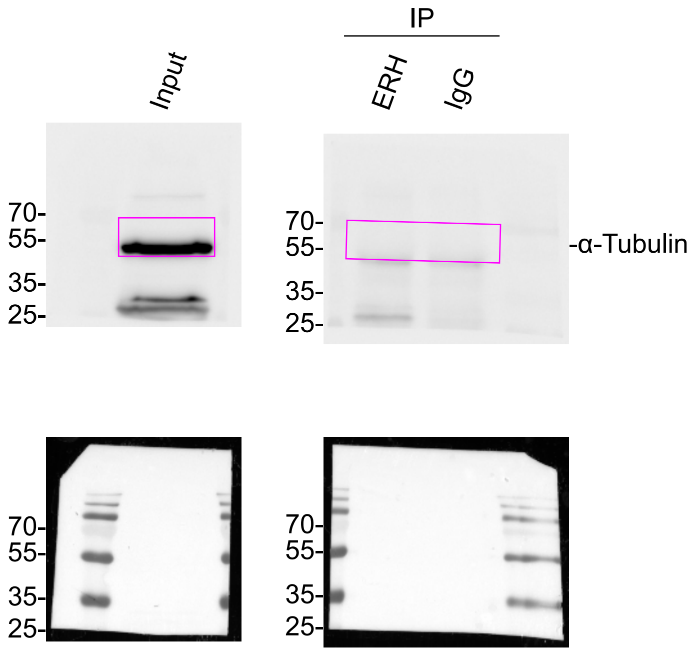

Replicate 2

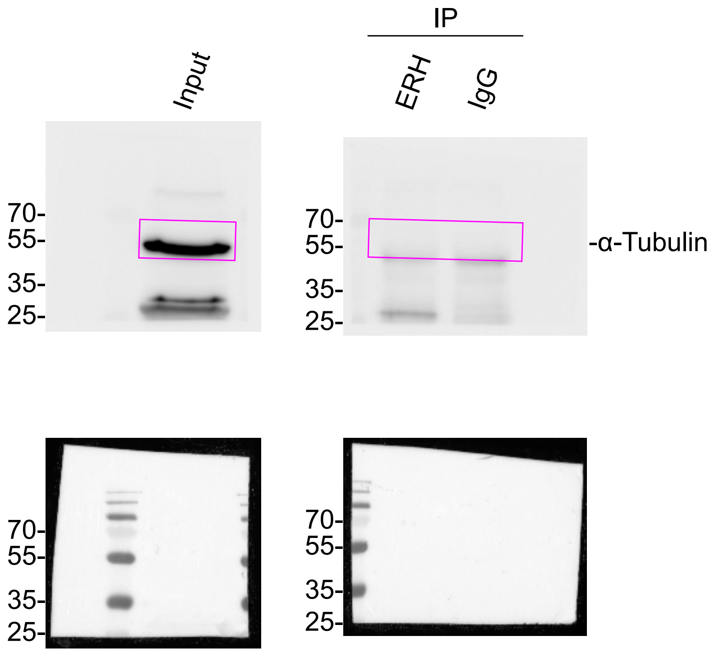

Souce_data_Fig_3B

Replicate 1

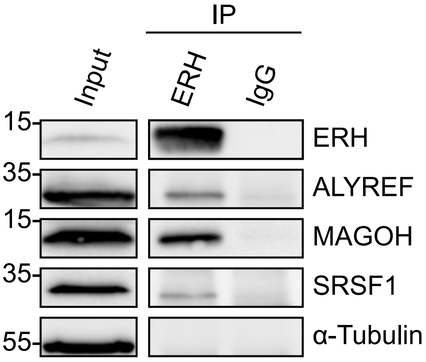

Replicate 2

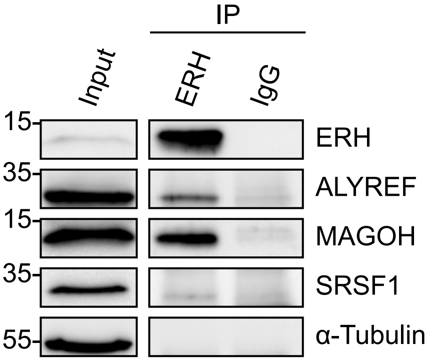

Souce_data_Fig_3E

Replicate 1

RKO, JAK2 (8% Gel)

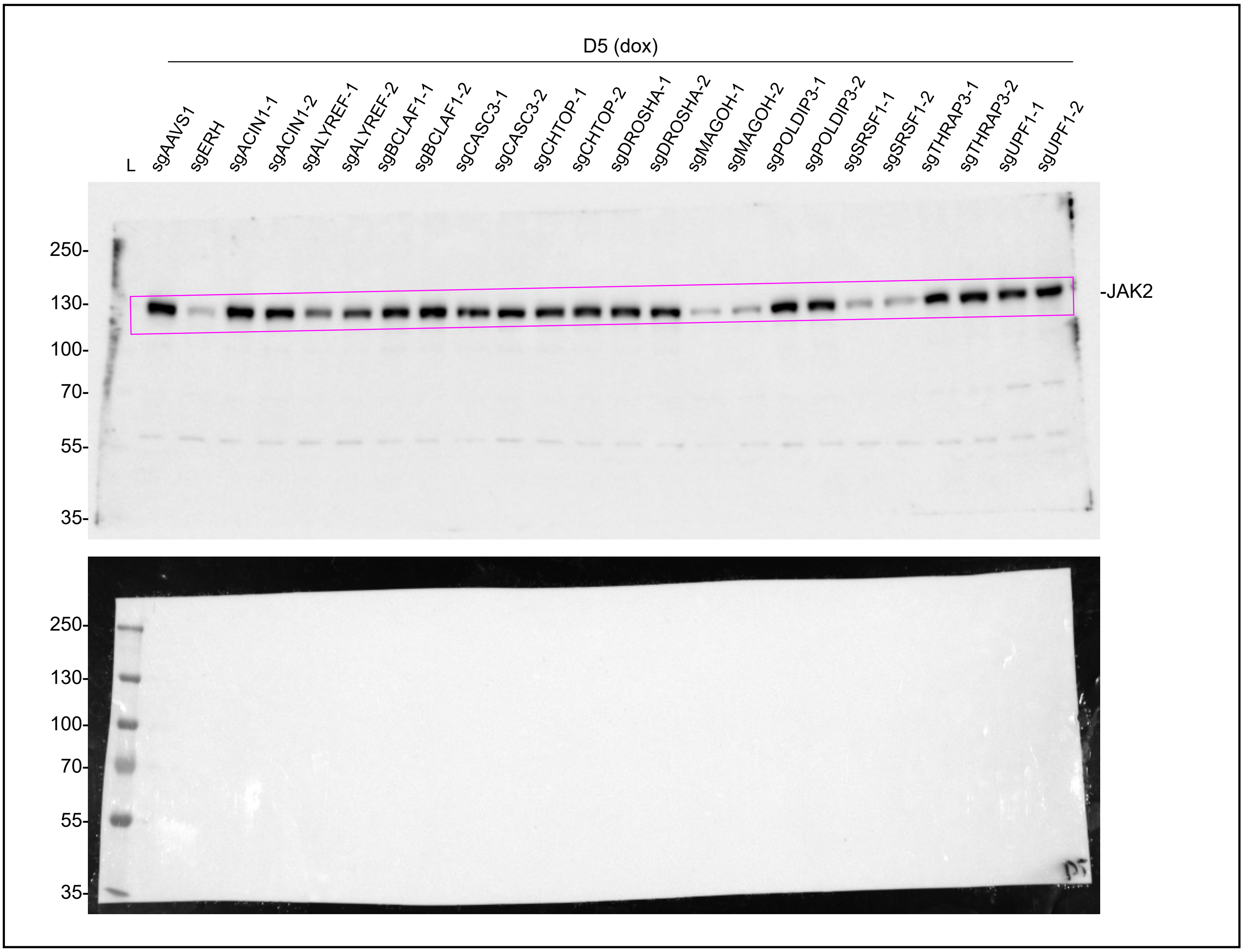

Souce_data_Fig_3E

Replicate 1

RKO, STAT1 (8% Gel)

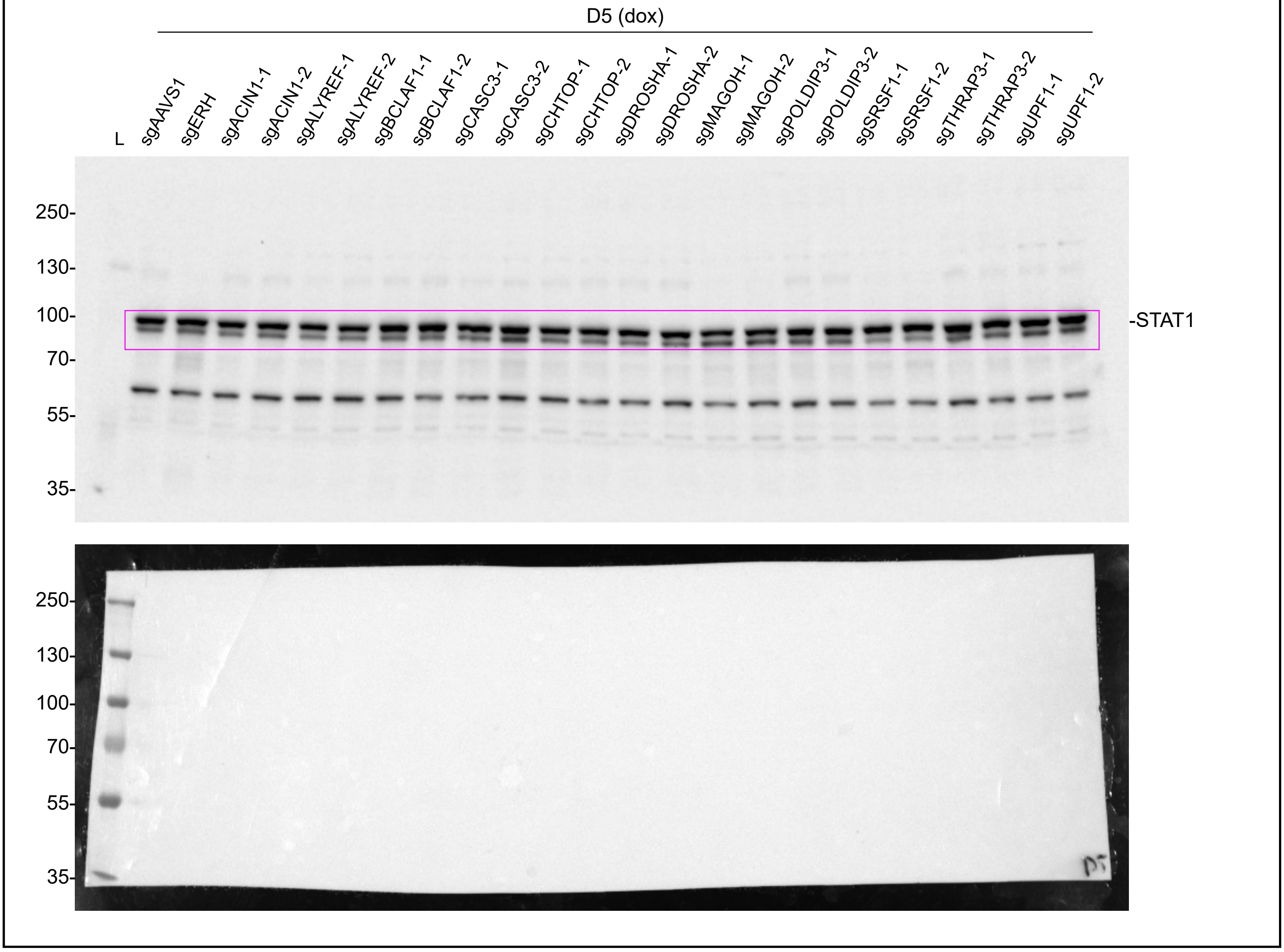

Replicate 1

RKO, Vinculin (8% Gel)

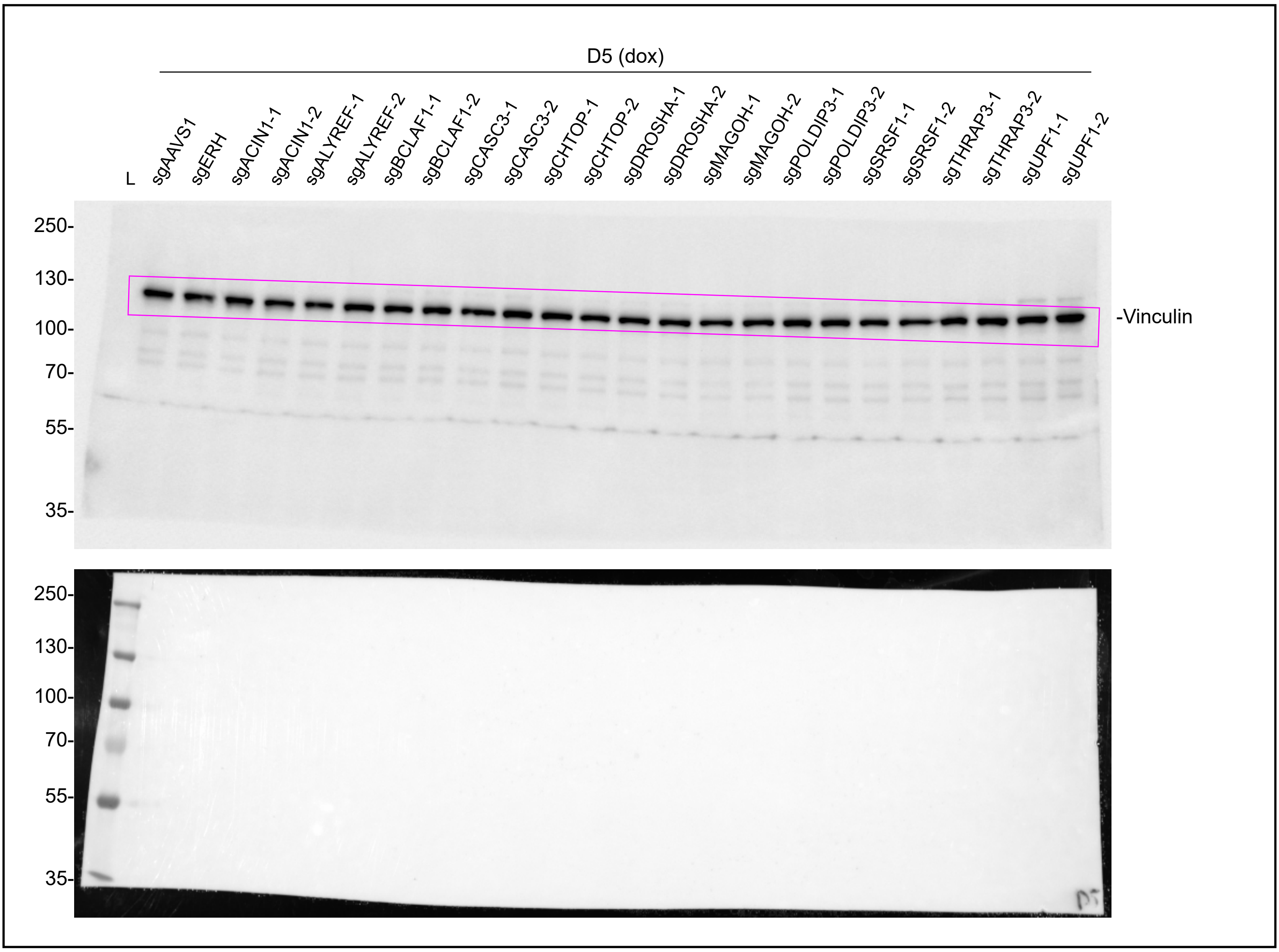

Souce_data_Fig_3E

Replicate 2

RKO, JAK2 (8% Gel 1)

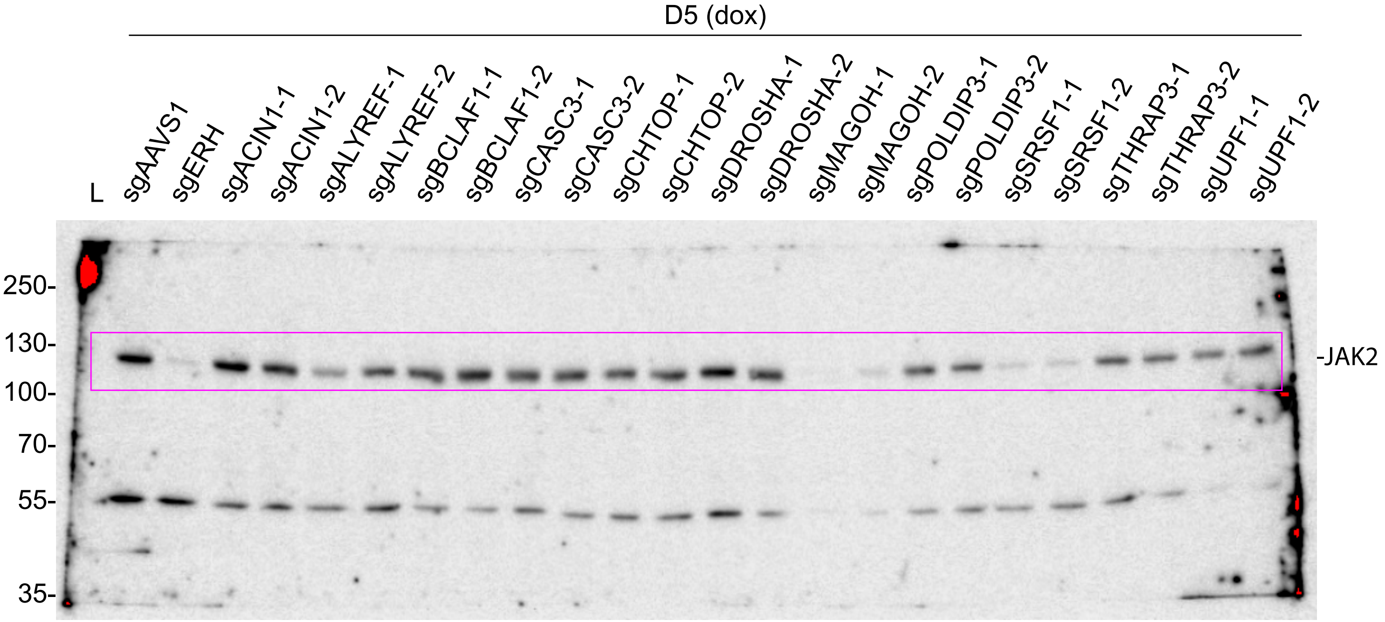

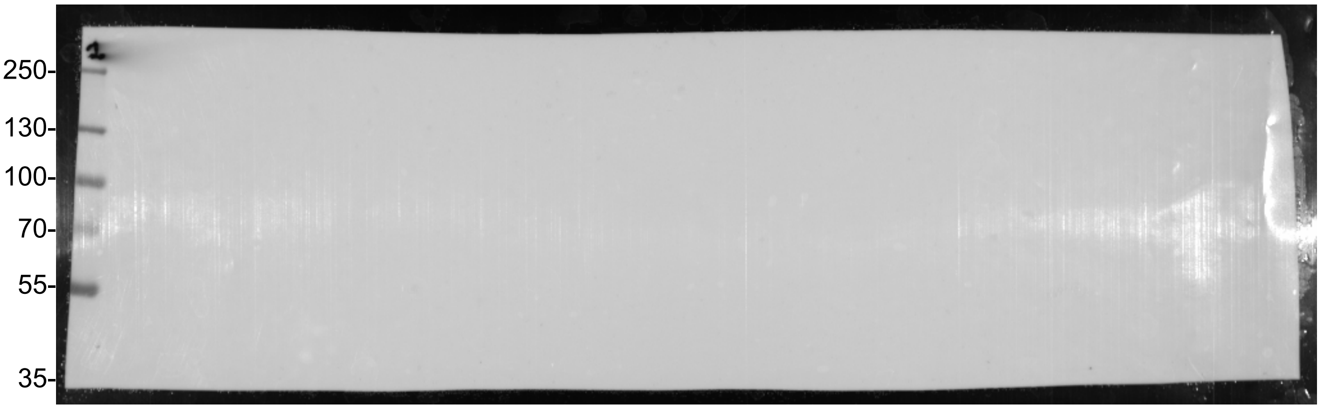

Souce_data_Fig_3E

Replicate 3

RKO, JAK2 (8% Gel 2)

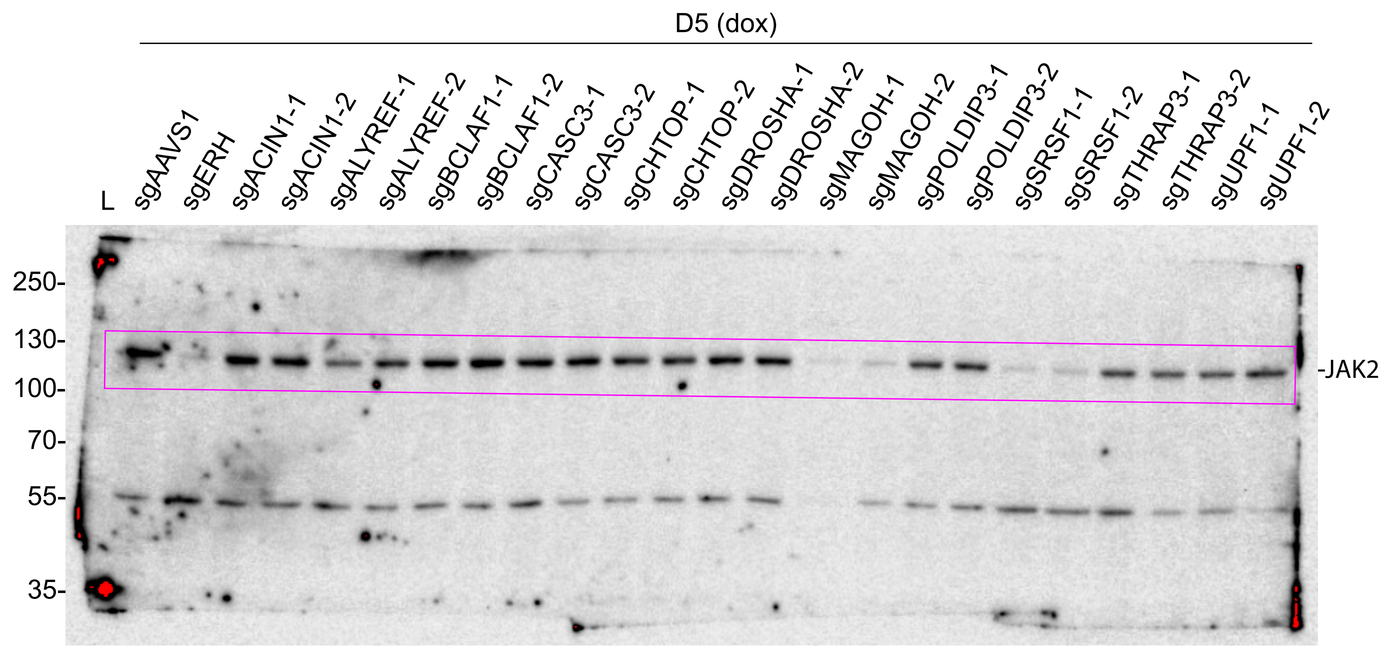

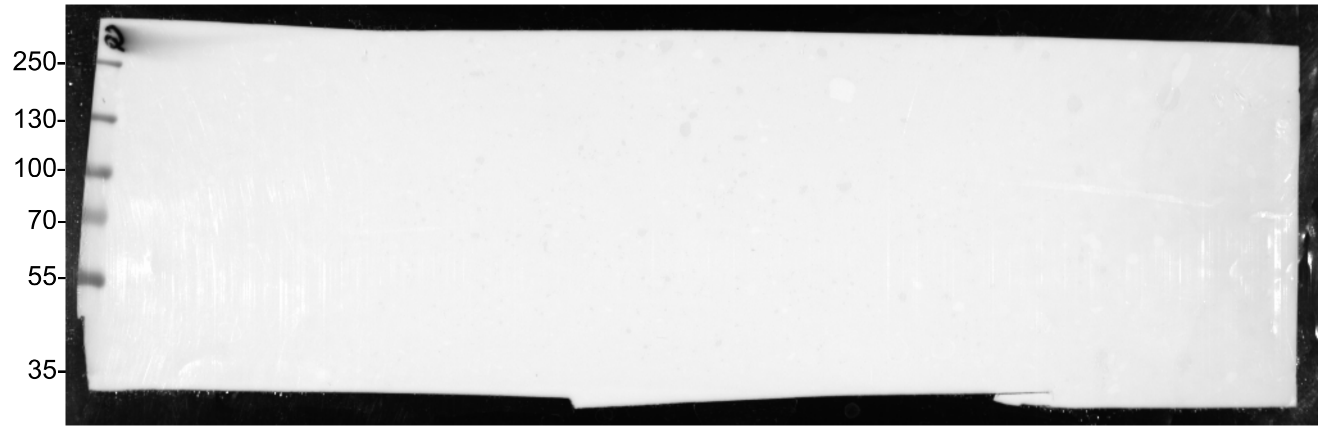

Souce_data_Fig_3E

Replicate 4

RKO, JAK2 (8% Gel 3)

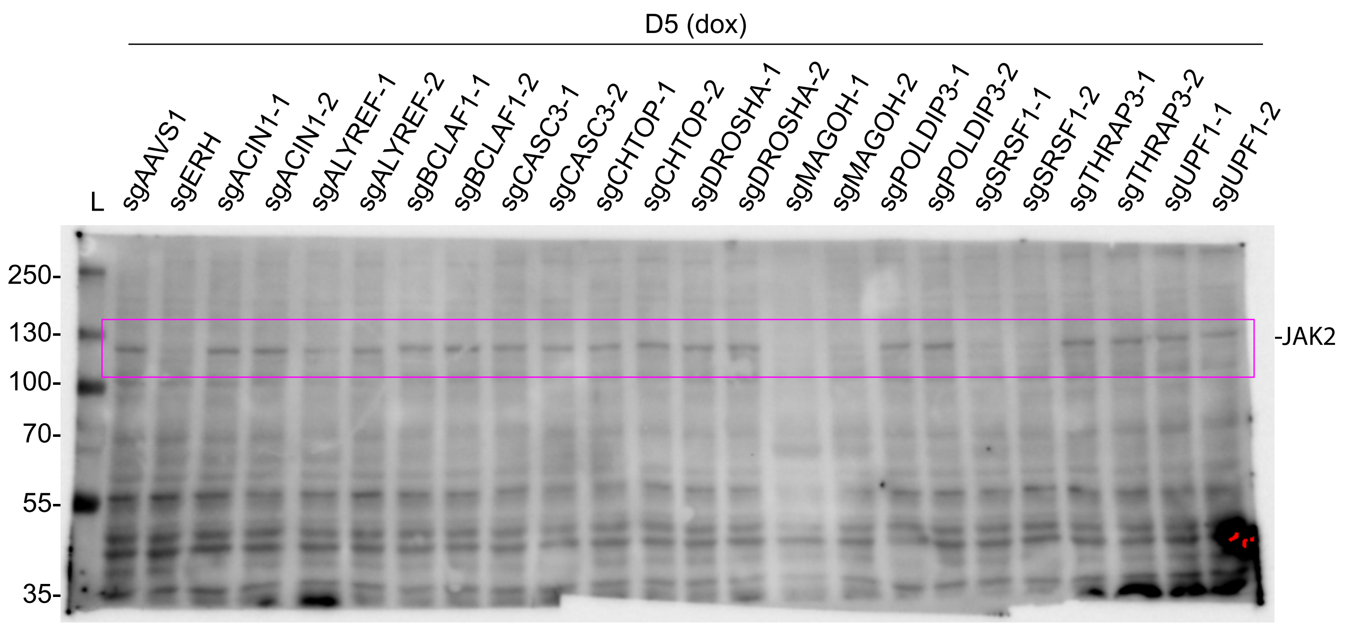

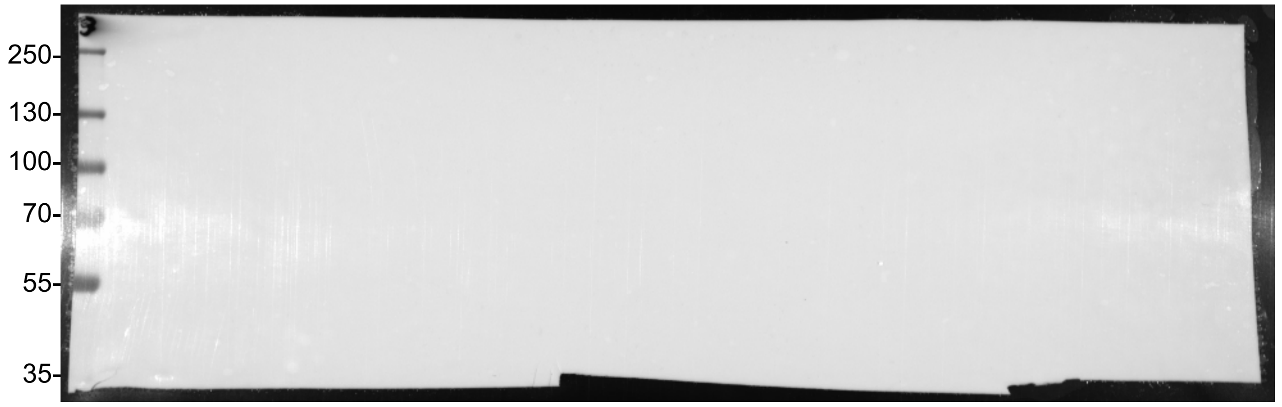

Souce_data_Fig_3E

Replicate 2

RKO, STAT1 (8% Gel 1)

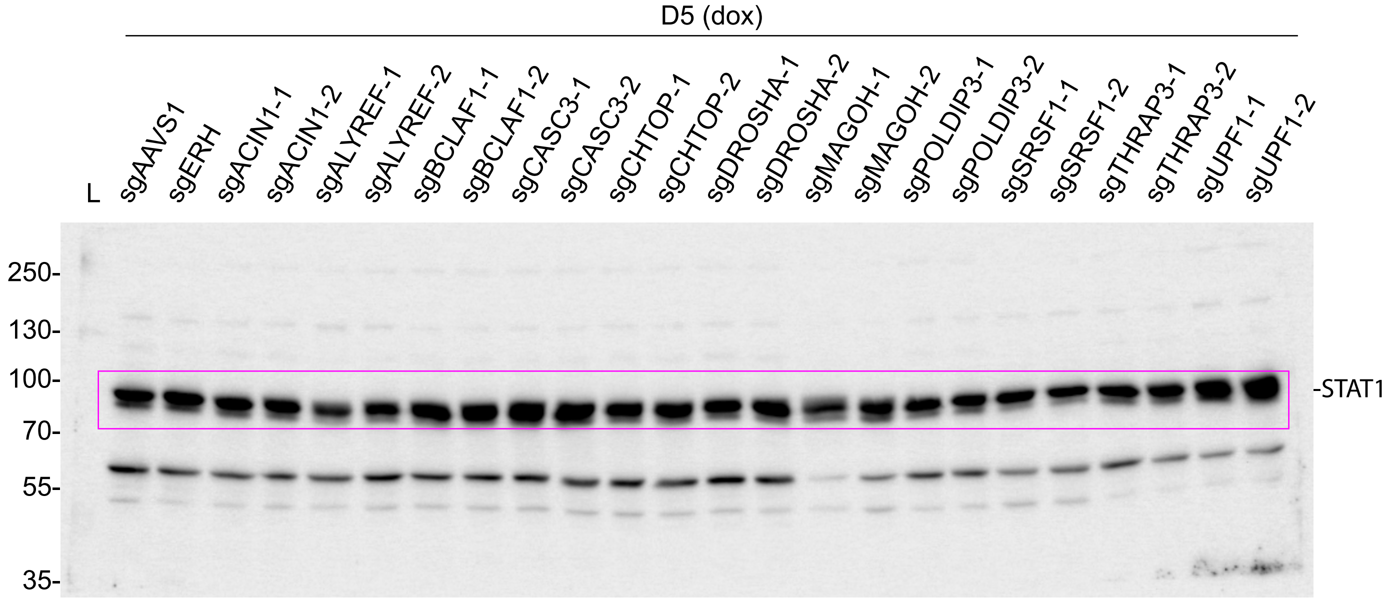

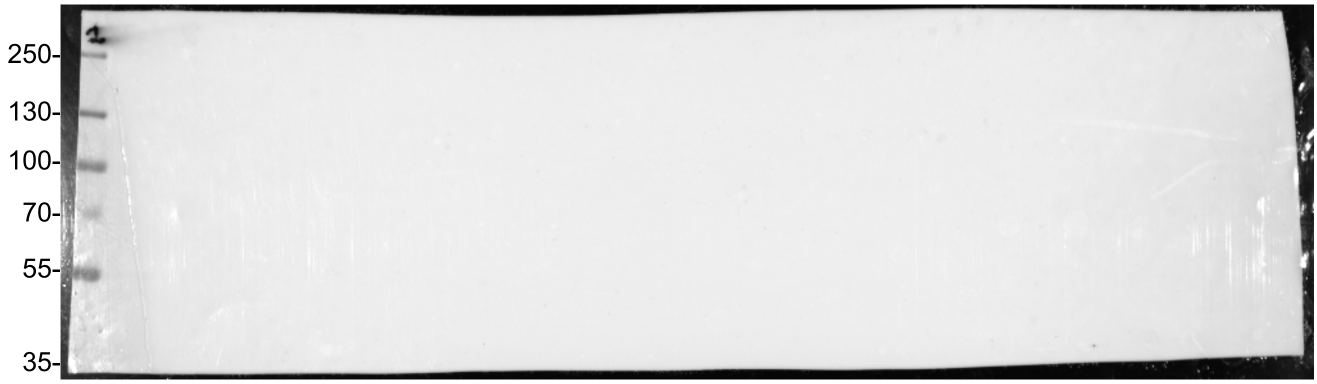

Souce_data_Fig_3E

Replicate 3

RKO, STAT1 (8% Gel 2)

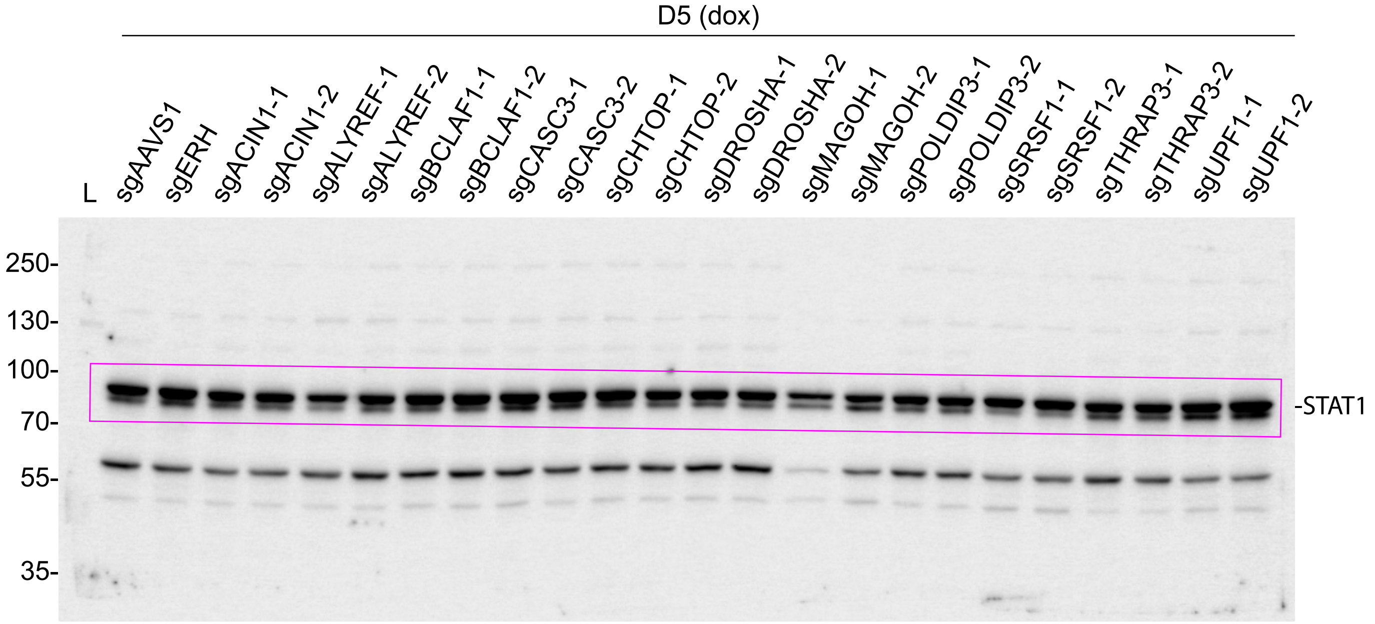

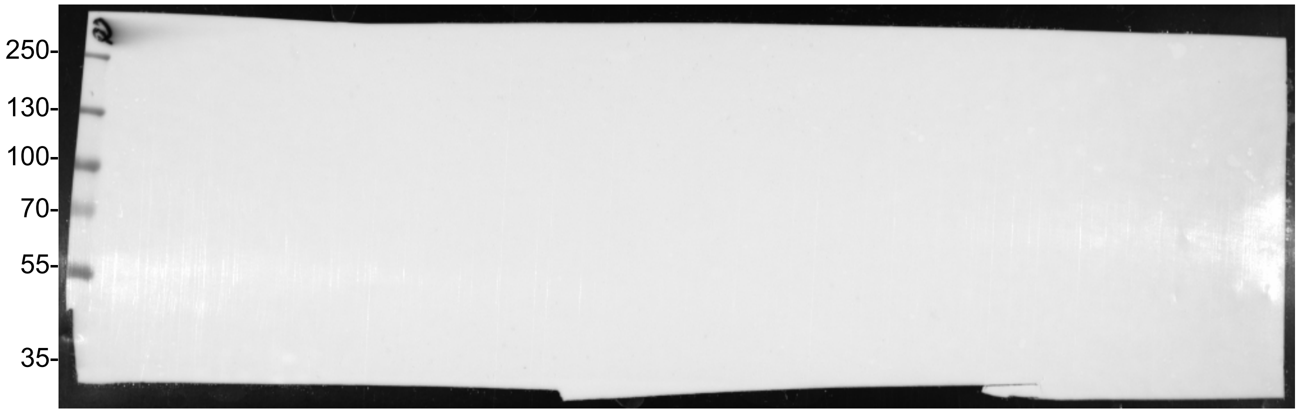

Souce_data_Fig_3E

Replicate 4

RKO, STAT1 (8% Gel 3)

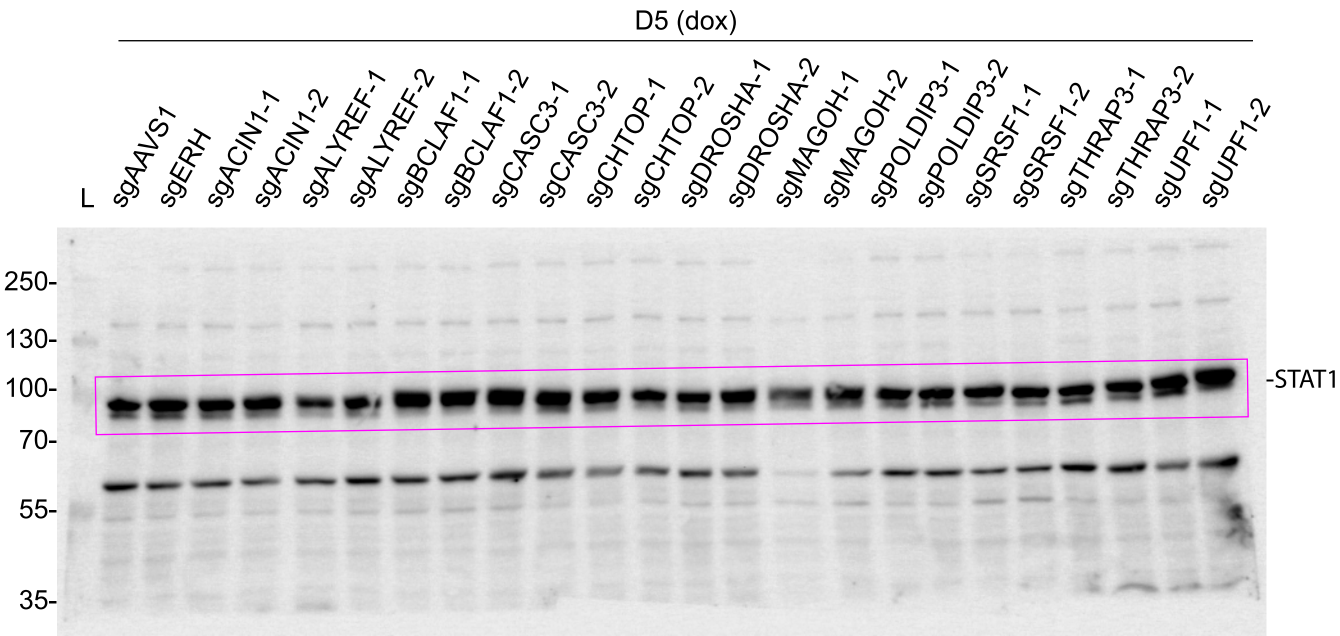

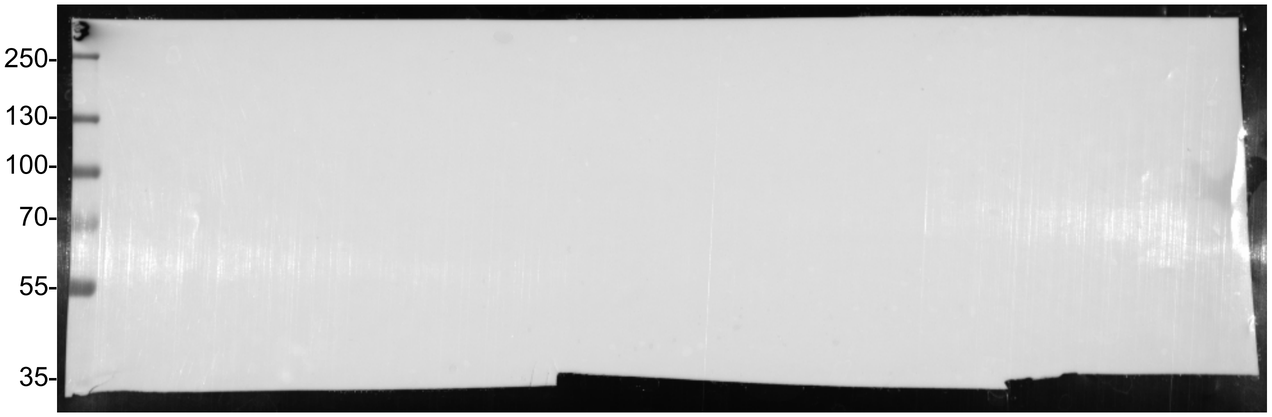

Souce_data_Fig_3E

Replicate 2

RKO, Vinculin (8% Gel 1)

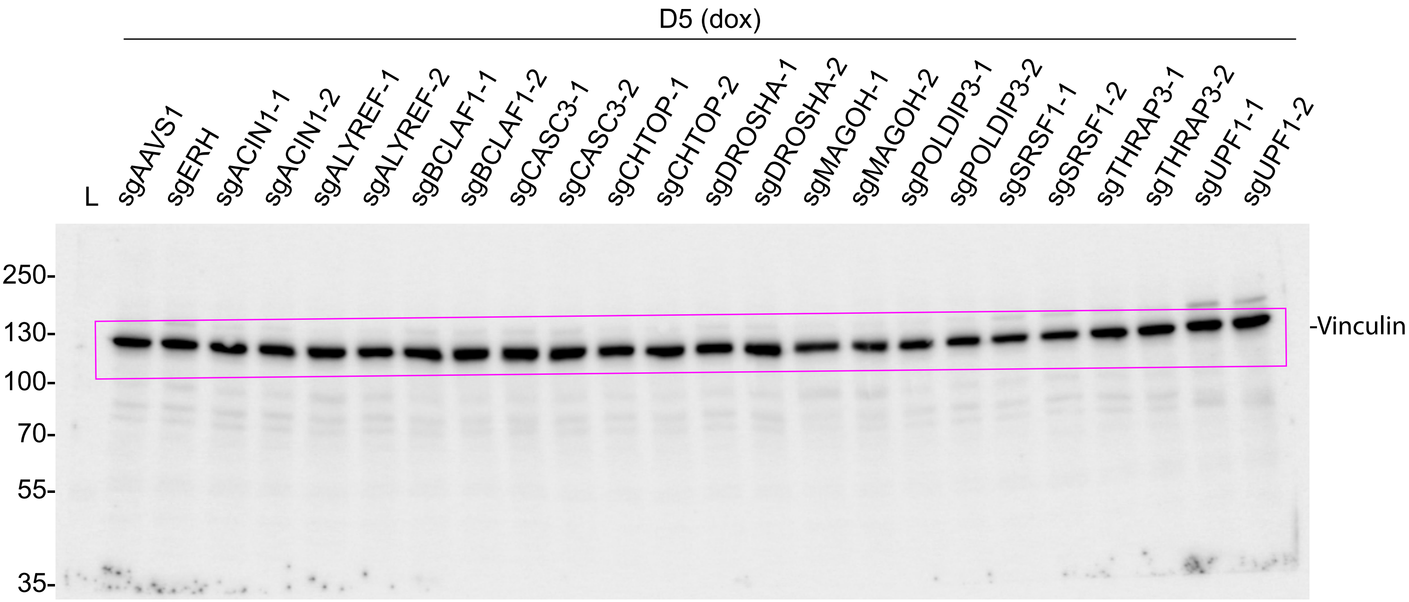

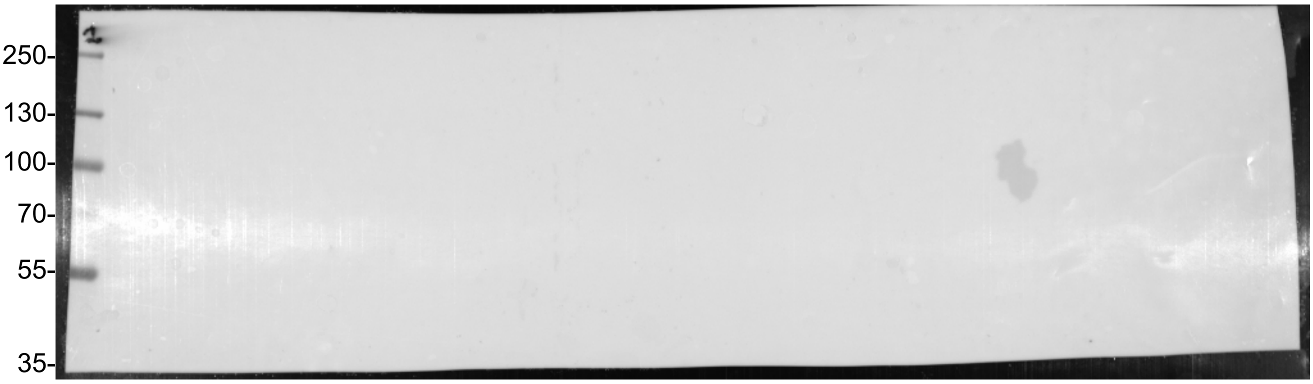

Souce_data_Fig_3E

Replicate 3

RKO, Vinculin (8% Gel 2)

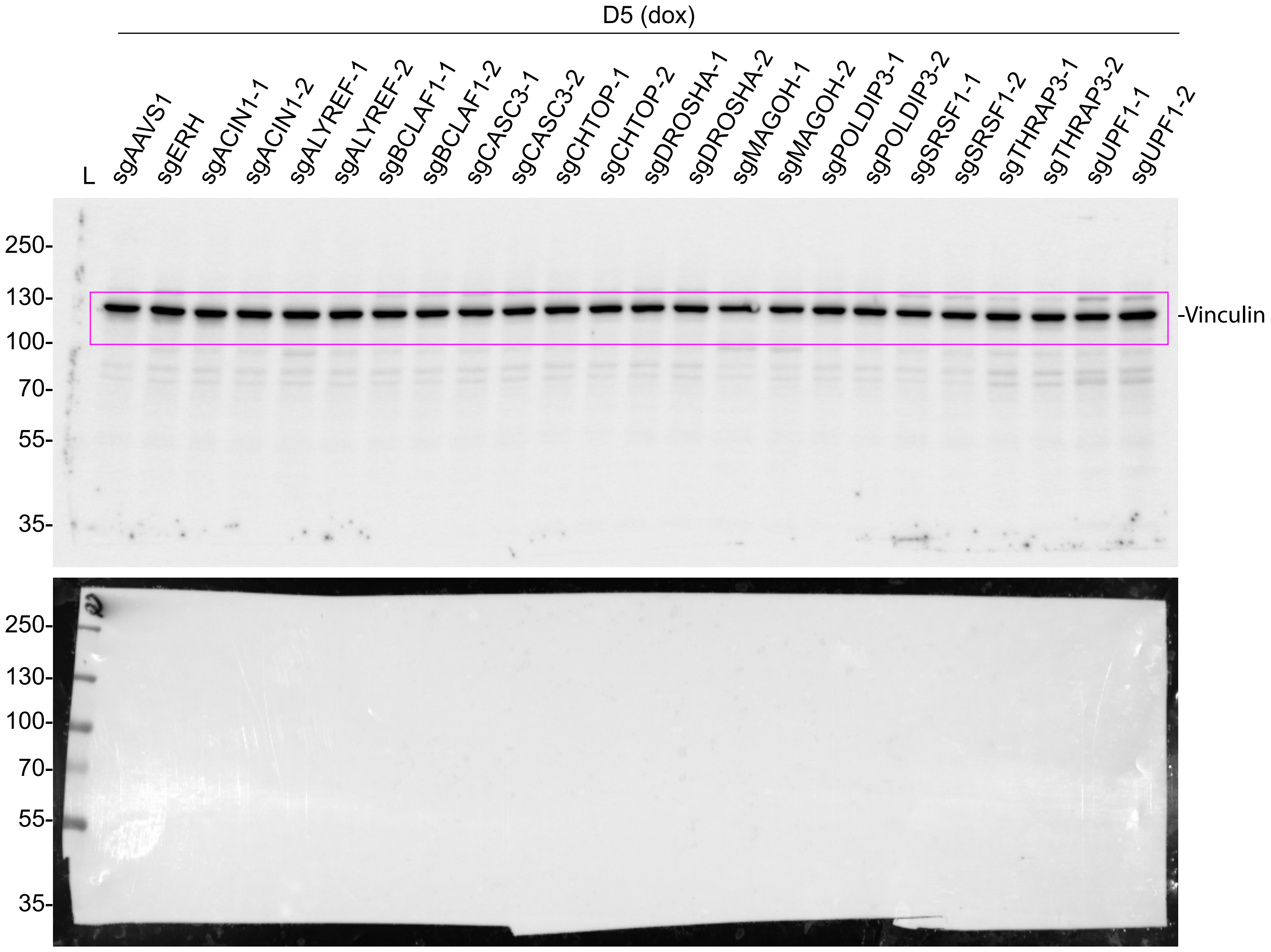

Souce_data_Fig_3E

Replicate 4

RKO, Vinculin (8% Gel 3)

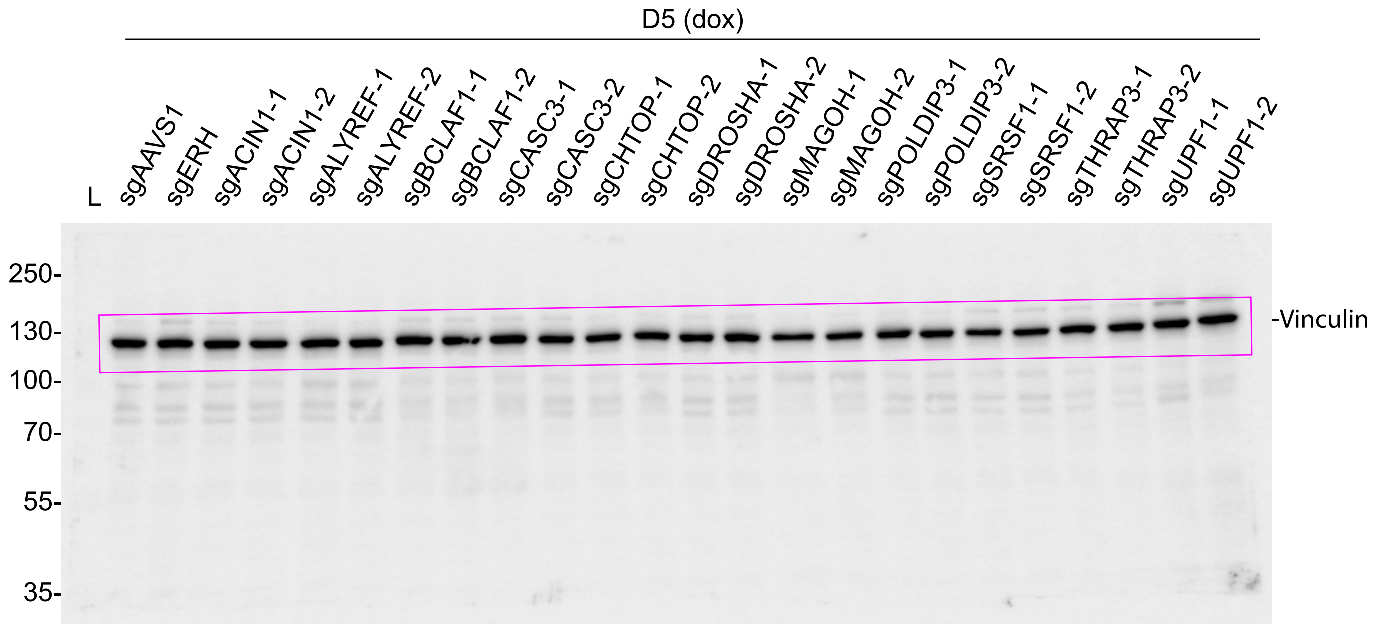

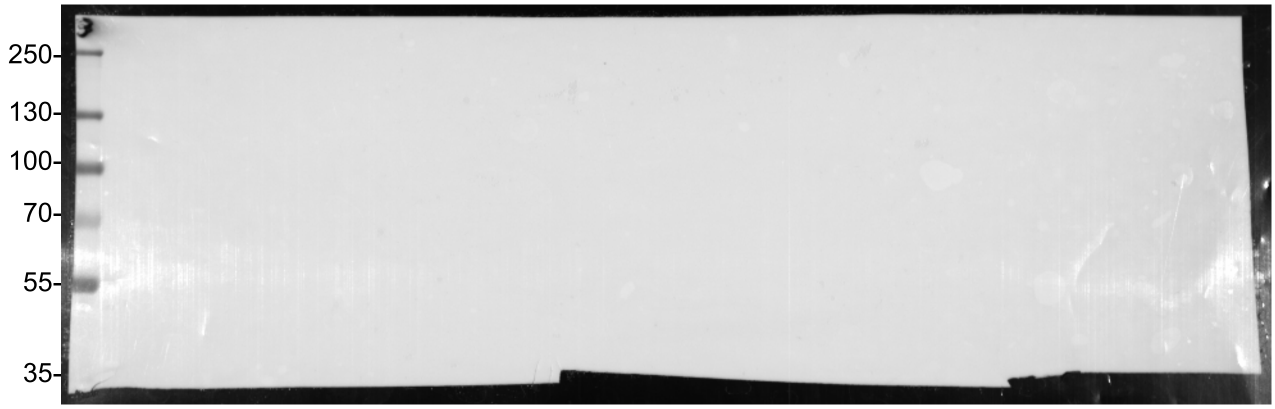

Replicate 1

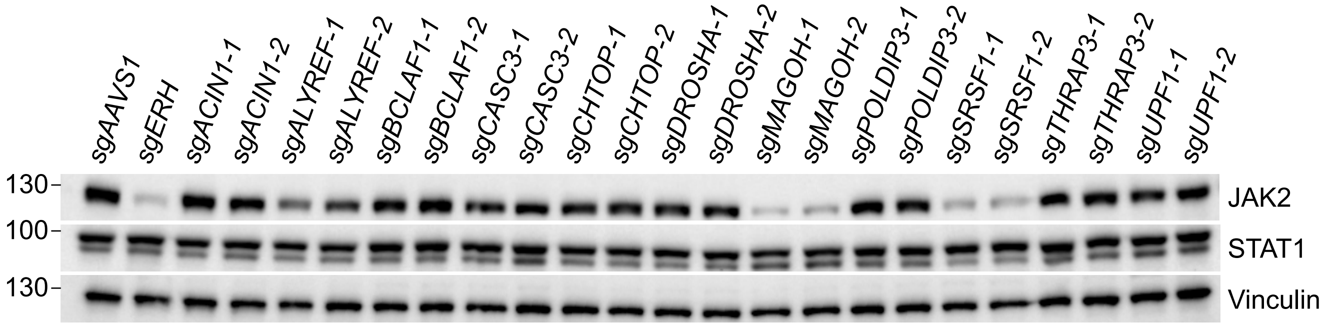

Replicate 2

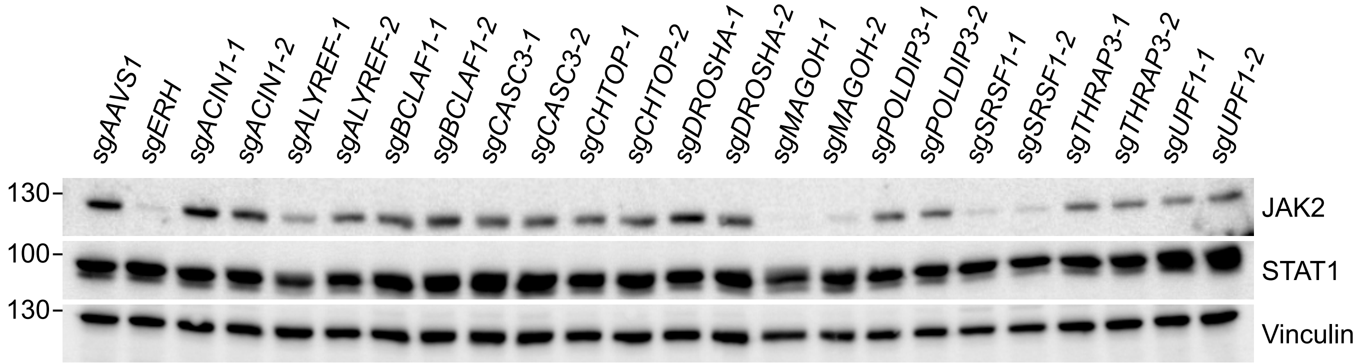

Replicate 3

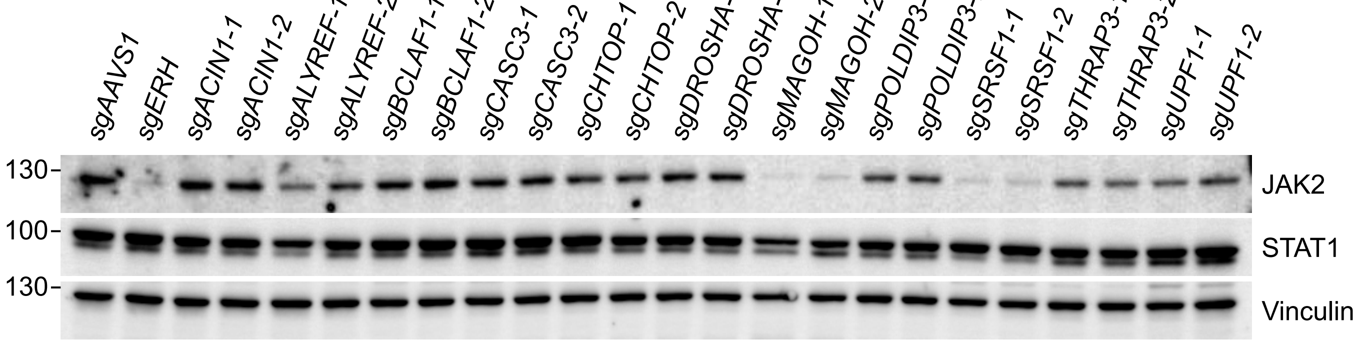

Replicate 4

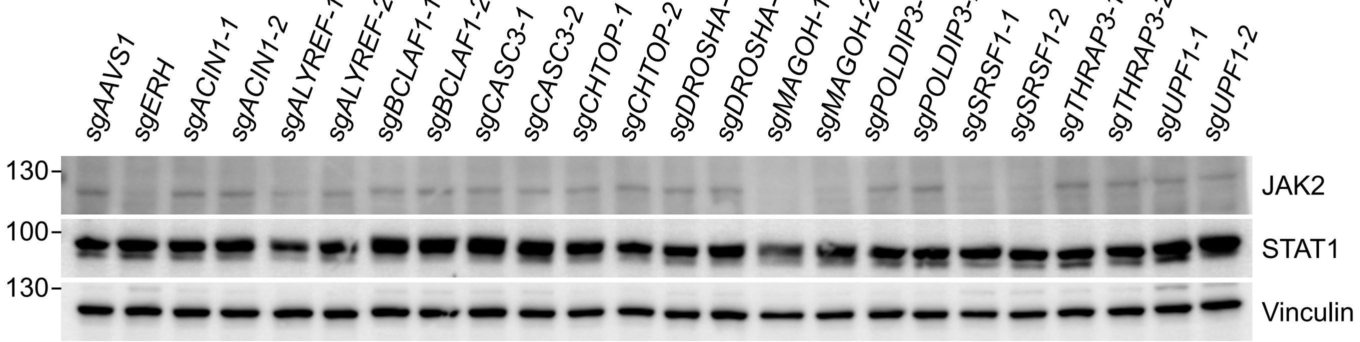

RKO, JAK2 (8% Gel 1)

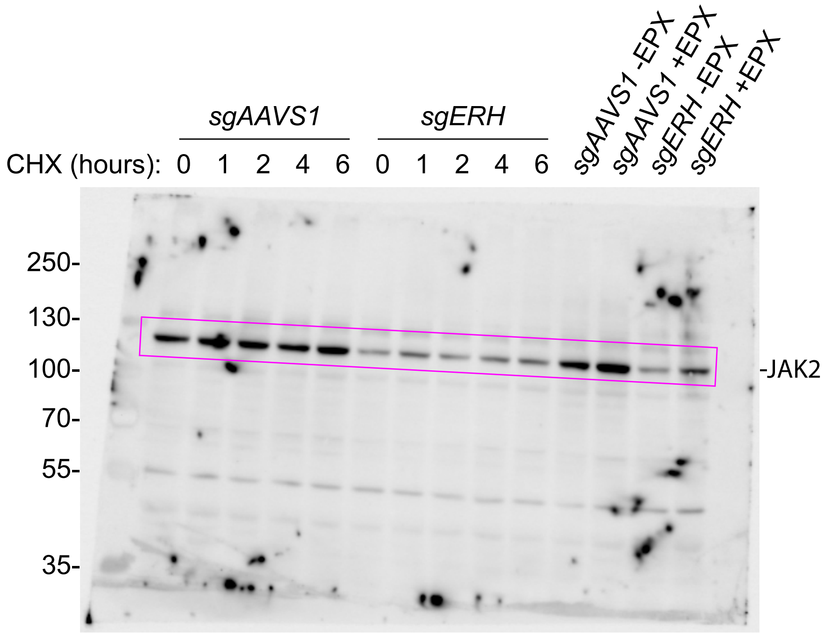

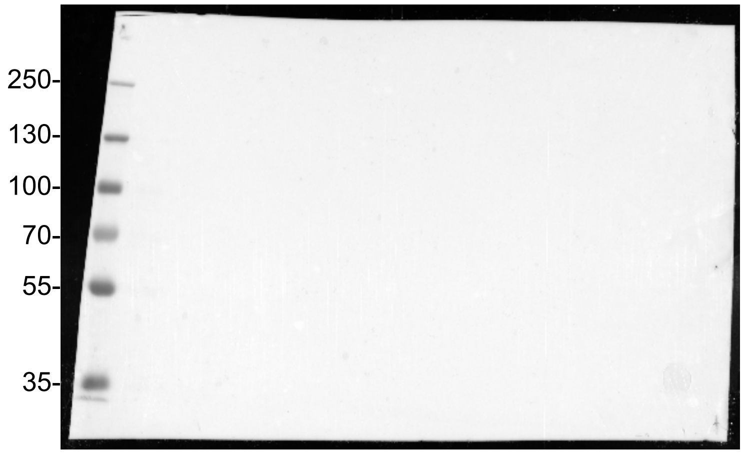

RKO, JAK2 (8% Gel 2)

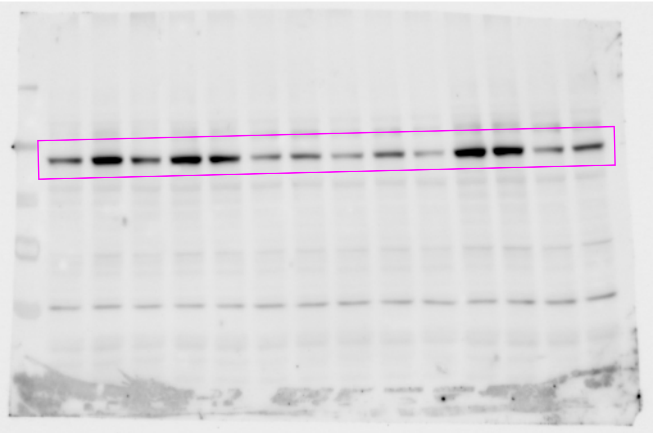

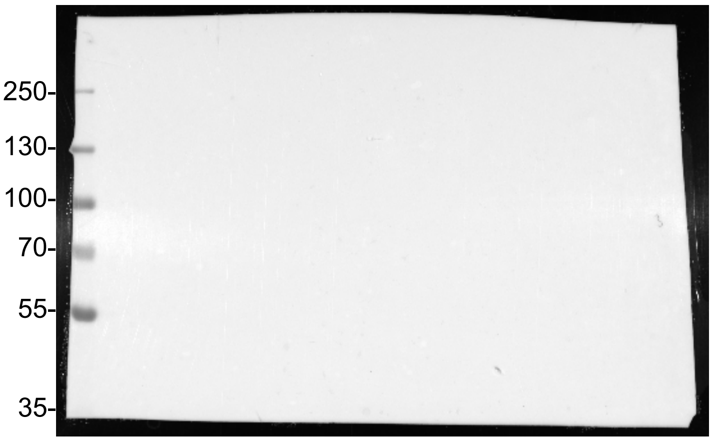

RKO, JAK2 (8% Gel 3)

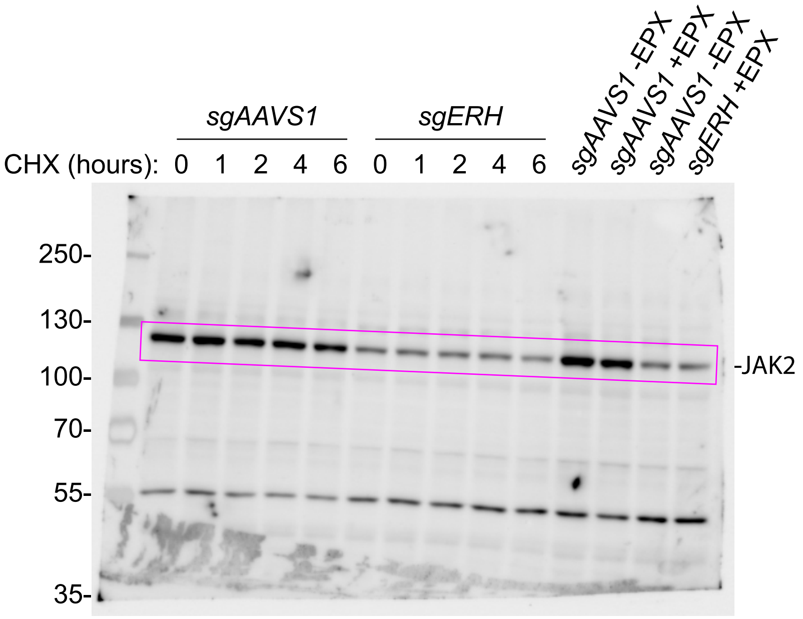

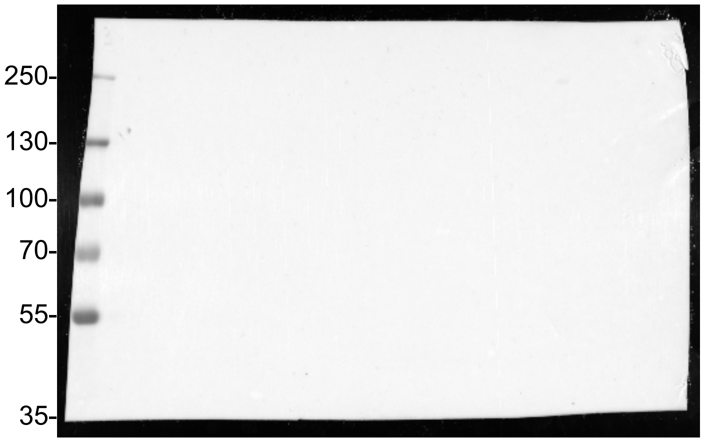

Souce_data_Fig_4D

RKO, MYC (8% Gel 1)

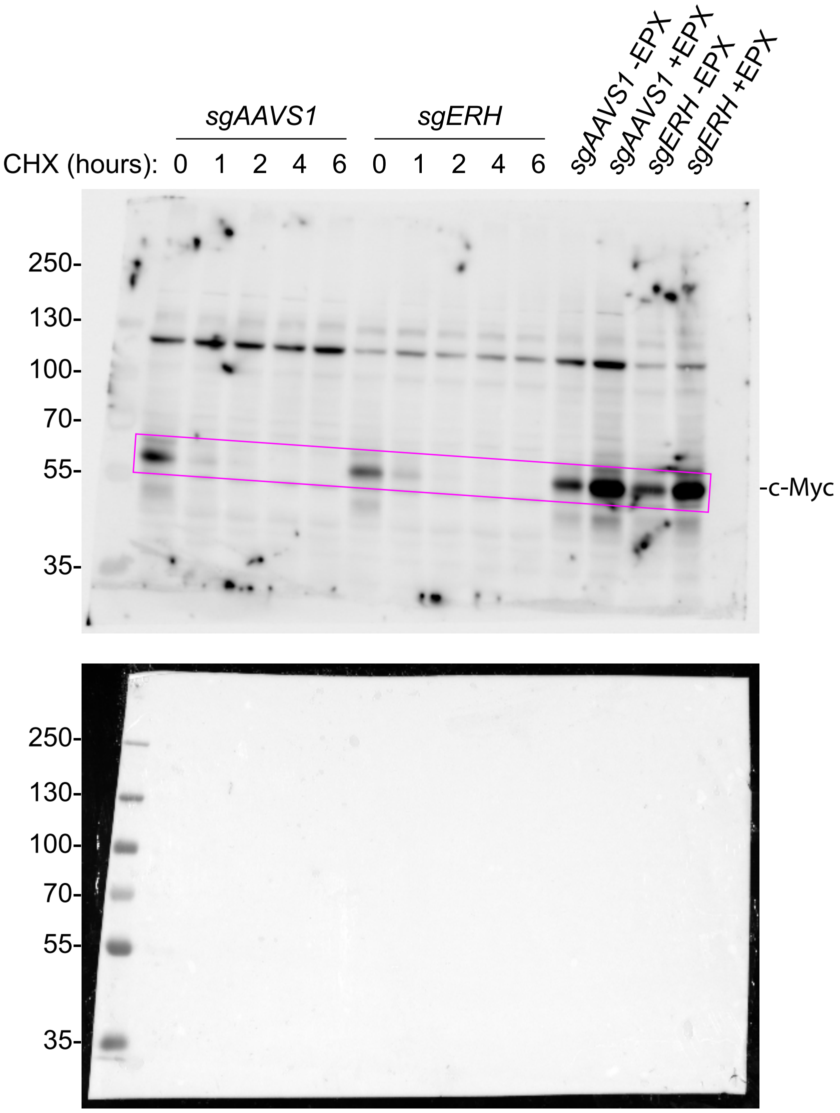

Souce_data_Fig_4D

RKO, MYC (8% Gel 2)

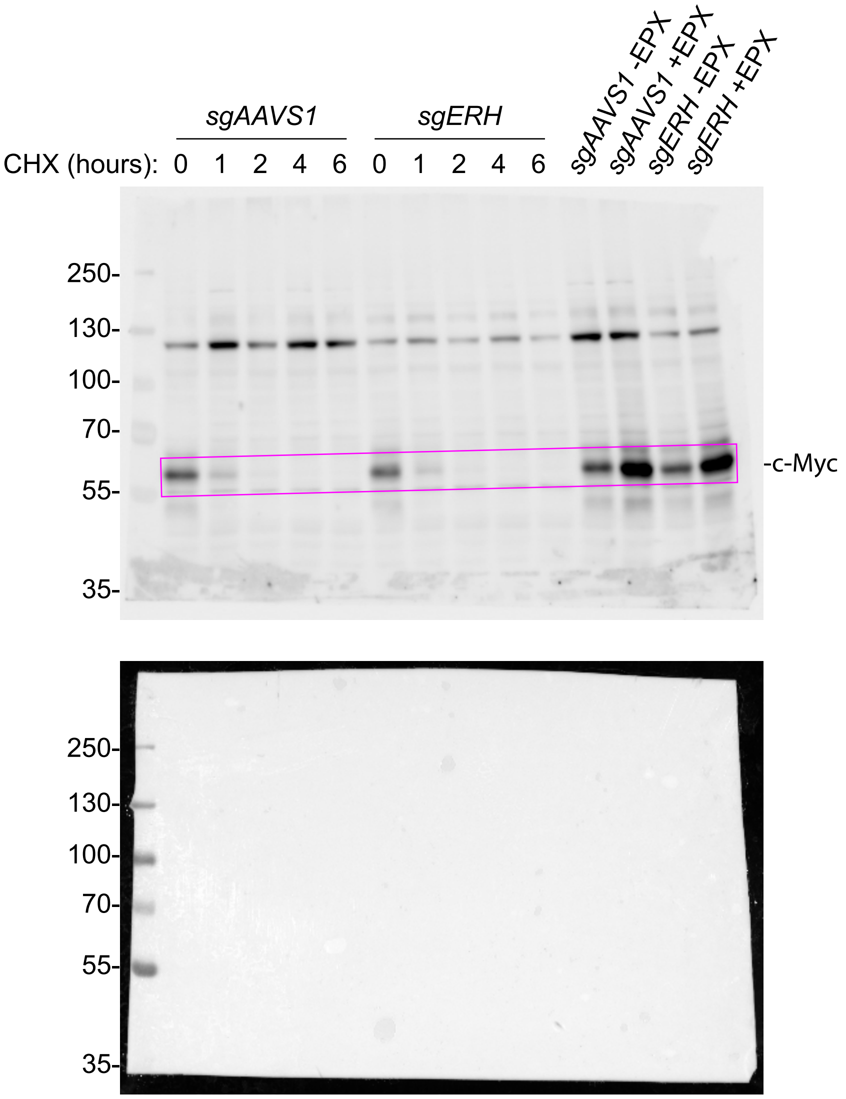

RKO, MYC (8% Gel 3)

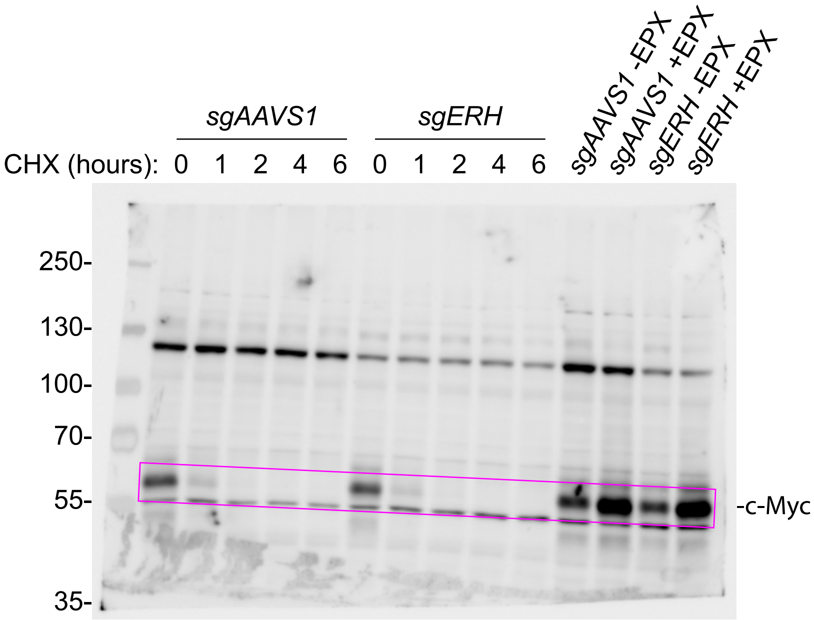

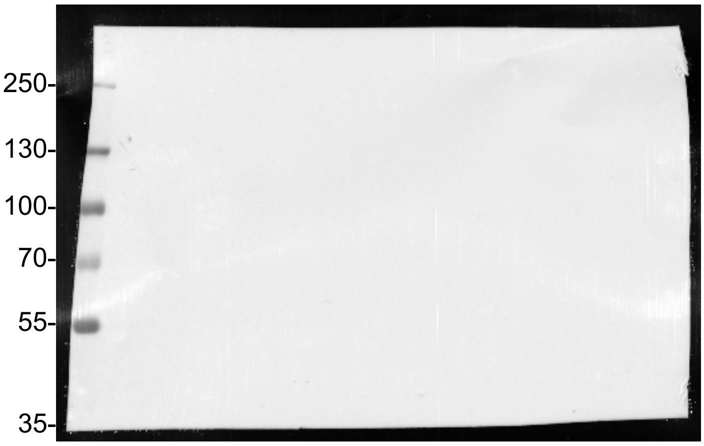

RKO, Vinculin (8% Gel 1)

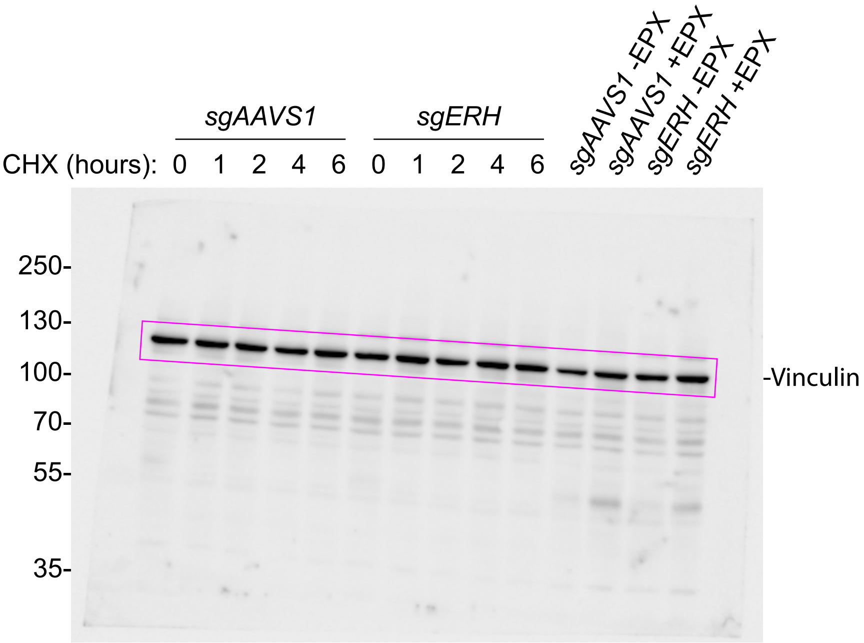

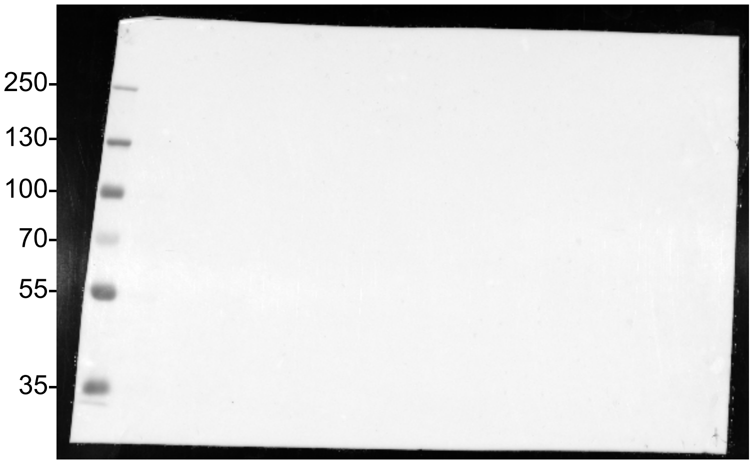

RKO, Vinculin (8% Gel 2)

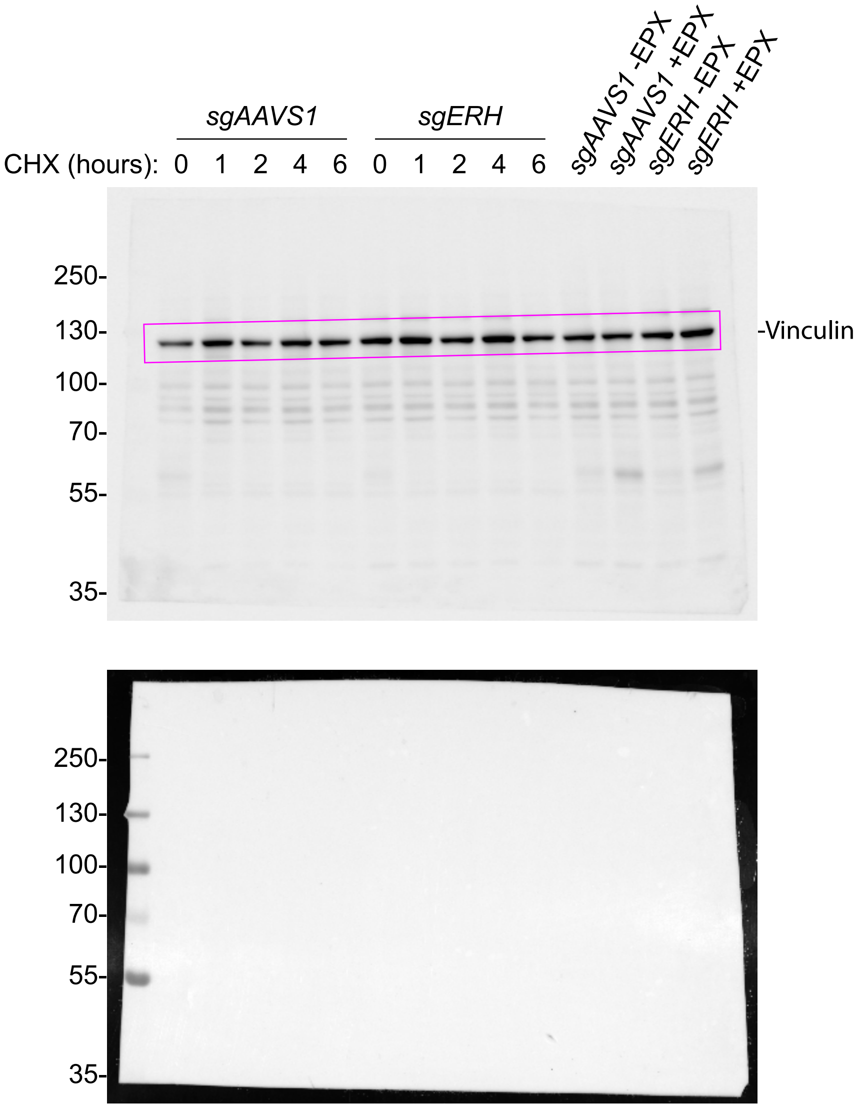

Souce_data_Fig_4D

RKO, Vinculin (8% Gel 3)

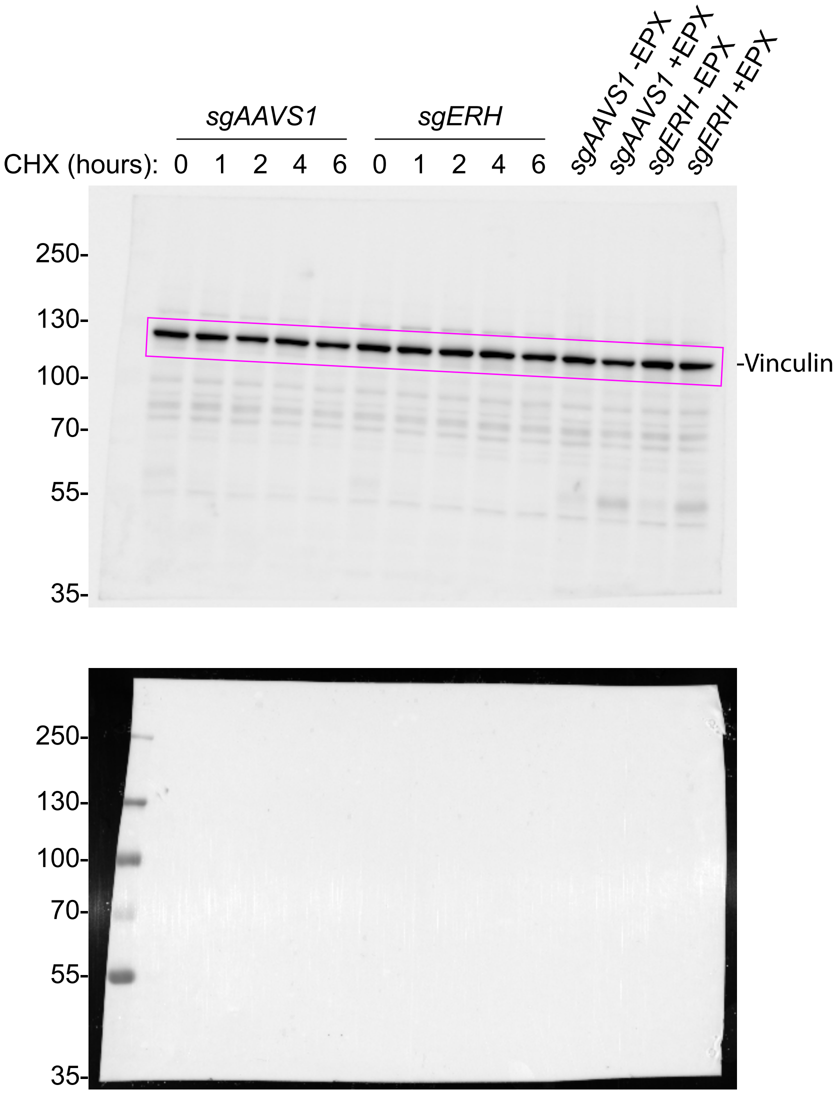

RKO, Tubulin (8% Gel 1)

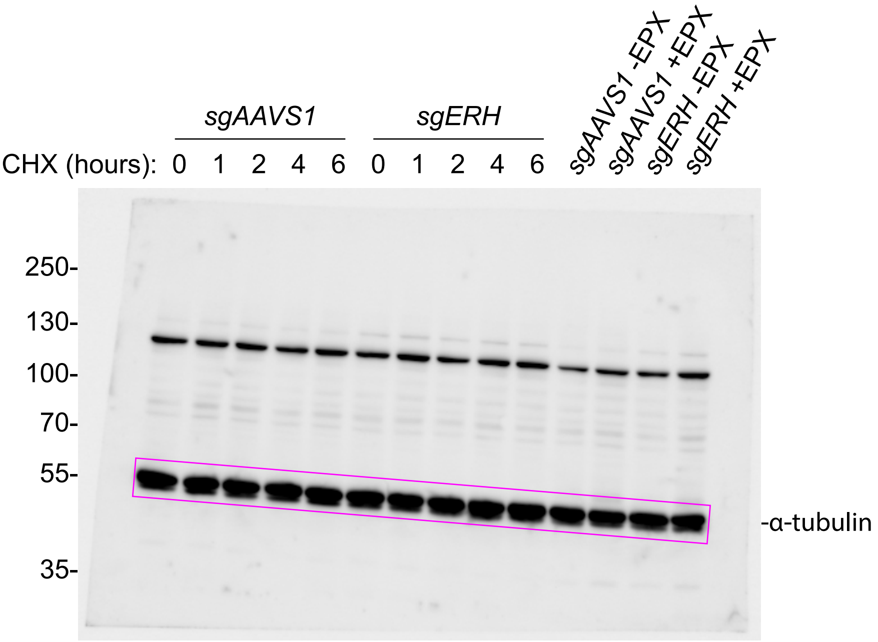

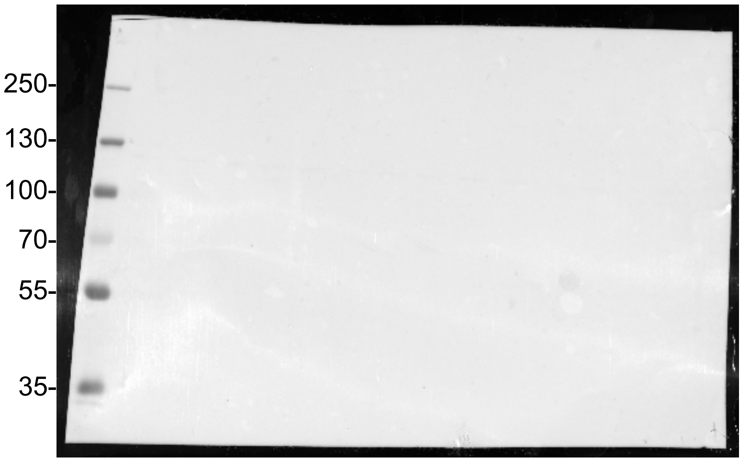

RKO, Tubulin (8% Gel 2)

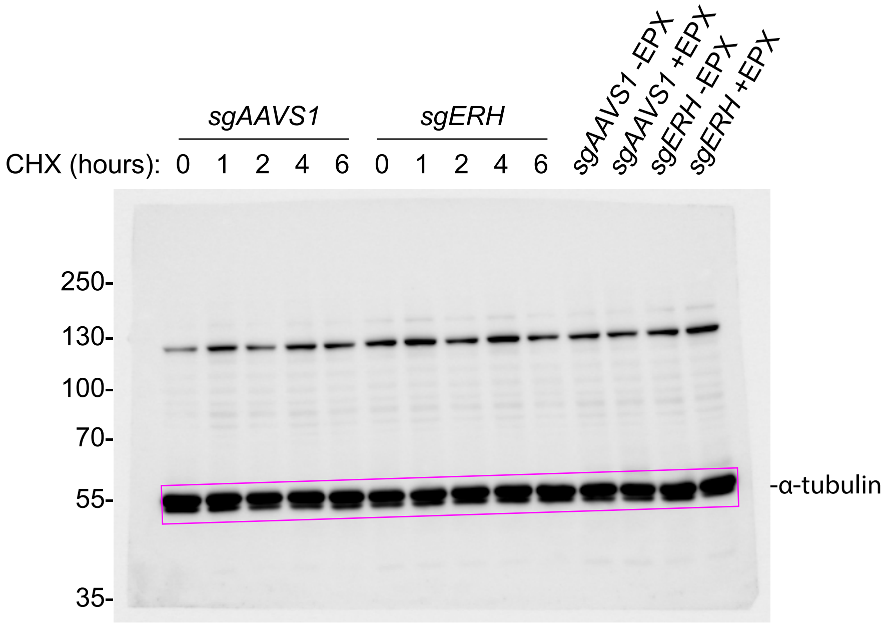

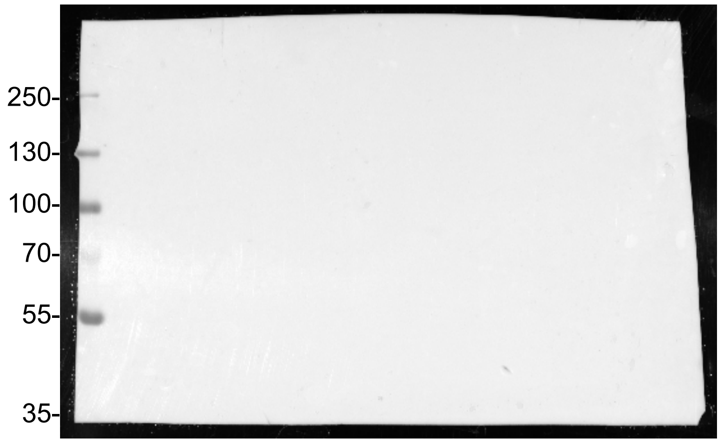

RKO, Tubulin (8% Gel 3)

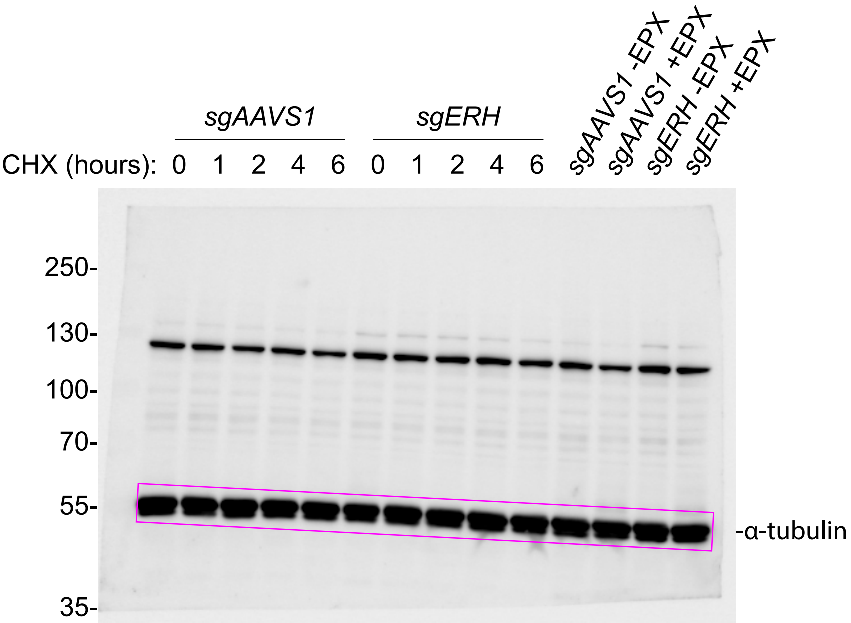

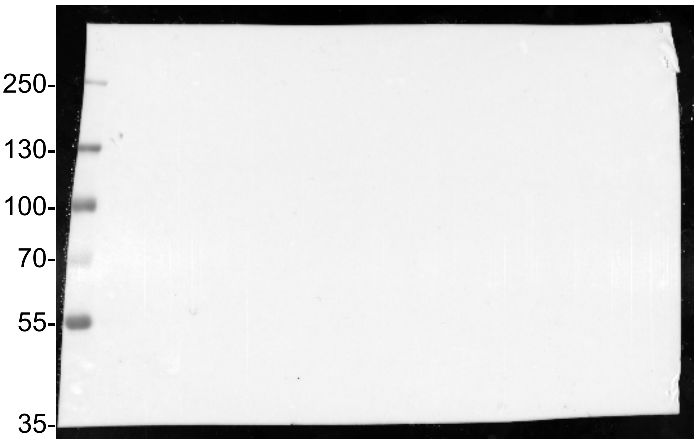

Souce_data_Fig_4D

Replicate 1

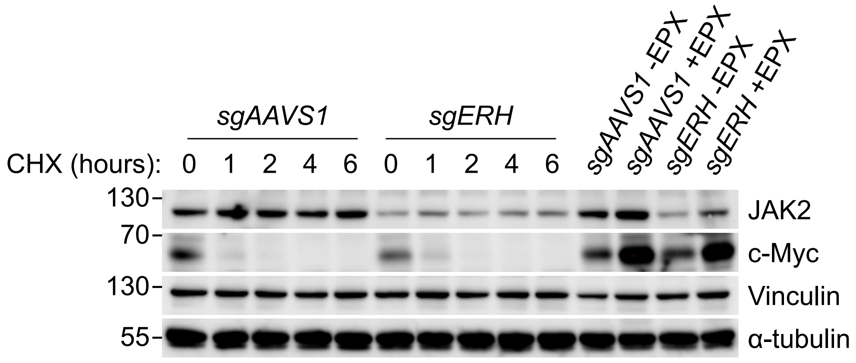

Replicate 2

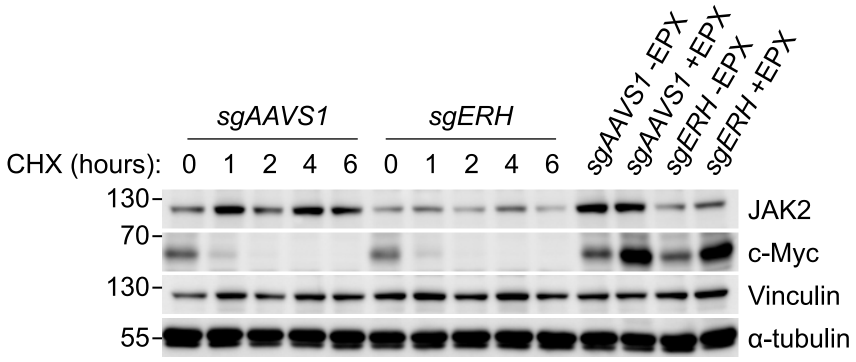

Replicate 3

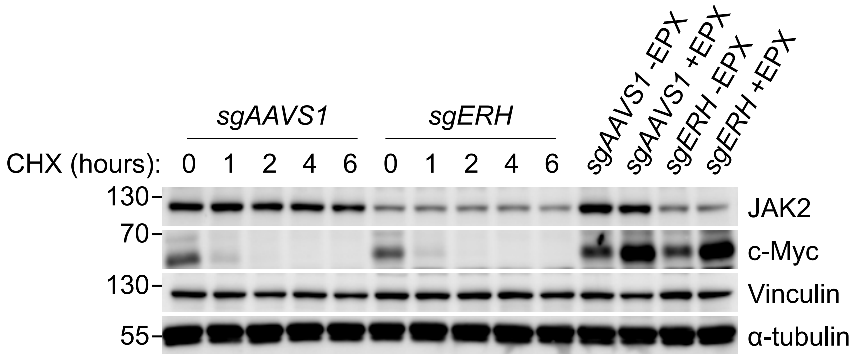

Souce_data_Fig_S2E

RAW 264.7, JAK2 (8% Gel 1 and Gel 2)

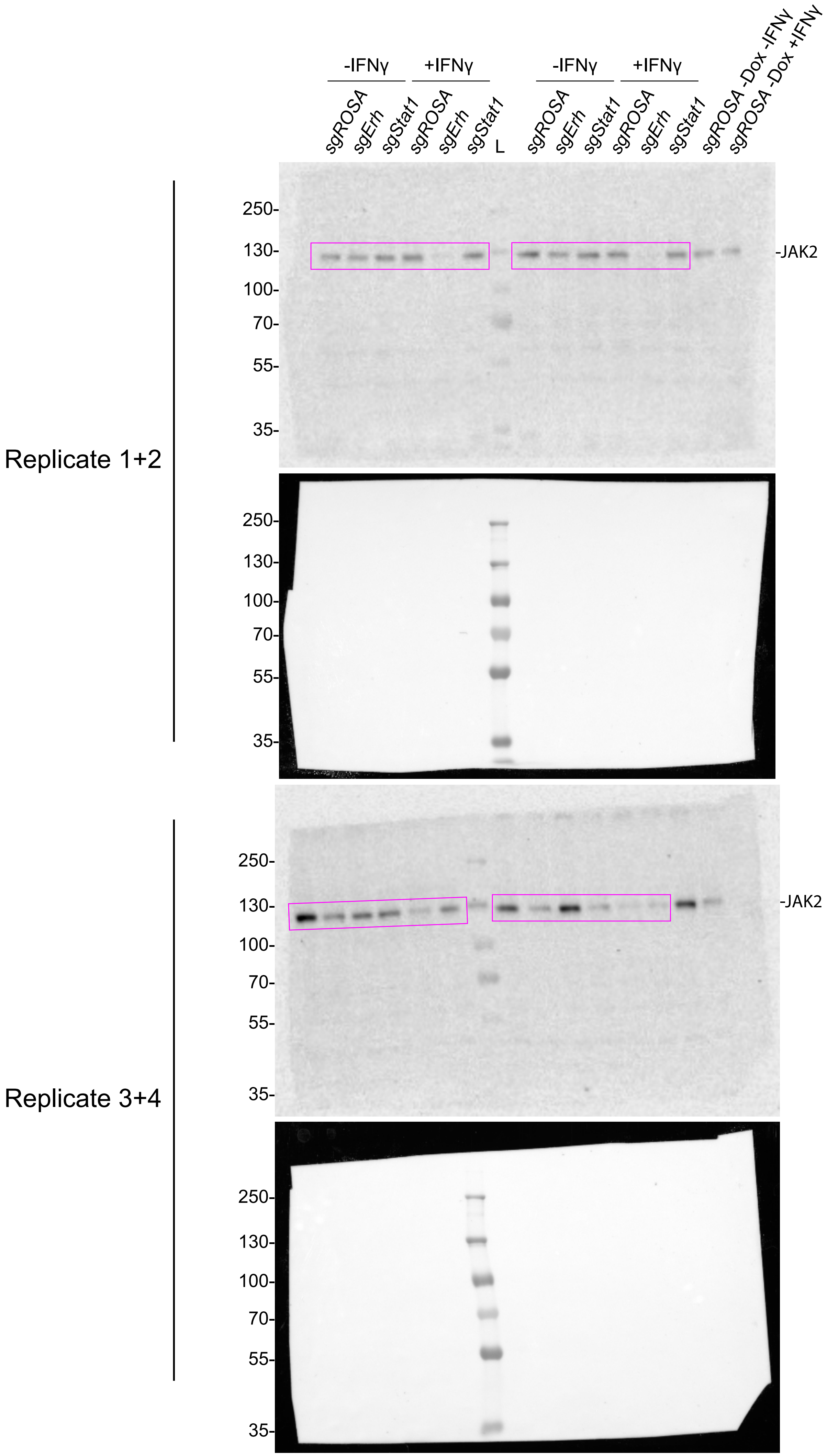

Souce_data_Fig_S2E

RAW 264.7, IRF1 (8% Gel 3 and Gel 4)

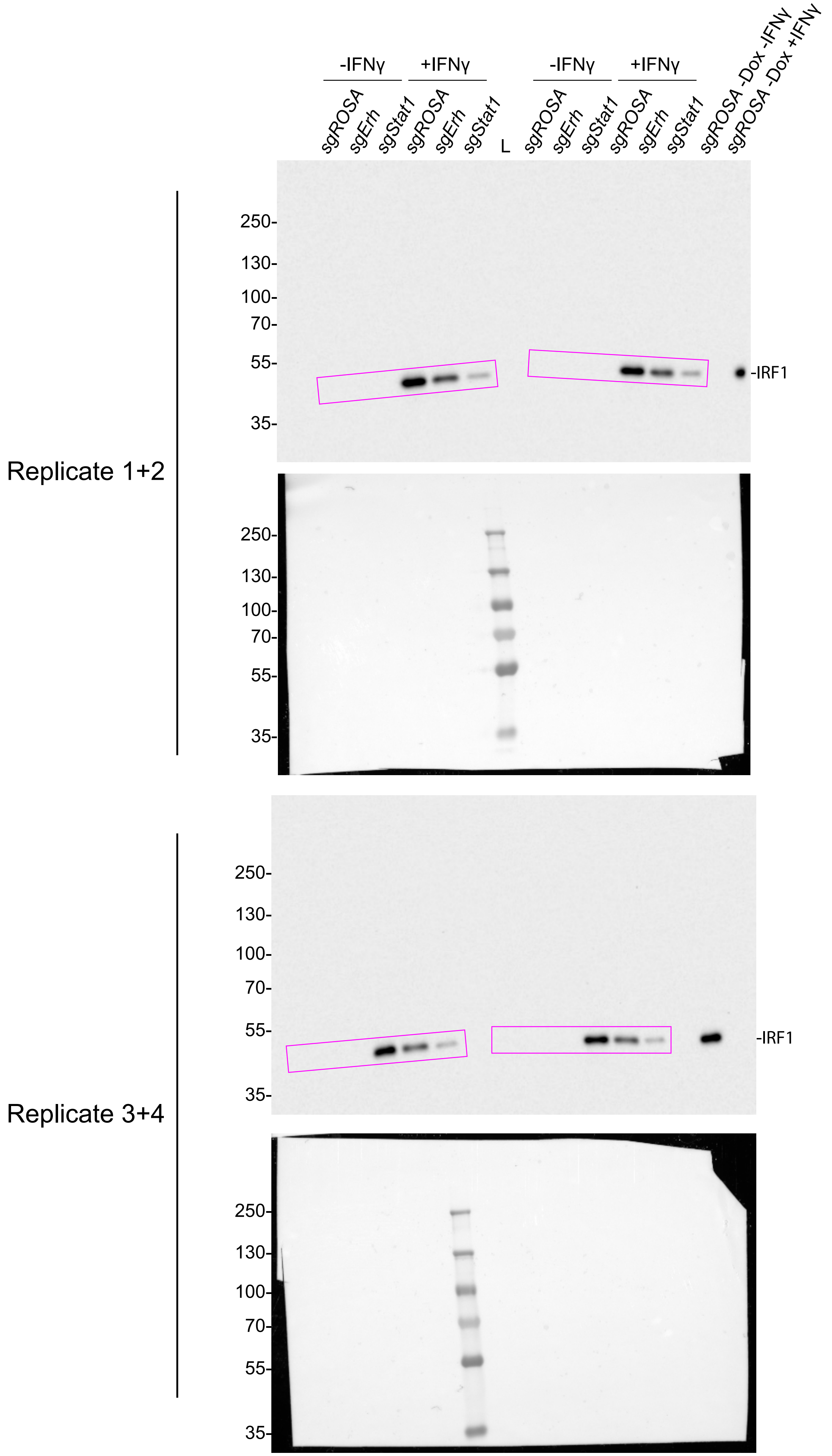

Souce_data_Fig_S2E

RAW 264.7, ERH (20% Gel)

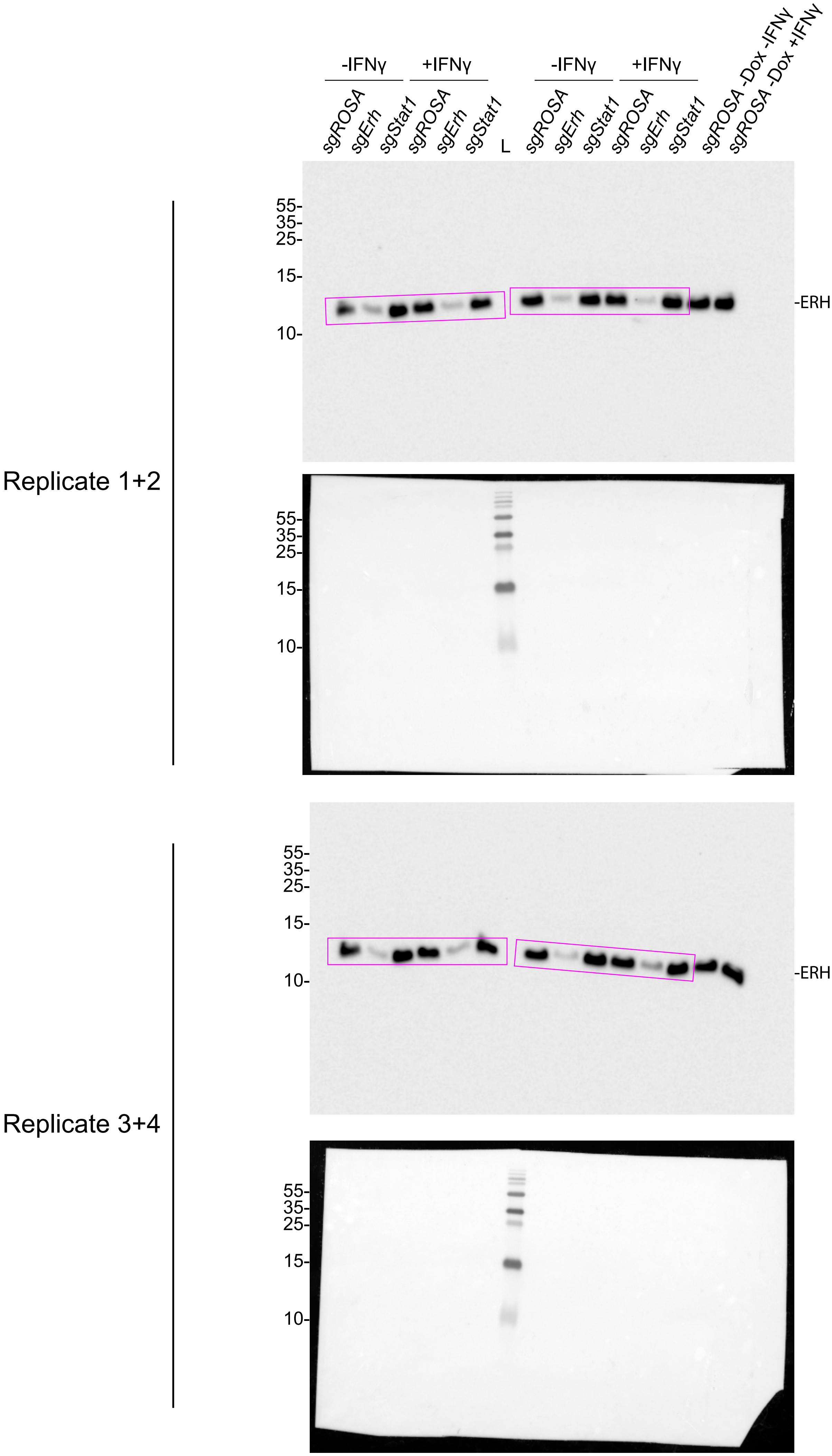

Souce_data_Fig_S2E

RAW 264.7, p-STAT1 (8% Gel 1 and Gel 2)

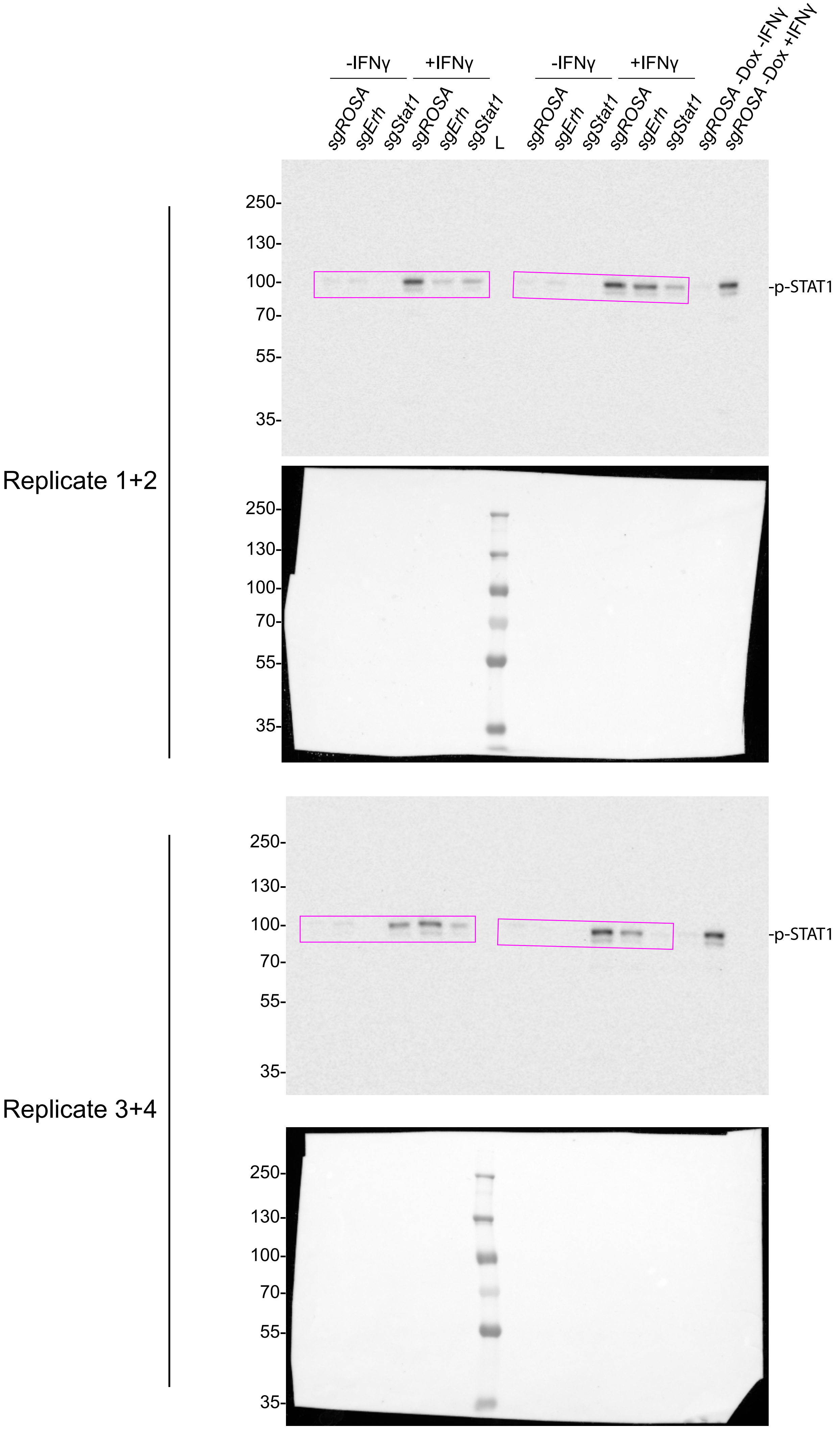

Souce_data_Fig_S2E

RAW 264.7, JAK1 (8% Gel 3 and Gel 4)

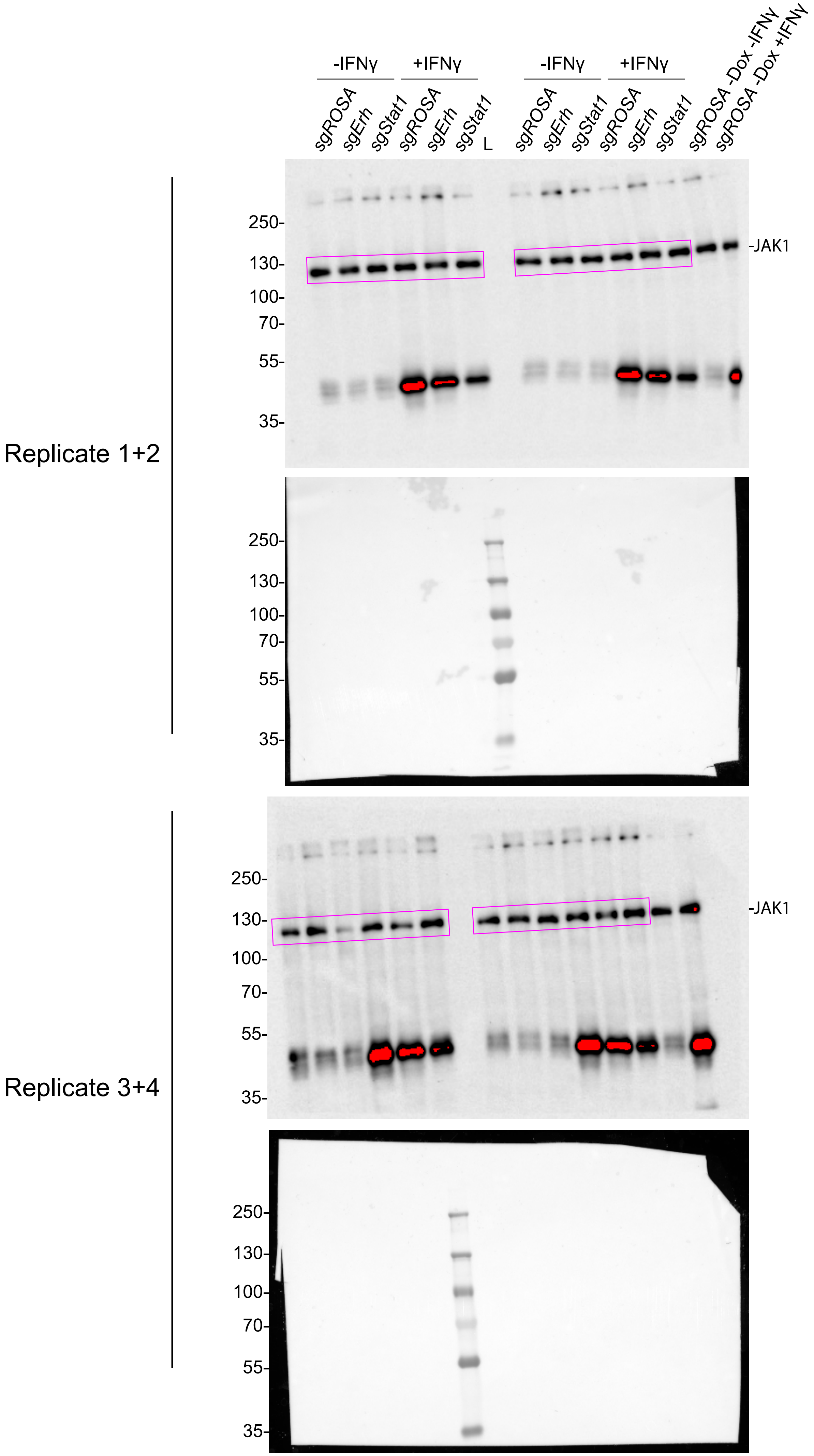

Souce_data_Fig_S2E

RAW 264.7, STAT1 (8% Gel 1 and Gel 2)

Replicate 1+2

Replicate 3+4

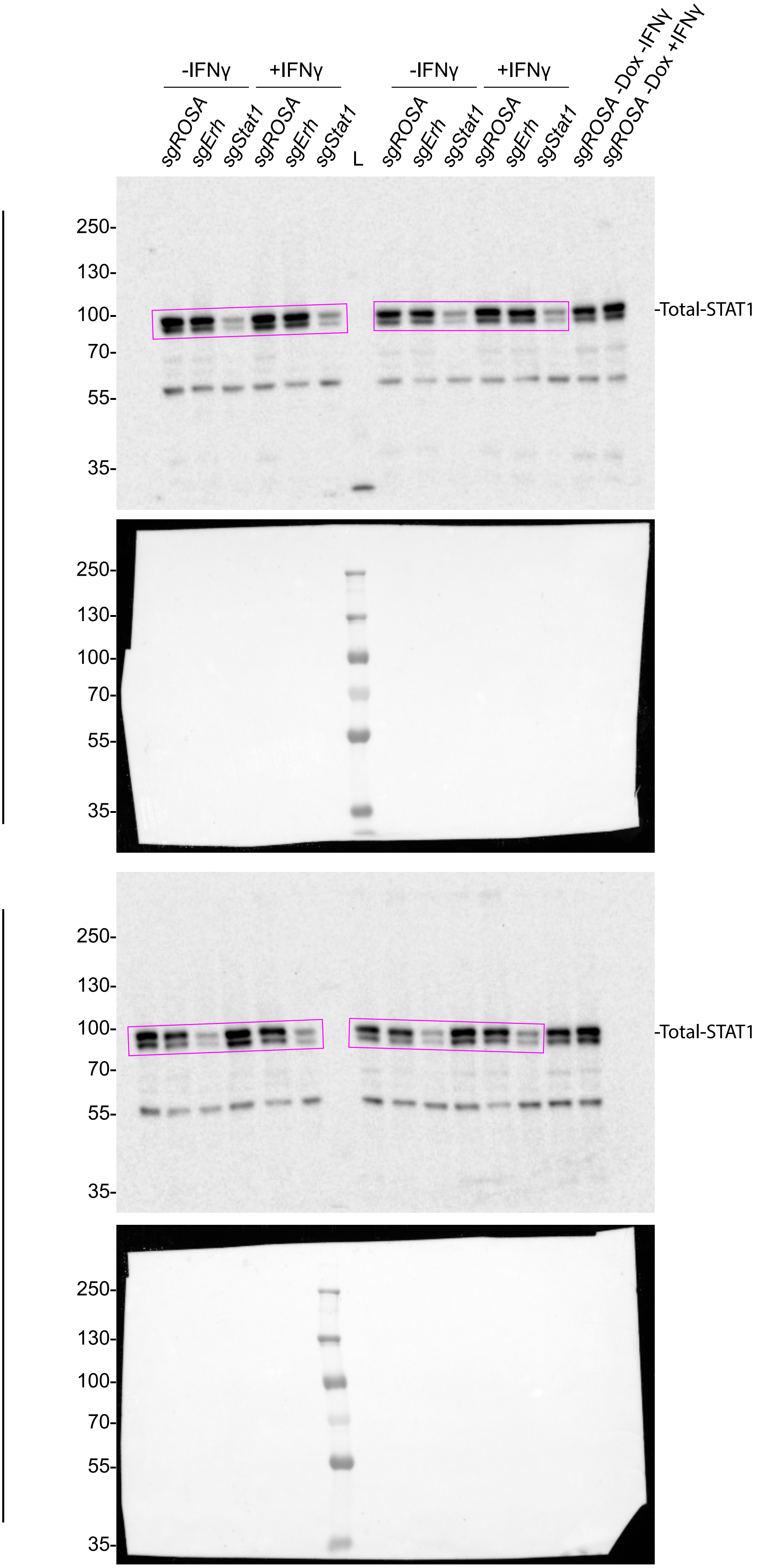

Souce_data_Fig_S2E

RAW264.7, β-Actin (8% Gel 2 and Gel 4)

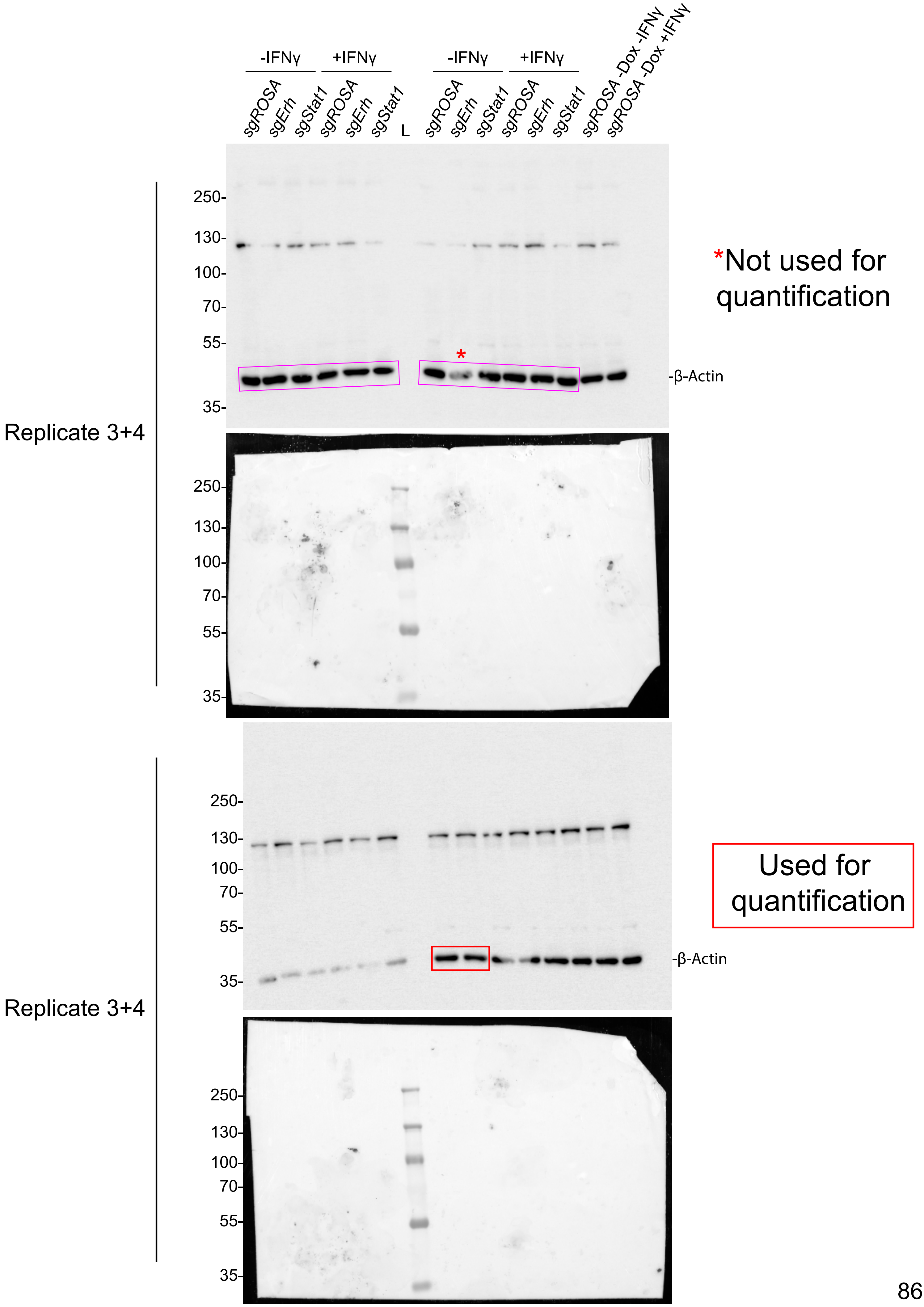

Souce_data_Fig_S2E

RAW264.7, β-Actin (8% Gel 3 and Gel 4)

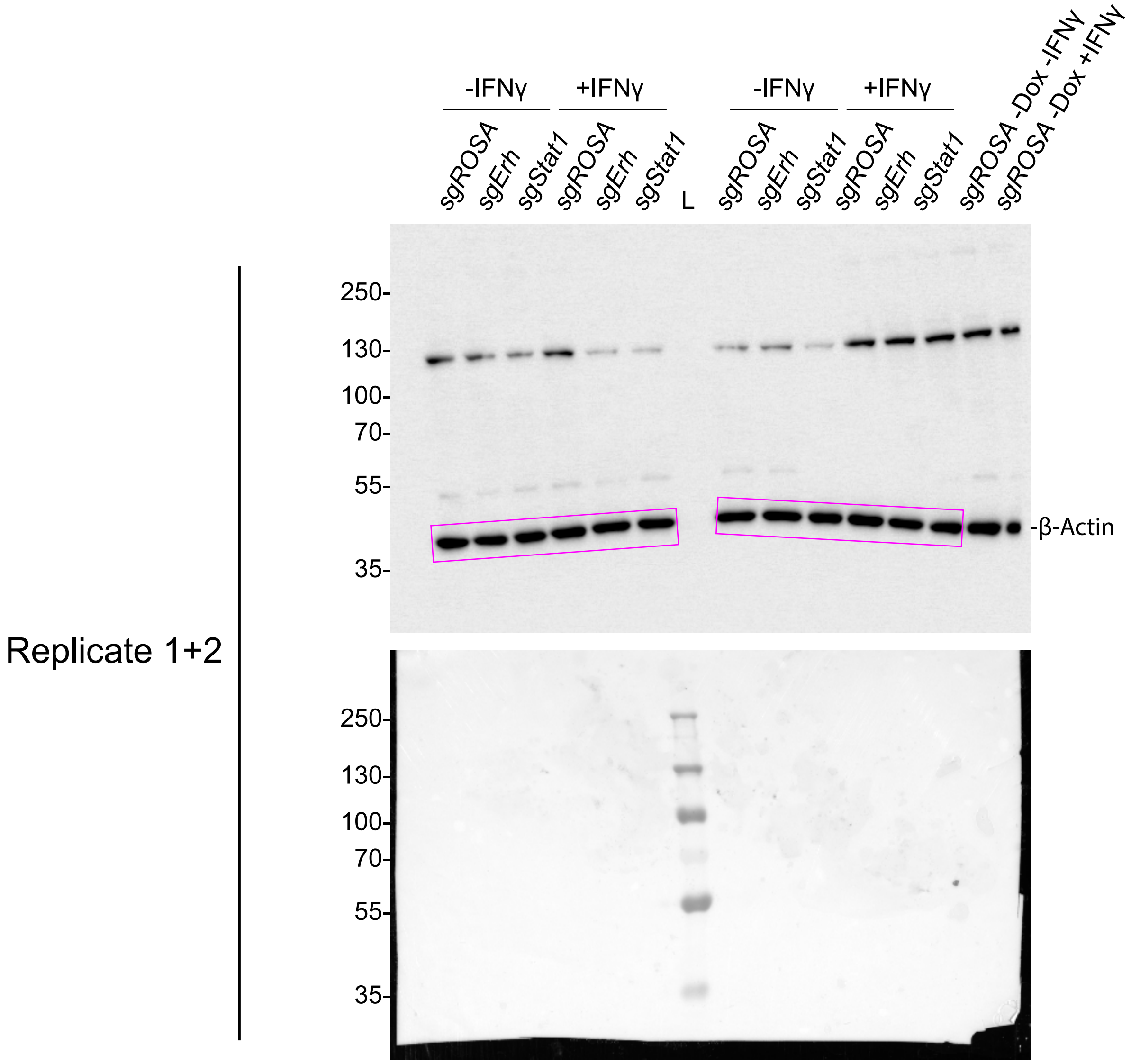

Replicate 5

RAW 264.7, β-Actin (20%)

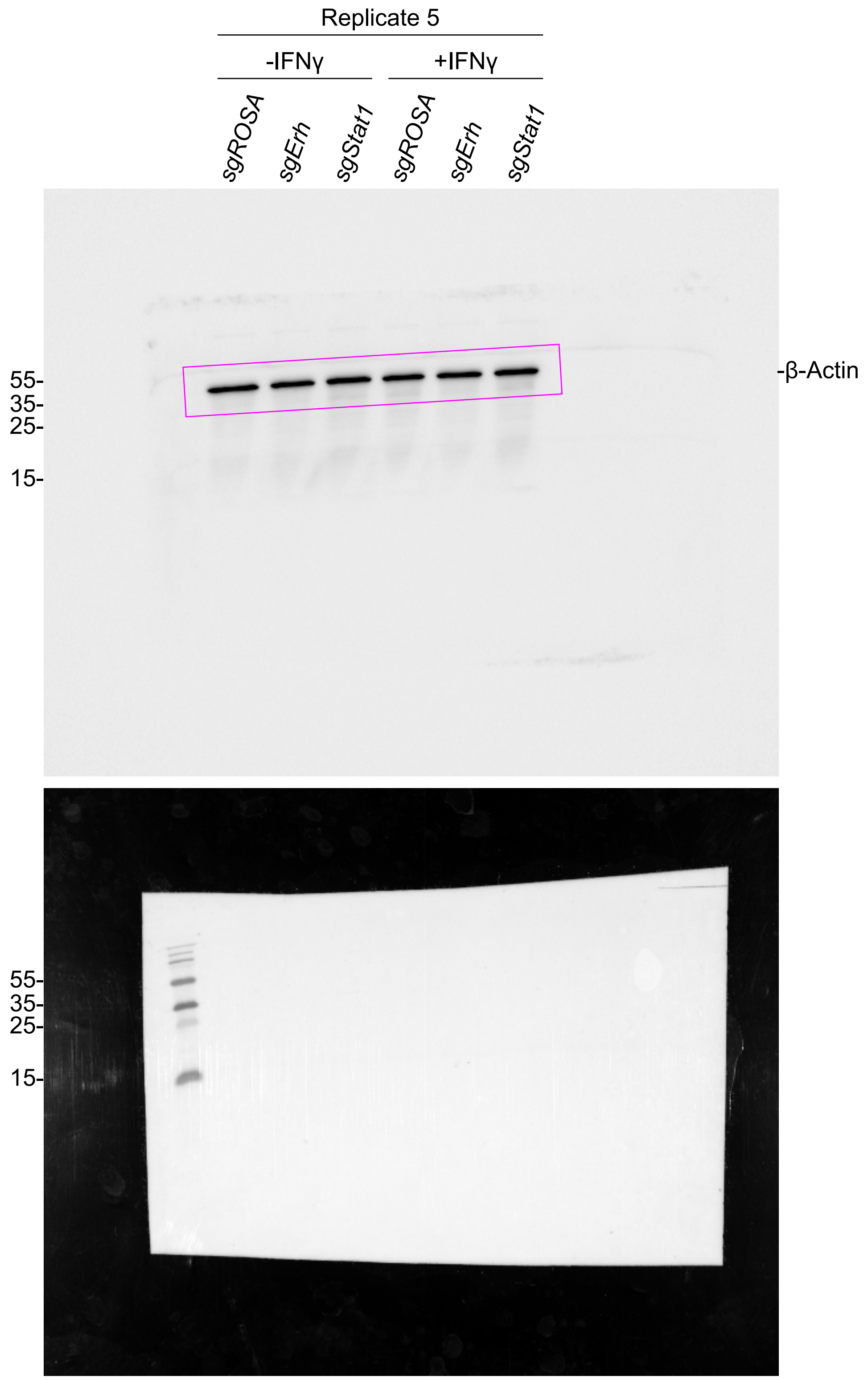

Replicate 5

RAW 264.7, ERH (20%)

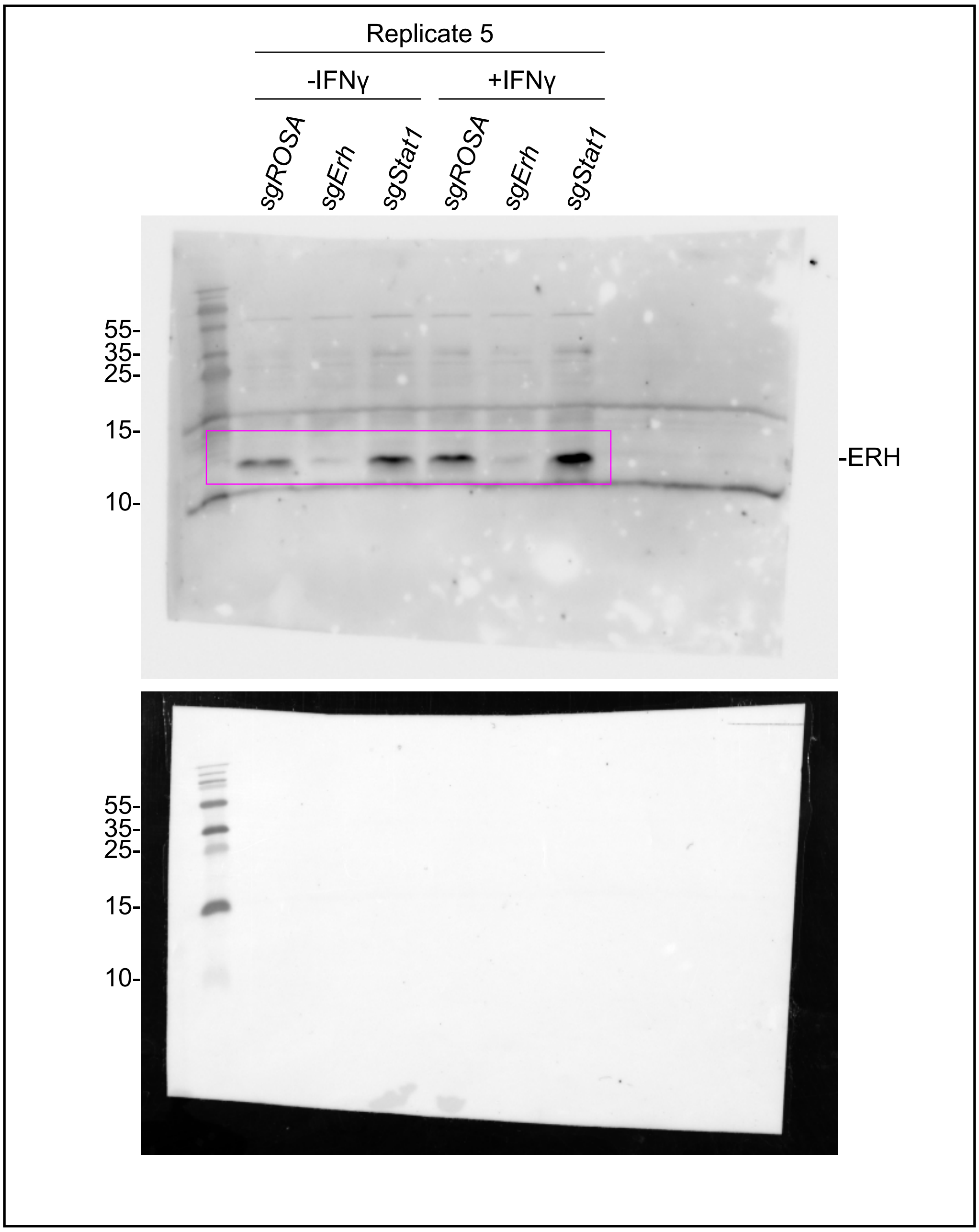

Replicate 5

RAW 264.7, β-Actin (10%)

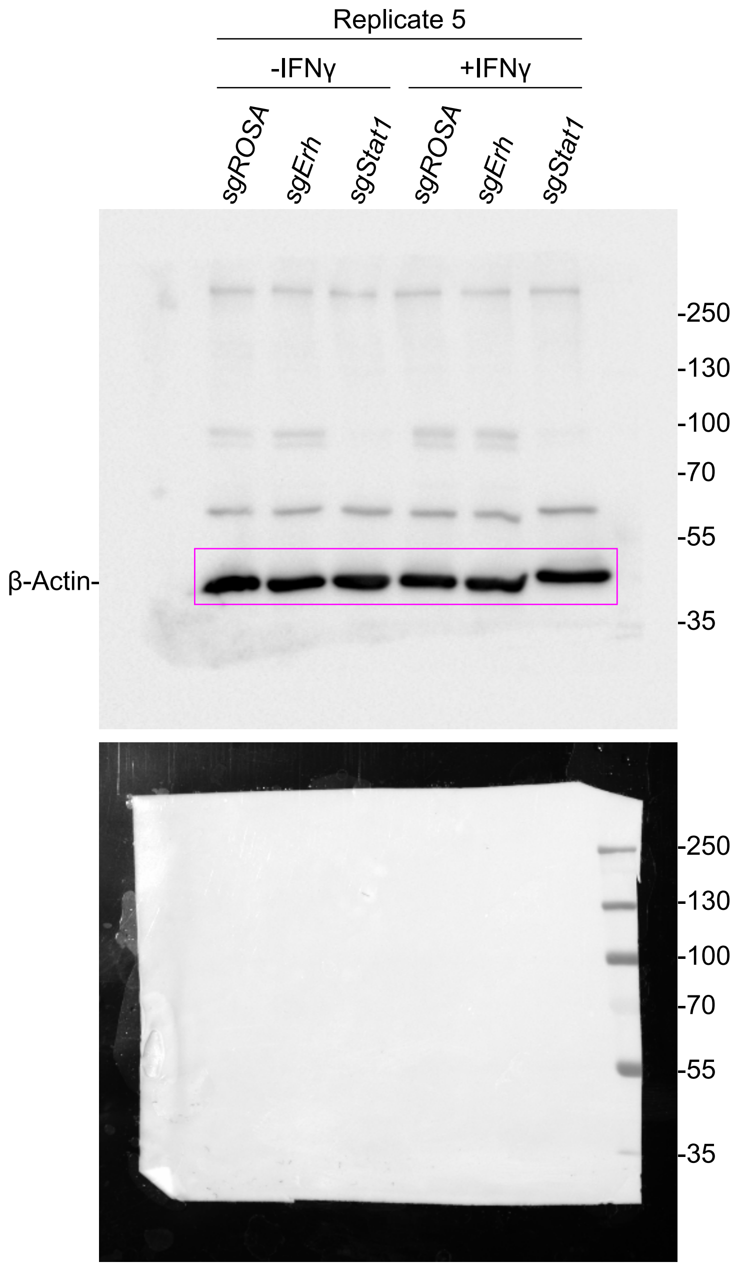

Replicate 5

RAW 264.7, IRF1 (10%)

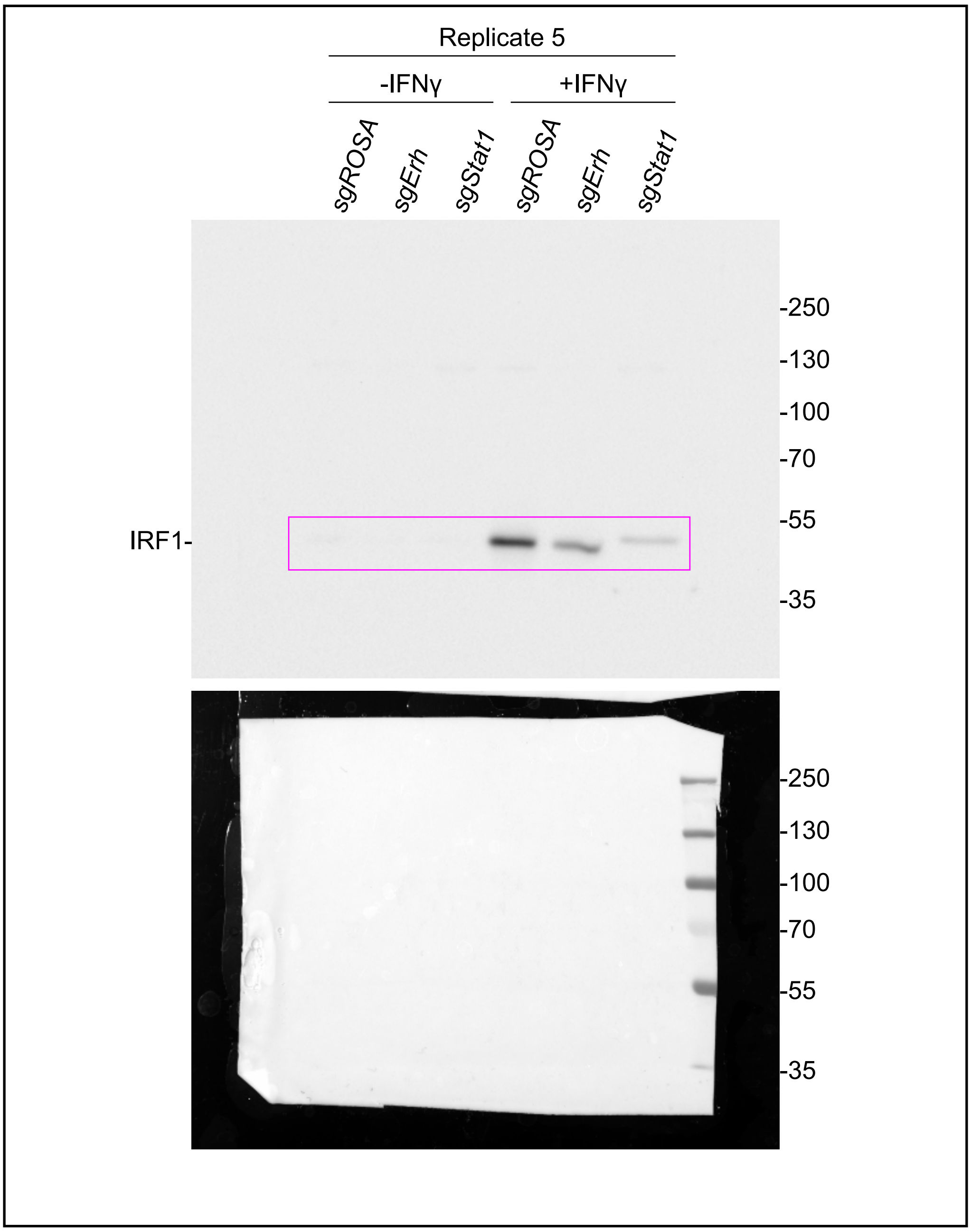

Replicate 5

RAW 264.7, JAK2 (10%)

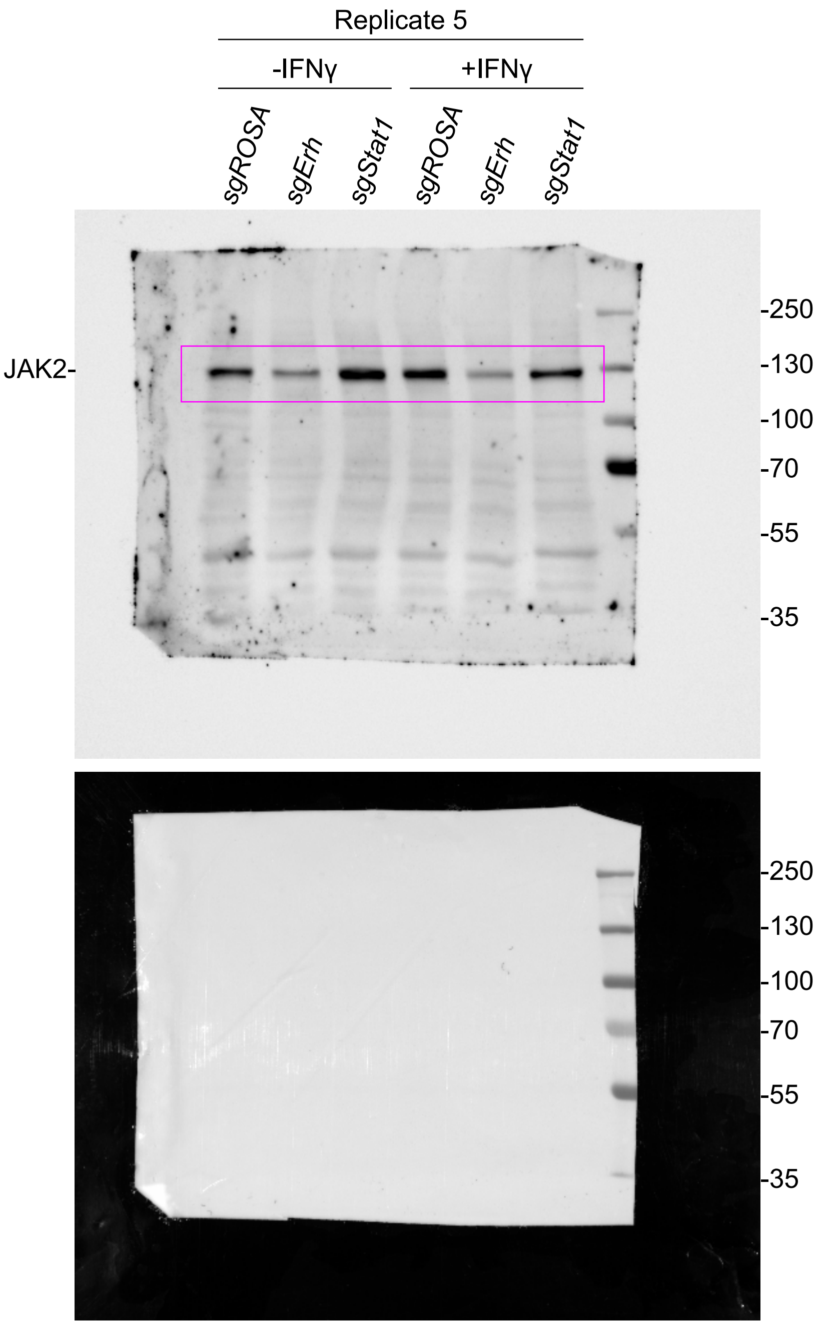

Replicate 5

RAW 264.7, p-STAT1 (10%)

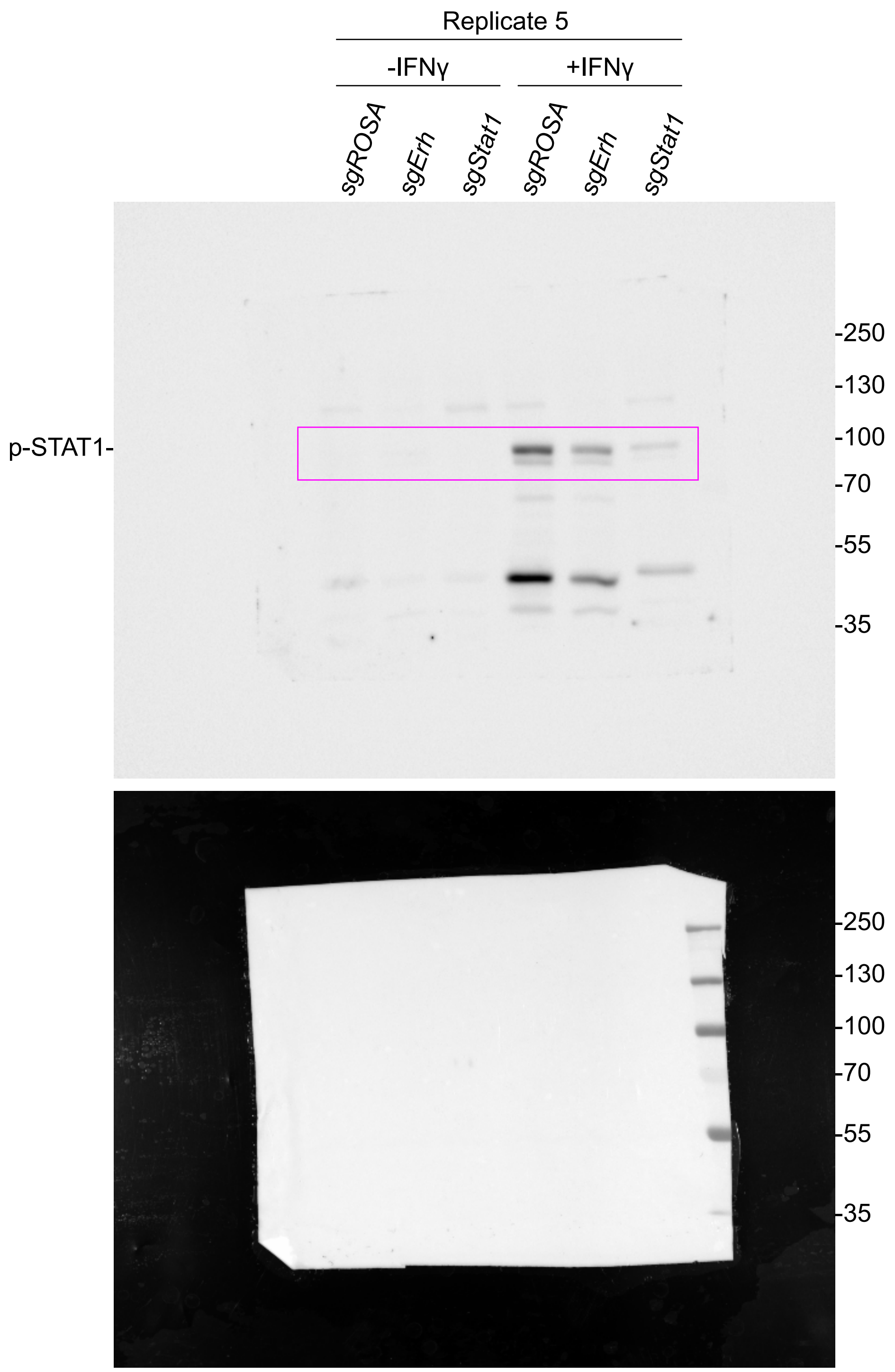

Replicate 5

RAW 264.7, β-Actin (8%)

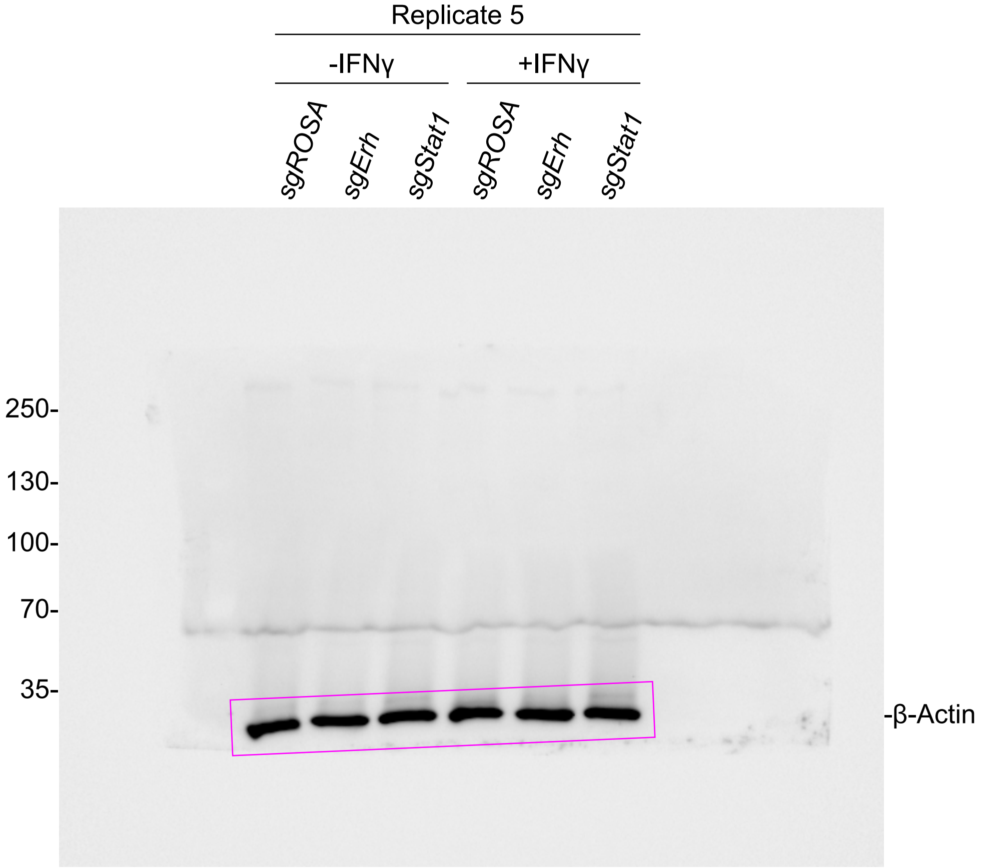

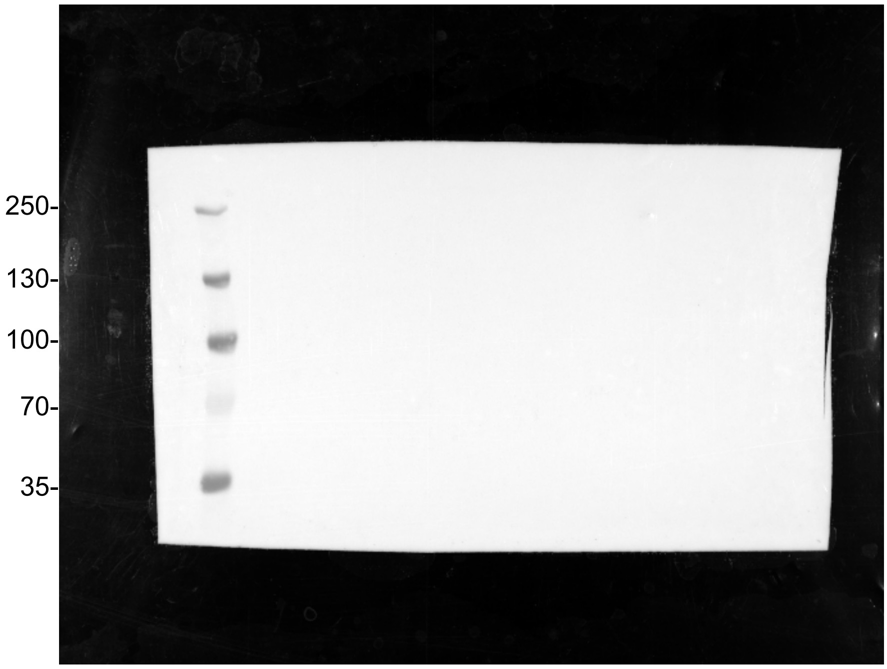

Replicate 5

RAW 264.7, JAK1 (8%)

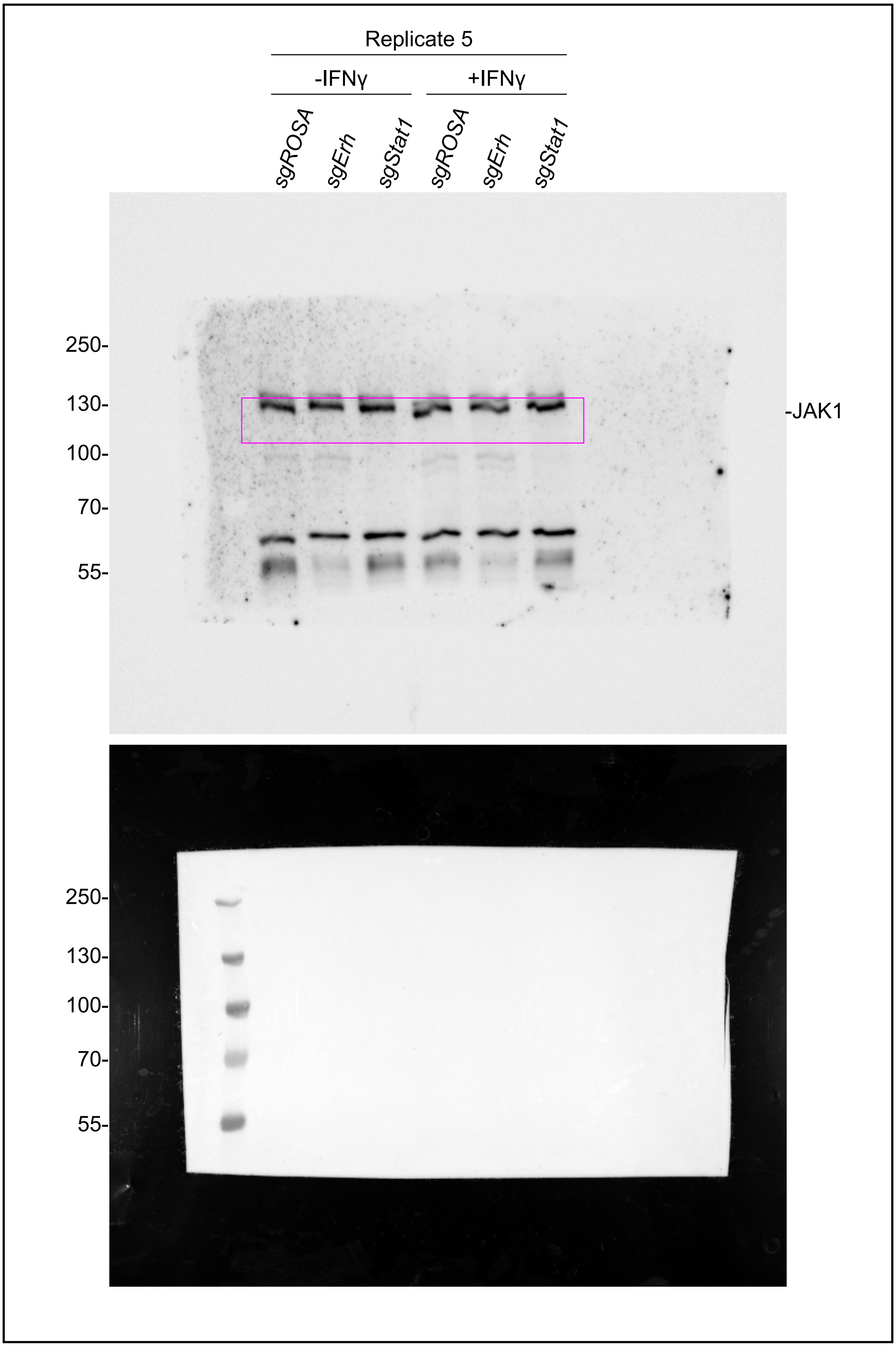

Replicate 5

RAW 264.7, STAT1 (8%)

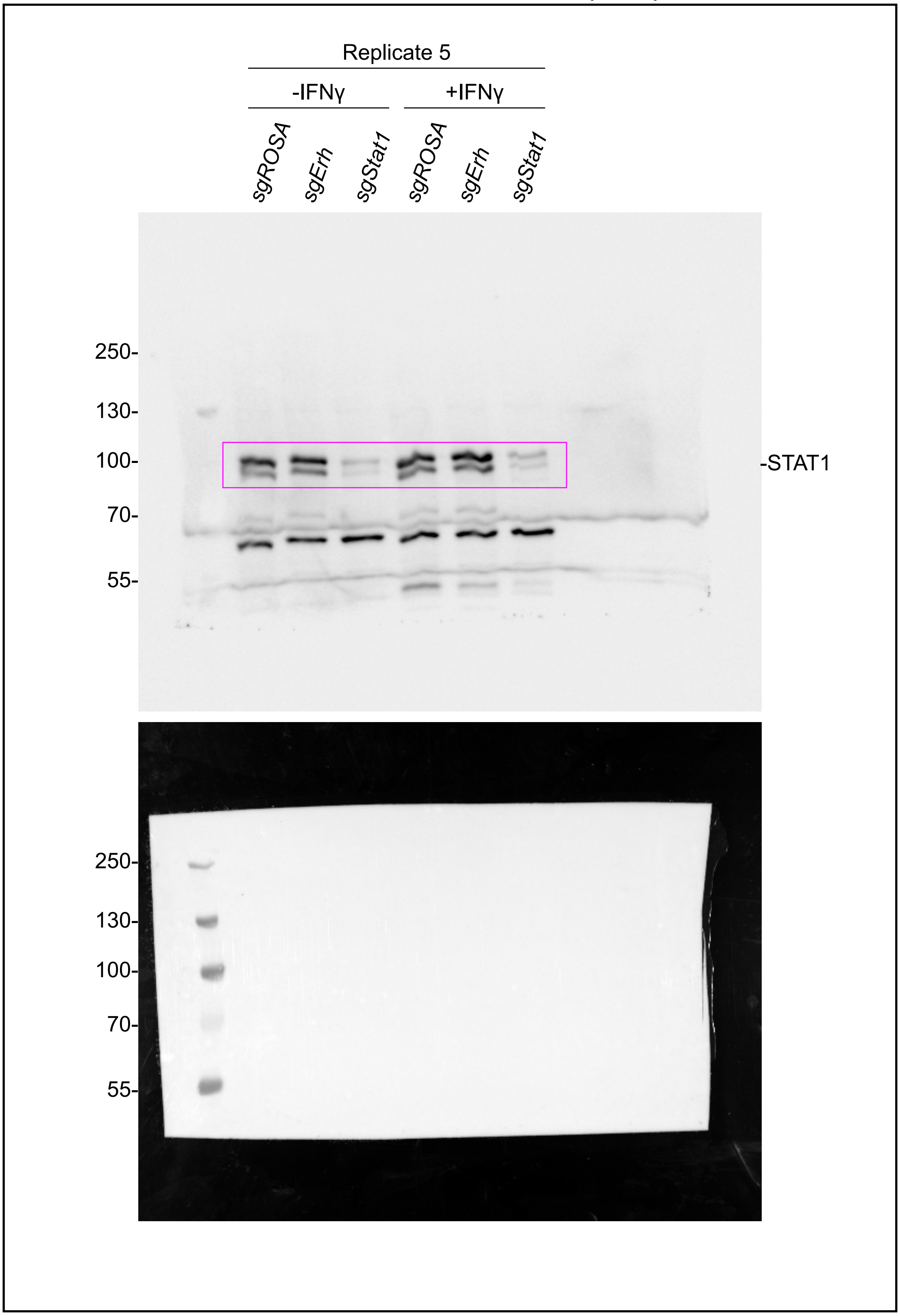

Souce_data_Fig_S2E

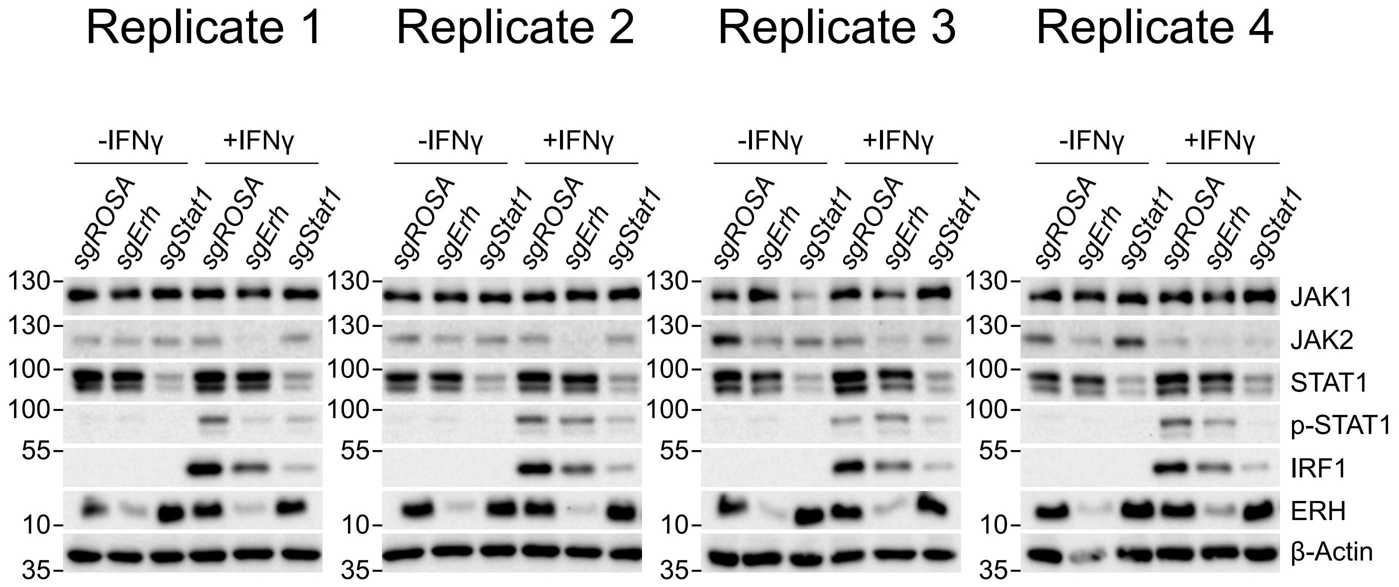

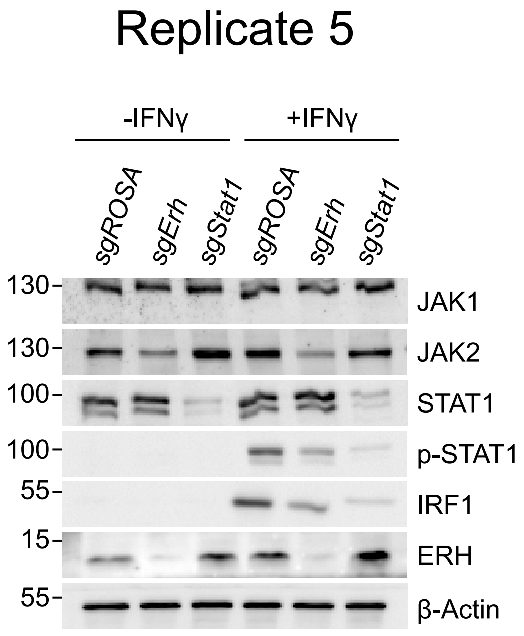

Souce_data_Fig_S3D

RKO, ERH (15% Gel)

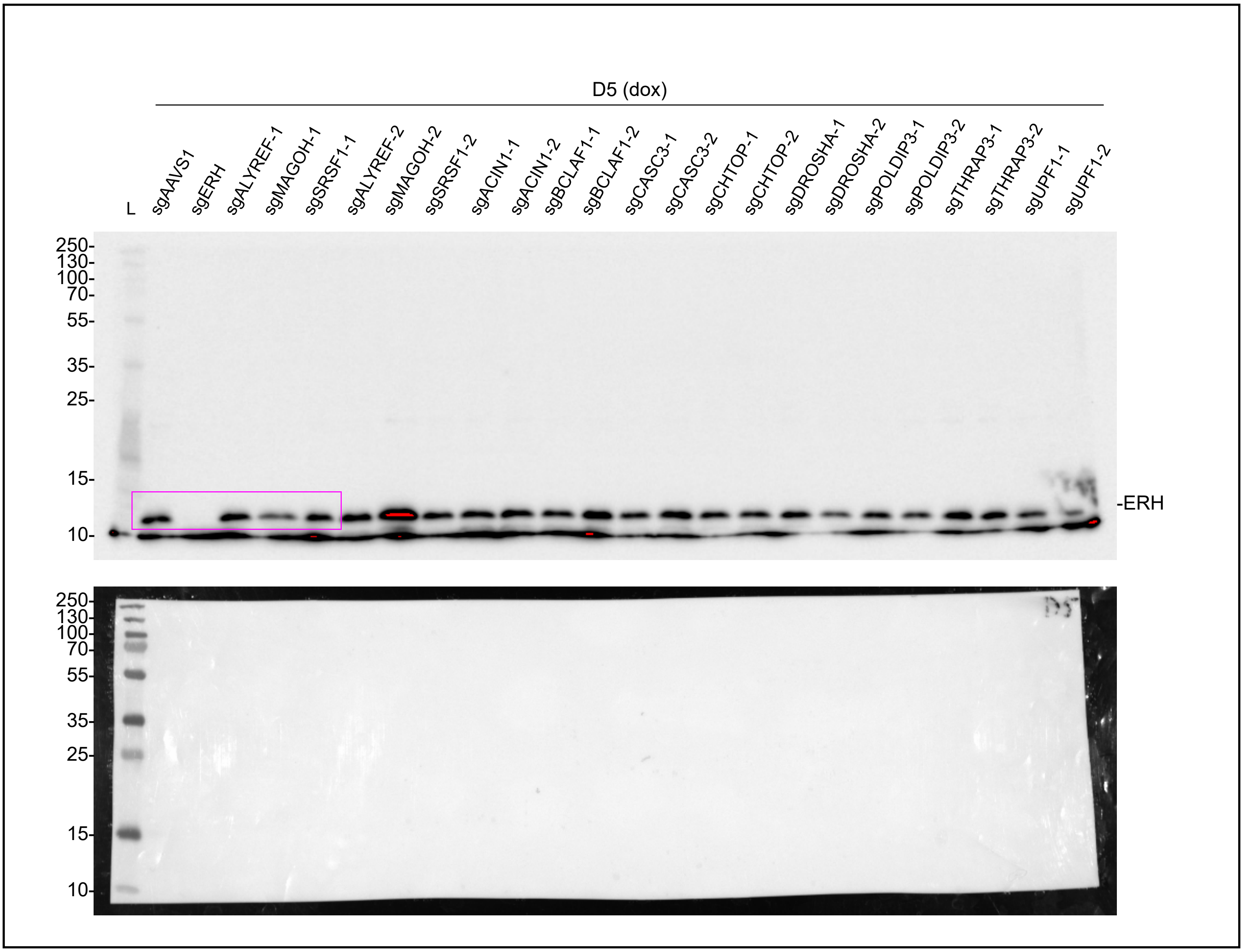

RKO, MAGOH (15% Gel)

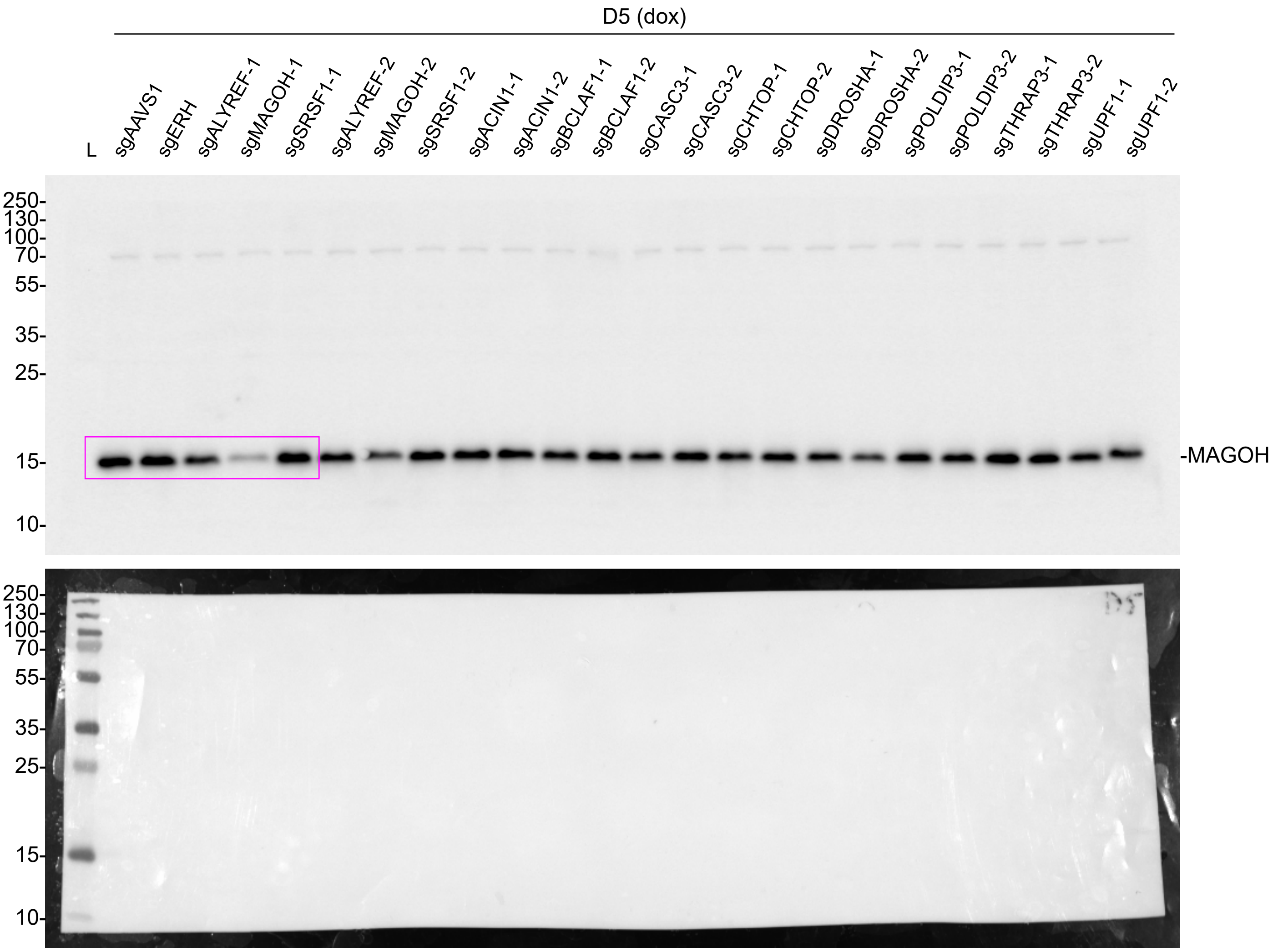

RKO, ALYREF (15% Gel)

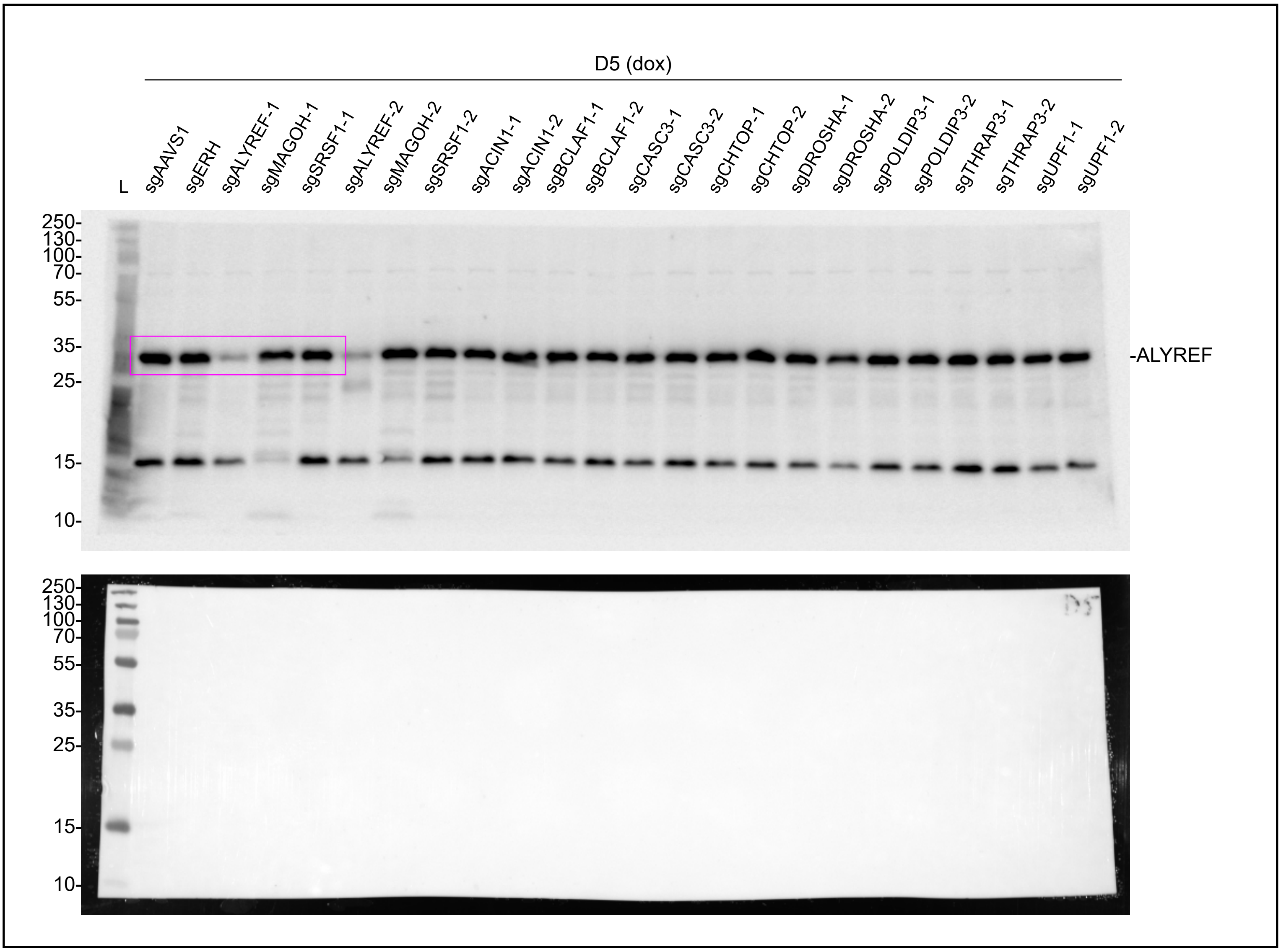

RKO, SRSF1 (15% Gel)

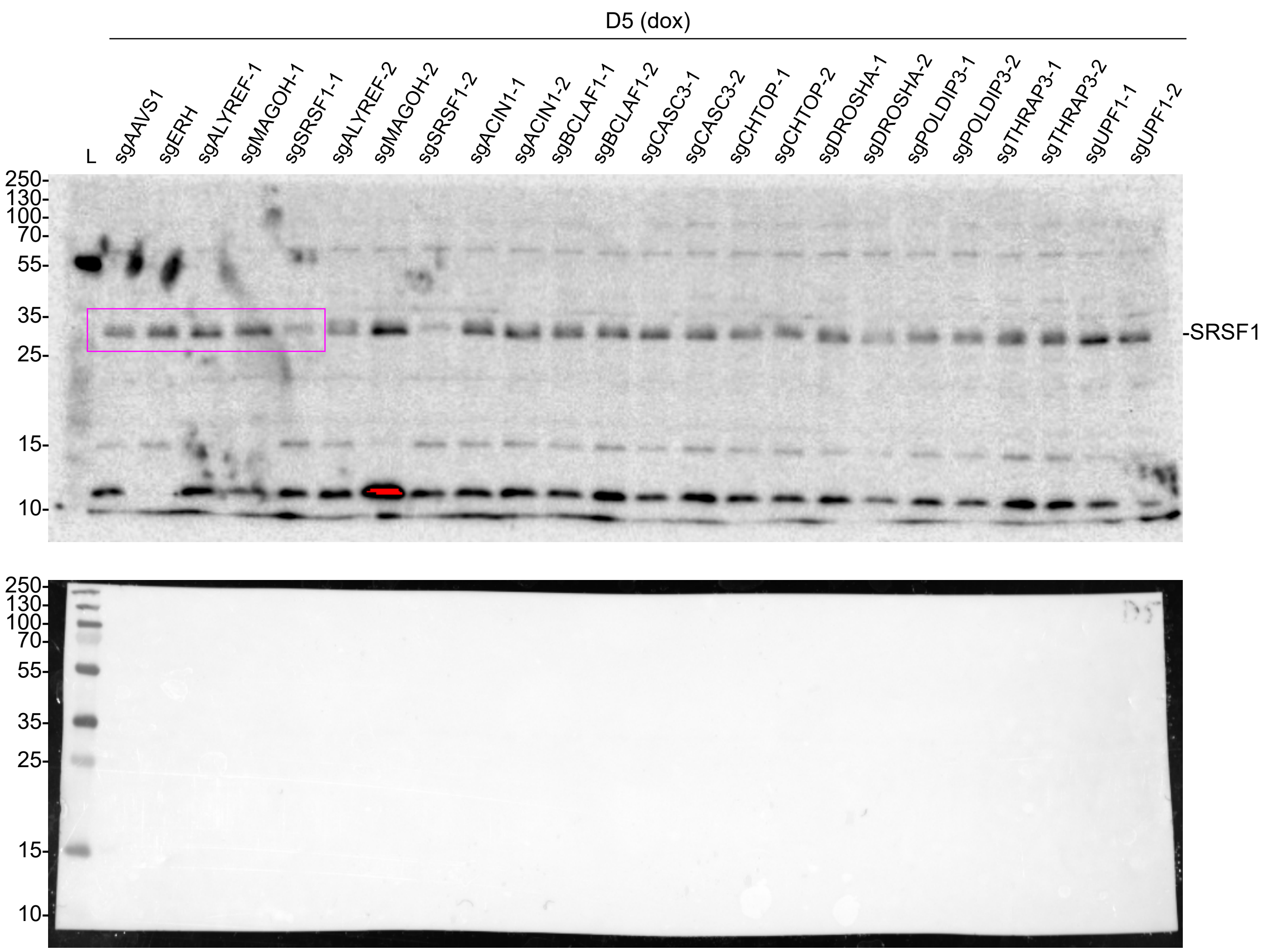

Souce_data_Fig_S3D

RKO, Vinculin (15% Gel)

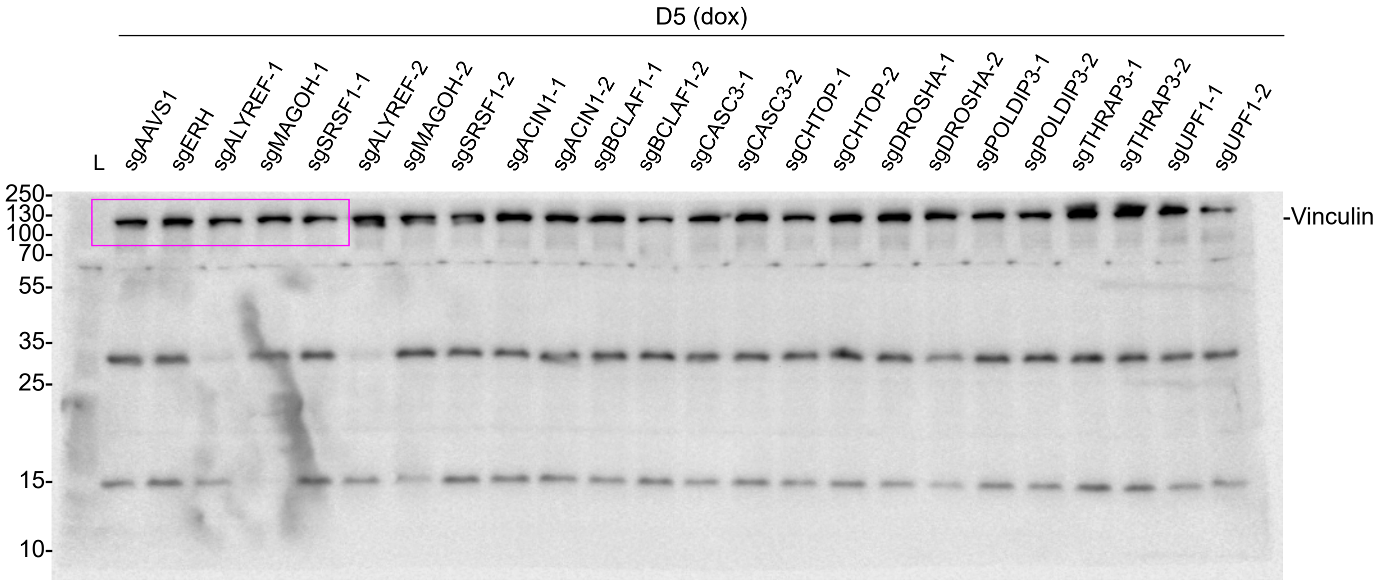

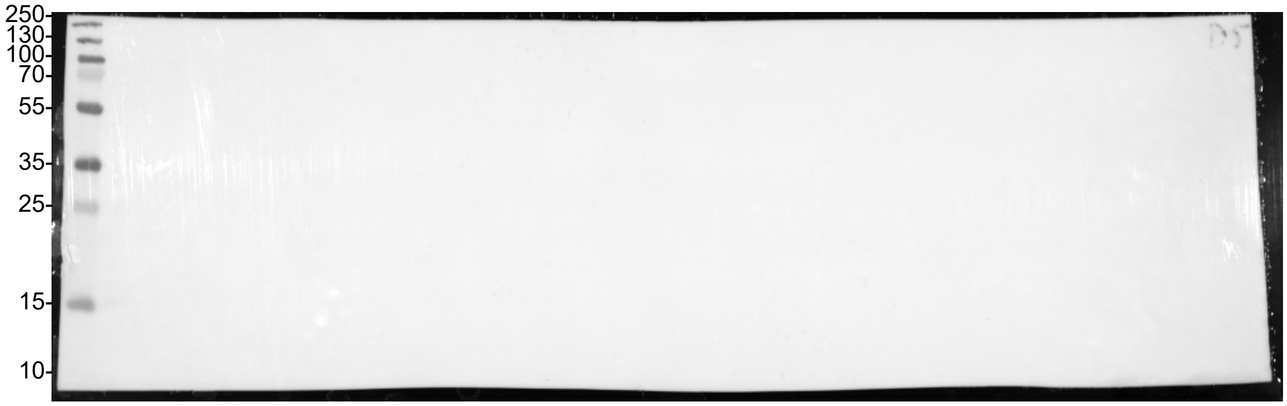

RKO, LAMIN A/C (10% Gel)

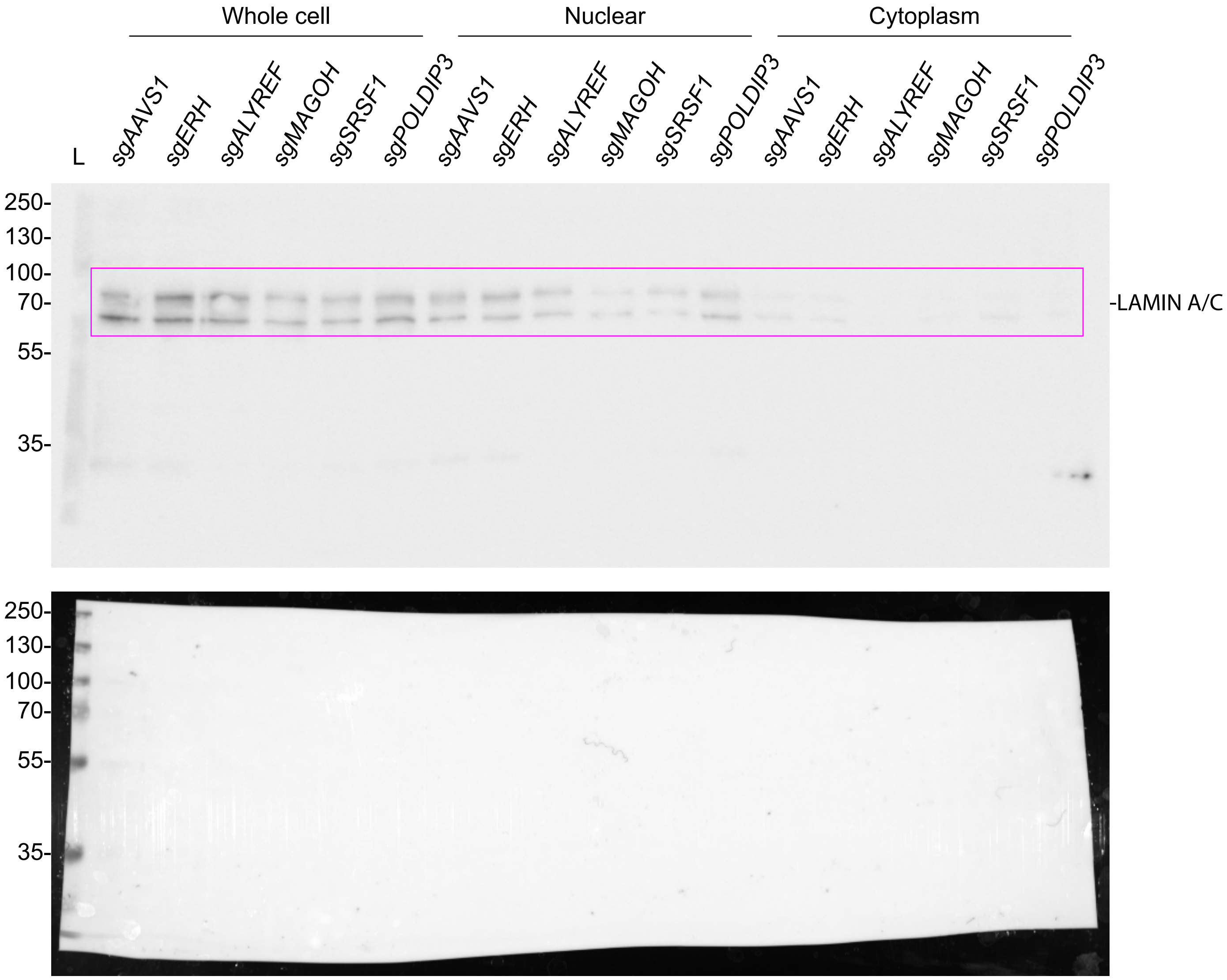

RKO, Vinculin (10% Gel)

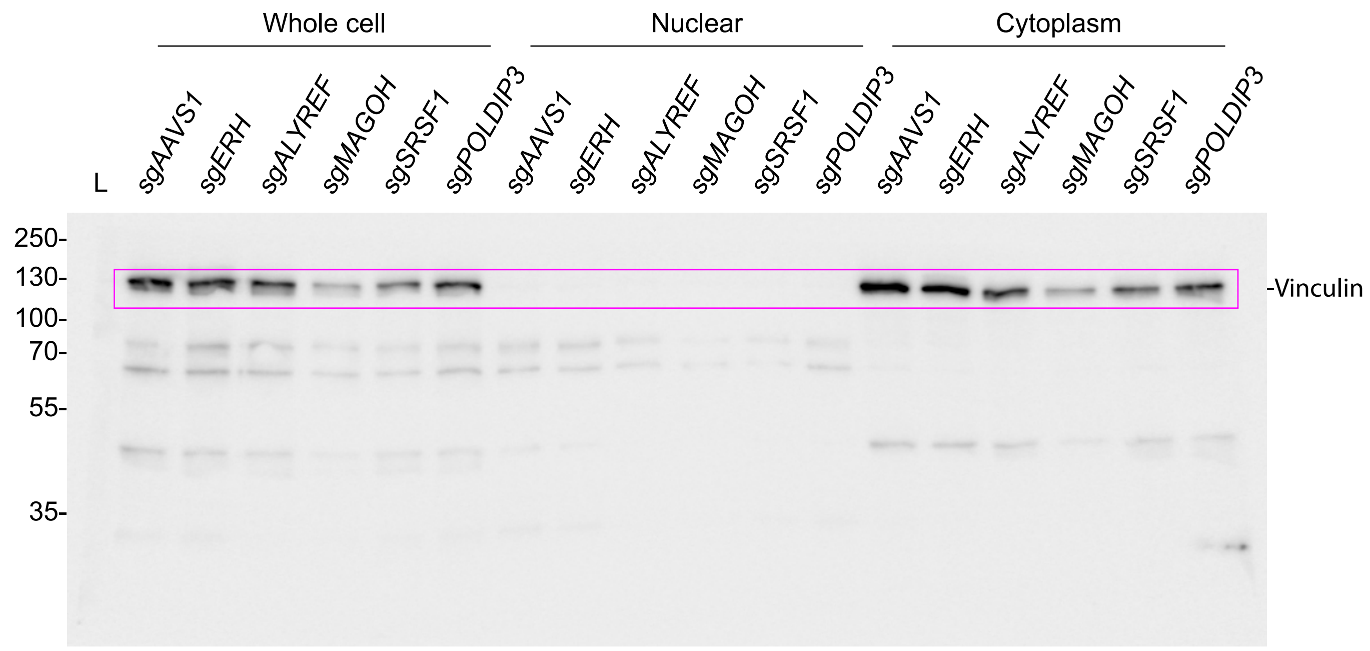

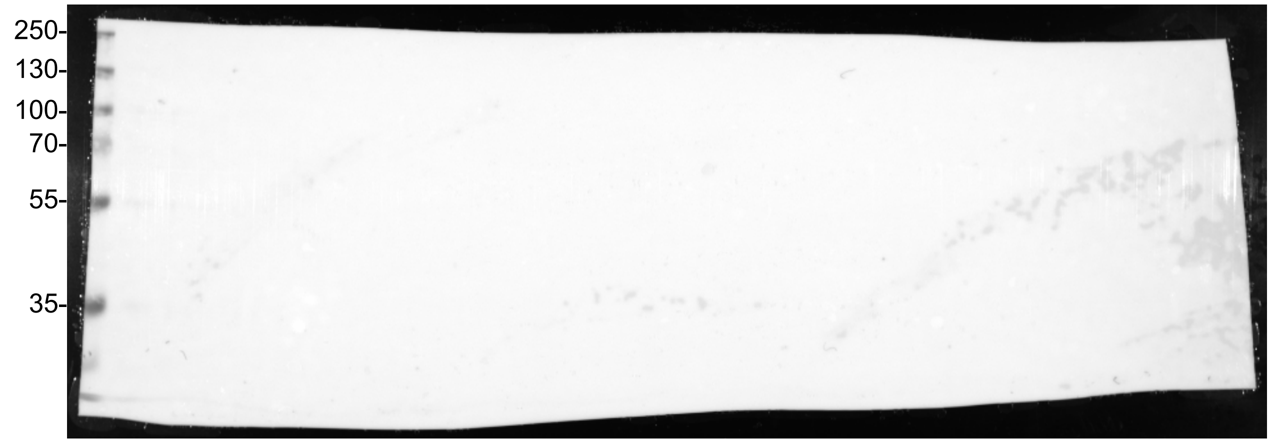

Supplement: gkaf545_Supplemental_Files [file gkaf545_supplemental_files.zip › Source_data.pdf]
