# Supplementary material for: ERH regulates type II interferon immune signaling through post-transcriptional regulation of JAK2 mRNA
Source: Nucleic Acids Res. 2025 Jun 30;53(12):gkaf545. doi: 10.1093/nar/gkaf545 (PMC12207402; doi:10.1093/nar/gkaf545)
Supplement: gkaf545_Supplemental_Files [file gkaf545_supplemental_files.zip › Supplementary_Figures.pdf]

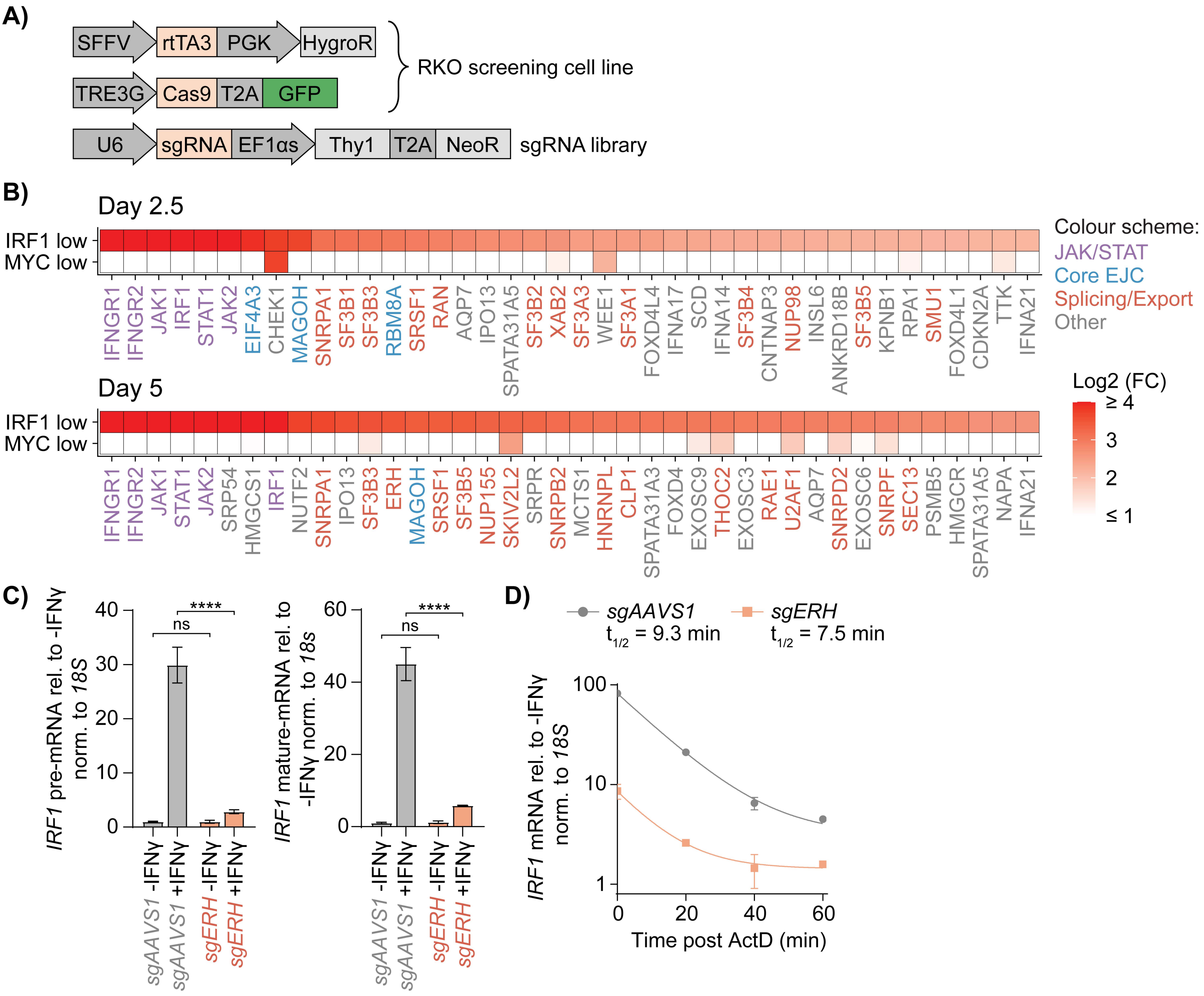
A)
SFFV
rtTA3
PGK
HygroR
TRE3G
Cas9
T2A
GFP
RKO screening cell line
U6
sgRNA
EF1αs
Thy1
T2A
NeoR
sgRNA library
B)
Day 2.5
IRF1 low
MYC low
IFNGR1 IFNGR2 JAK1 IRF1 STAT1 JAK2 EIF4A3 CHEK1 MAGOH SNRPA1 SF3B1 SF3B3 RBM8A SRSF1 RAN AQP7 IPO13 SPATA31A5 SF3B2 XAB2 SF3A3 WEE1 SF3A1 FOXD4L4 IFNA17 SCD IFNA14 SF3B4 CNTNAP3 NUP98 INSL6 ANKRD18B SF3B5 KPNB1 RPA1 SMU1 FOXD4L1 CDKN2A TTK IFNA21
Colour scheme:
JAK/STAT
Core EJC
Splicing/Export
Other
Day 5
IRF1 low
MYC low
IFNGR1 IFNGR2 JAK1 STAT1 JAK2 SRP54 HMGCS1 IRF1 NUTF2 SNRPA1 IPO13 SF3B3 ERH MAGOH SRSF1 SF3B5 NUP155 SKIV2L2 SRPR SNRPB2 MCTS1 HNRNPL CLP1 SPATA31A3 FOXD4 EXOSC9 THOC2 EXOSC3 RAE1 U2AF1 AQP7 SNRPD2 EXOSC6 SNRPF SEC13 PSMB5 HMGCR SPATA31A5 NAPA IFNA21
Log2 (FC)
≥ 4
3
2
≤ 1
C)
IRF1 pre-mRNA rel. to -IFNγ norm. to 18S
40
30
20
10
0
ns
****
sgAAVS1 -IFNγ
sgAAVS1 +IFNγ
sgERH -IFNγ
sgERH +IFNγ
IRF1 mature-mRNA rel. to -IFNγ norm. to 18s
60
40
20
0
ns
****
sgAAVS1 -IFNγ
sgAAVS1 +IFNγ
sgERH -IFNγ
sgERH +IFNγ
D)
sgAAVS1
t1/2 = 9.3 min
sgERH
t1/2 = 7.5 min
IRF1 mRNA rel. to -IFNγ norm. to 18S
100
10
1
0
20
40
60
Time post ActD (min)

Figure S1. Identification of ERH and other novel positive regulators of IFNγ signaling by genome-wide genetic screening.

(**A**) Constructs expressed in the RKO-iCas9 cell line used for the genetic screen. (**B**) Heatmaps showing enrichment of the top 40 positive IRF1 regulators in RKO cells at 2.5 or 5 days of Cas9 induction from the IRF1 genetic screen (Fig. 1D) or a comparable genetic screen for MYC regulators (de Almeida *et al*, 2021). Regulatory factors are color-coded for involvement in: JAK/STAT signaling (purple), EJC (blue), splicing and export (orange), or other processes (gray). (**C**) RKO-iCas9 cells expressing the indicated sgRNAs were induced with dox for 5 days, treated for 4 h with IFNγ, after which pre- and mature *IRF1* mRNA levels were measured by RT-qPCR. Data represent means and sd; n = 3 biological replicates. Two-tailed t-test with Benjamini-Hochberg correction (*p ≤ 0.05; **p ≤ 0.01; ***p ≤ 0.001; ****p ≤ 0.0001). (**D**) RKO-iCas9 cells expressing the indicated sgRNAs were induced with dox for 5 days, stimulated for 4 h with IFNγ, after which actinomycin D was added for the indicated times, and *IRF1* mRNA levels and half-life analyzed by RT-qPCR. Data represent means and s.d.; n = 3 biological replicates.

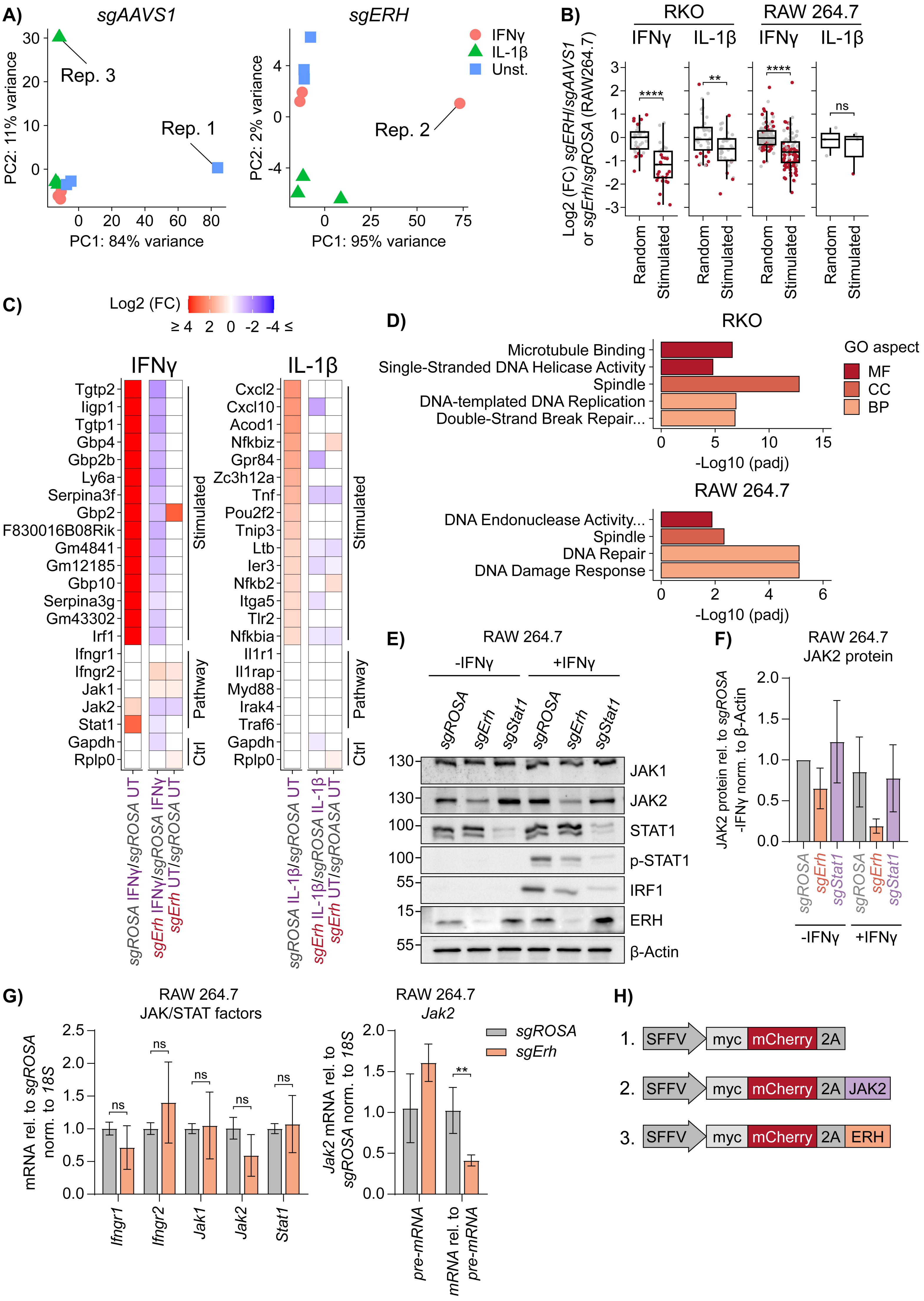
A)
sgAAVS1
PC2: 11% variance
PC1: 84% variance
Rep. 3
Rep. 1
sgERH
PC2: 2% variance
PC1: 95% variance
Rep. 2
IFNγ
IL-1β
Unst.
B)
RKO
RAW 264.7
IFNγ
IL-1β
Log2 (FC) sgERH/sgAAVS1 or sgErh/sgROSA (RAW264.7)
****
**
ns
Random
Stimulated
C)
Log2 (FC)
≥ 4 2 0 -2 -4 ≤
IFNγ
Tgtp2
Iigp1
Tgtp1
Gbp4
Gbp2b
Ly6a
Serpina3f
Gbp2
F830016B08Rik
Gm4841
Gm12185
Gbp10
Serpina3g
Gm43302
Irf1
Ifngr1
Ifngr2
Jak1
Jak2
Stat1
Gapdh
Rplp0
Stimulated
Pathway
Ctrl
sgROSA IFNγ/sgROSA UT
sgErh IFNγ/sgROSA IFNγ
sgErh UT/sgROSA UT
IL-1β
Cxcl2
Cxcl10
Acod1
Nfkbiz
Gpr84
Zc3h12a
Tnf
Pou2f2
Tnip3
Ltb
Ier3
Nfkb2
Itga5
Tlr2
Nfkbia
Il1r1
Il1rap
Myd88
Irak4
Traf6
Gapdh
Rplp0
sgROSA IL-1β/sgROSA UT
sgErh IL-1β/sgROSA IL-1β
sgErh UT/sgROASA UT
D)
RKO
Microtubule Binding
Single-Stranded DNA Helicase Activity
Spindle
DNA-templated DNA Replication
Double-Strand Break Repair...
-Log10 (padj)
GO aspect
MF
CC
BP
RAW 264.7
DNA Endonuclease Activity...
Spindle
DNA Repair
DNA Damage Response
-Log10 (padj)
E)
RAW 264.7
-IFNγ
+IFNγ
sgROSA
sgErh
sgStat1
JAK1
JAK2
STAT1
p-STAT1
IRF1
ERH
β-Actin
F)
RAW 264.7
JAK2 protein
JAK2 protein rel. to sgROSA -IFNγ norm. to β-Actin
sgROSA
sgErh
sgStat1
-IFNγ
+IFNγ
G)
RAW 264.7
JAK/STAT factors
mRNA rel. to sgROSA norm. to 18S
ns
Ifngr1
Ifngr2
Jak1
Jak2
Stat1
RAW 264.7
Jak2
Jak2 mRNA rel. to sgROSA norm. to 18S
**
pre-mRNA
mRNA rel. to pre-mRNA
sgROSA
sgErh
H)
1. SFFV myc mCherry 2A
2. SFFV myc mCherry 2A JAK2
3. SFFV myc mCherry 2A ERH

Figure S2. ERH is essential to maintain *JAK2* mRNA and protein levels, and consequently critical for IFNγ signaling.

(A) PCA analysis for the 3' QuantSeq analysis of RKO-iCas9 cells expressing the indicated sgRNAs and stimulated with the indicated treatments. Replicate outliers excluded from downstream analysis (Fig. 2A) are labeled. (**B**) Boxplots comparing the differential expression of stimulated genes in control cells (log2 fold change ≥ 2 and padj ≤ 0.05) or the same number of randomly selected genes in RKO-iCas9 or RAW 264.7-iCas9 cells expressing the indicated sgRNAs and stimulated with indicated cytokines. Statistically non-significant genes from differential expression analysis (padj > 0.05) are colored gray and significant genes (padj ≤ 0.05) are colored red. Two-sided Wilcoxon Rank Sum test (*p ≤0.05; **p ≤ 0.01; ***p ≤ 0.001; ****p ≤ 0.0001). (**C**) Cas9 was induced for 5 days in RAW 264.7-iCas9 cells expressing the indicated sgRNAs, stimulated for 4 h with IFNγ or IL-1β, after which RNA levels were quantified by 3' QuantSeq. The top 15 stimulated genes and pathway components are shown. Non-significant changes (padj ≤ 0.1) were given a 0-fold change difference. n = 3 biological replicates. (**D**) Selected top enriched GO terms for the subset of mRNAs with reduced expression (log2 fold change ≤ -1 and padj ≤ 0.05) from *ERH* ablation at non-stimulated conditions by 3' mRNA-Seq in RKO-iCas9 and RAW 264.7-iCas9 cells. MF, molecular function; CC, cellular component; BP, biological process. (**E**) Dox-induced RAW 264.7-iCas9 cells expressing the indicated sgRNAs were analyzed by WB, and (**F**) signal was quantified. Data represent means and sd; n = 5 biological replicates. (**G**) RAW 264.7-iCas9 cells with the indicated genes targeted for 5 days were analyzed by RT-qPCR. Data represent means and sd; n = 3 biological replicates. Two-tailed t-test with Benjamini-Hochberg correction (*p ≤ 0.05; **p ≤ 0.01; ***p ≤ 0.001; ****p ≤ 0.0001). (**H**) Constructs used for (1.) empty parental control vector, (2.) constitutive *JAK2* cDNA expression, or (3.) constitutive sgRNA resistant *ERH* cDNA expression in RKO-iCas9 cells.

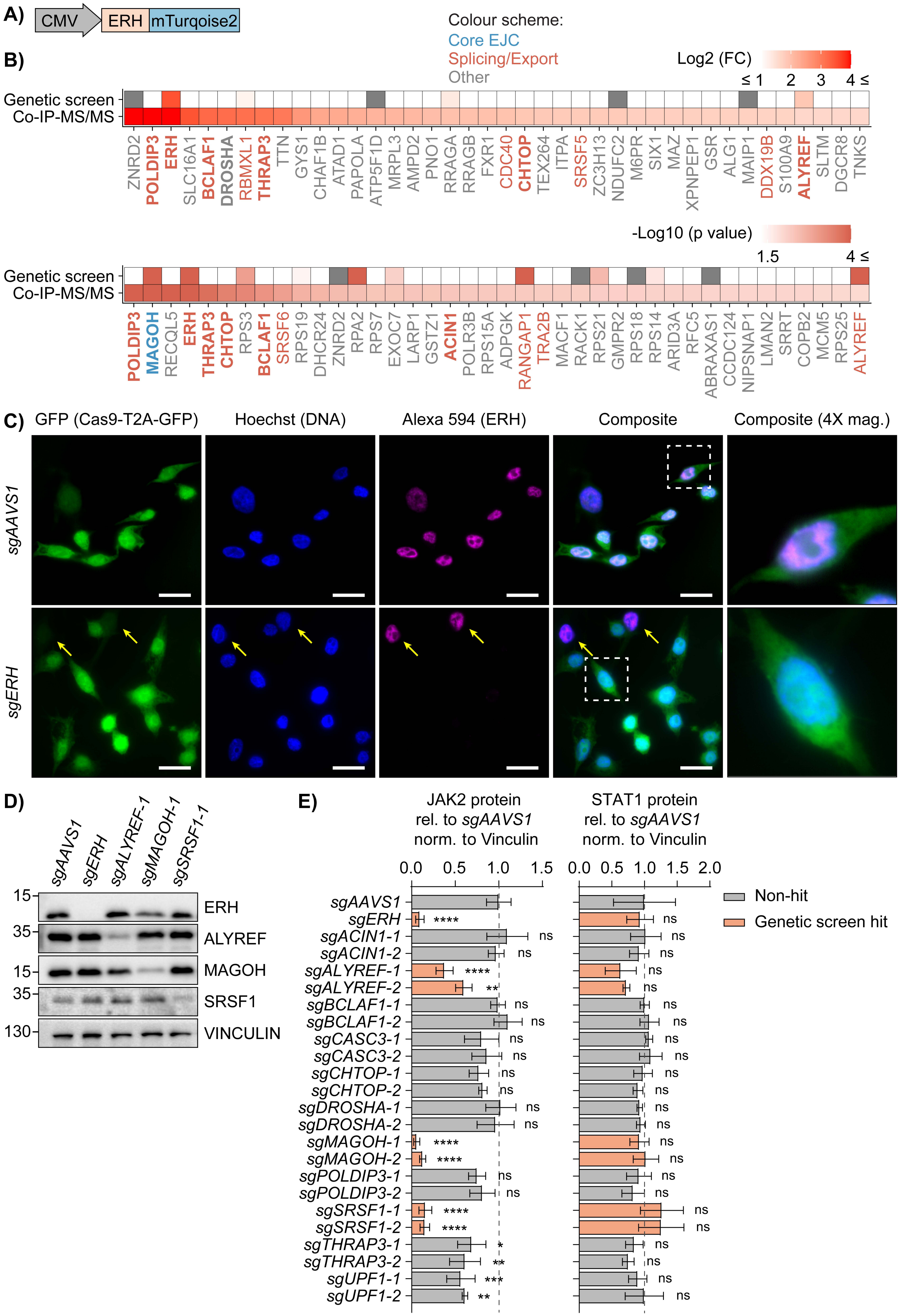

A)
CMV
ERH
mTurqoise2
B)
Colour scheme:
Core EJC
Splicing/Export
Other
Log2 (FC)
≤ 1
2
3
4 ≤
Genetic screen
Co-IP-MS/MS
ZNRD2 POLDIP3 ERH SLC16A1 BCLAF1 DROSHA RBMXL1 THRAP3 TTN GYS1 CHAF1B ATAD1 PAPOLA ATP5F1D MRPL3 AMPD2 PNO1 RRAGA RRAGB FXR1 CDC40 CHTOP TEX264 ITPA SRSF5 ZC3H13 NDUFC2 M6PR SIX1 MAZ XPNPEP1 GSR ALG1 MAIP1 DDX19B S100A9 ALYREF SLTM DGCR8 TNKS
-Log10 (p value)
1.5
4 ≤
Genetic screen
Co-IP-MS/MS
POLDIP3 MAGOH RECQL5 ERH THRAP3 CHTOP RPS3 BCLAF1 SRSF6 RPS19 DHCR24 ZNRD2 RPA2 RPS7 EXOC7 LARP1 GSTZ1 ACIN1 POLR3B RPS15A ADPGK RANGAP1 TRA2B MACF1 RACK1 RPS21 GMPR2 RPS18 RPS14 ARID3A RFC5 ABRAXAS1 CCDC124 NIPSNAP1 LMAN2 SRRT COPB2 MCM5 RPS25 ALYREF
C)
GFP (Cas9-T2A-GFP)
Hoechst (DNA)
Alexa 594 (ERH)
Composite
Composite (4X mag.)
sgAAVS1
sgERH
D)
sgAAVS1
sgERH
sgALYREF-1
sgMAGOH-1
sgSRSF1-1
15
35
15
35
130
ERH
ALYREF
MAGOH
SRSF1
VINCULIN
E)
JAK2 protein rel. to sgAAVS1 norm. to Vinculin
STAT1 protein rel. to sgAAVS1 norm. to Vinculin
0.0 0.5 1.0 1.5
0.0 0.5 1.0 1.5 2.0
Non-hit
Genetic screen hit
sgAAVS1
sgERH **** ns
sgACIN1-1 ns ns
sgACIN1-2 ns ns
sgALYREF-1 **** ns
sgALYREF-2 ** ns
sgBCLAF1-1 ns ns
sgBCLAF1-2 ns ns
sgCASC3-1 ns ns
sgCASC3-2 ns ns
sgCHTOP-1 ns ns
sgCHTOP-2 ns ns
sgDROSHA-1 ns ns
sgDROSHA-2 ns ns
sgMAGOH-1 **** ns
sgMAGOH-2 **** ns
sgPOLDIP3-1 ns ns
sgPOLDIP3-2 ns ns
sgSRSF1-1 **** ns
sgSRSF1-2 **** ns
sgTHRAP3-1 * ns
sgTHRAP3-2 ** ns
sgUPF1-1 *** ns
sgUPF1-2 ** ns

Figure S3. EJC-associated splicing and export factors interact with ERH and are critical for JAK2 production.

(**A**) Construct used for ERH co-immunoprecipitation (IP) and tandem mass spectrometry (MS/MS) in HEK-293T cells. (**B**) Heatmaps showing the top 40 enriched proteins in ERH co-IP-MS/MS based on fold change or adjusted p value, and corresponding enrichment in the genetic screen of IFNγ regulators (Fig. 1C). The timepoint (Cas9 induction) with lowest p value or highest fold change from the genetic screen is shown. Factors are color-coded for involvement in: EJC (blue), splicing and export (orange), or other processes (gray). (**C**) Representative immunofluorescent light microscopy images (scale bar, 50 µm) of RKO-iCas9 cells with indicated knockouts (left side) after 5 days of Cas9 induction. Co-expressed GFP was used to mark the cytoplasm and nucleus, whereas DNA (Hoechst) exclusively marked the nucleus. 4X magnification close-up views that visualize the sub-cellular localization of ERH are indicated with dashed white lines. Yellow arrows indicate non-knockout cells in *sgERH* samples. (**D**) Immunoblot from lysates of RKO-iCas9 cells with indicated knockouts after 5 days of Cas9 induction. (**E**) Quantified JAK2 and STAT1 protein levels from western blotting as represented in (Fig. 3E). Data represent means and sd; n = 4 biological replicates per sgRNA. One-way ANOVA with Bonferroni's multiple comparison correction (*$p \leq 0.05$; **$p \leq 0.01$; ***$p \leq 0.001$; ****$p \leq 0.0001$).

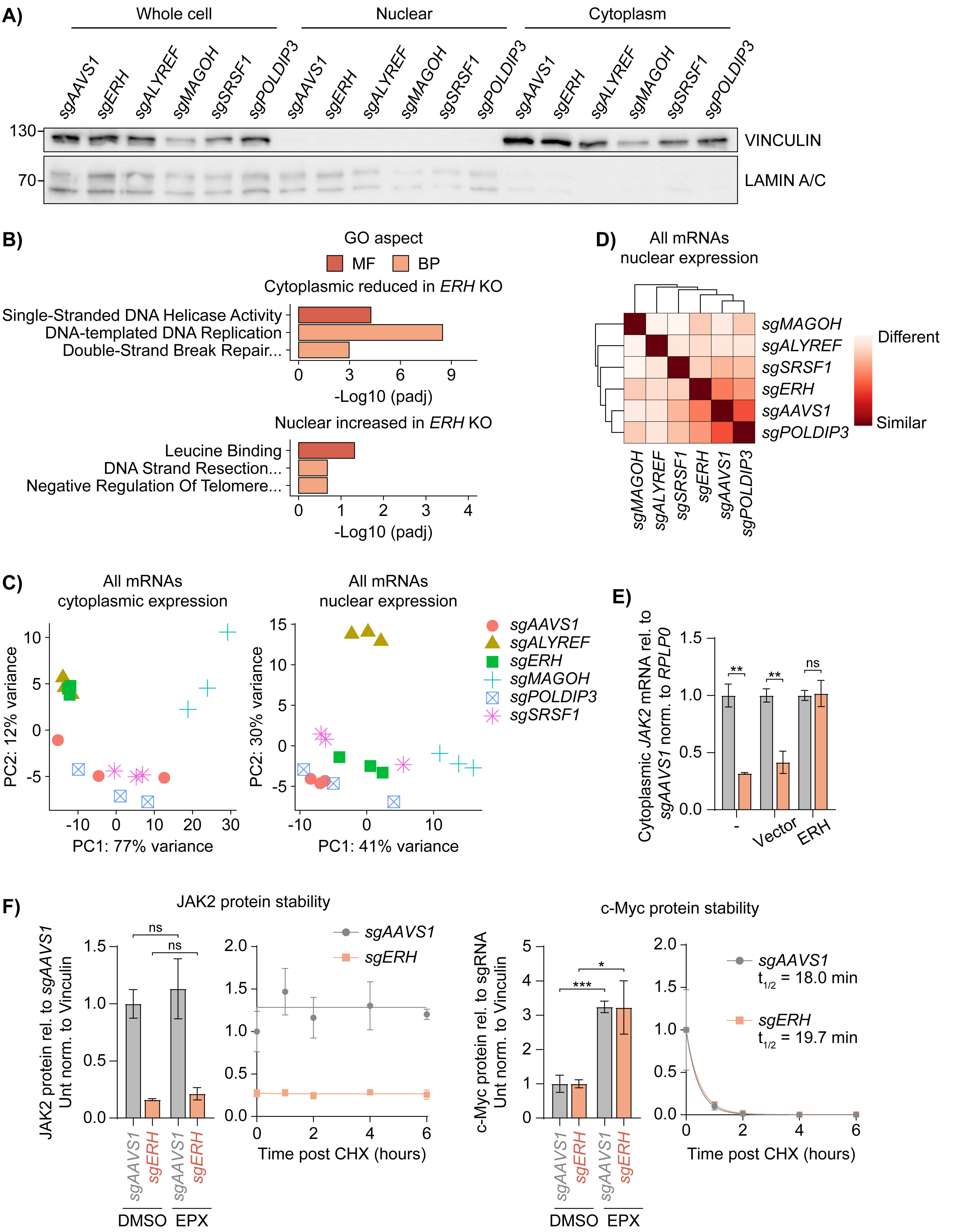

A)
Whole cell
Nuclear
Cytoplasm
sgAAVS1 sgERH sgALYREF sgMAGOH sgSRSF1 sgPOLDIP3
130
70
VINCULIN
LAMIN A/C
B)
GO aspect
MF BP
Cytoplasmic reduced in ERH KO
Single-Stranded DNA Helicase Activity
DNA-templated DNA Replication
Double-Strand Break Repair...
-Log10 (padj)
Nuclear increased in ERH KO
Leucine Binding
DNA Strand Resection...
Negative Regulation Of Telomere...
-Log10 (padj)
D)
All mRNAs
nuclear expression
sgMAGOH
sgALYREF
sgSRSF1
sgERH
sgAAVS1
sgPOLDIP3
Different
Similar
C)
All mRNAs
cytoplasmic expression
PC2: 12% variance
PC1: 77% variance
All mRNAs
nuclear expression
PC2: 30% variance
PC1: 41% variance
sgAAVS1
sgALYREF
sgERH
sgMAGOH
sgPOLDIP3
sgSRSF1
E)
Cytoplasmic JAK2 mRNA rel. to sgAAVS1 norm. to RPLP0
-
Vector
ERH
F)
JAK2 protein stability
JAK2 protein rel. to sgAAVS1 Unt norm. to Vinculin
DMSO
EPX
Time post CHX (hours)
c-Myc protein stability
c-Myc protein rel. to sgRNA Unt norm. to Vinculin
sgAAVS1 t1/2 = 18.0 min
sgERH t1/2 = 19.7 min
Time post CHX (hours)

Figure S4. ERH prevents faulty post-transcriptional *JAK2* mRNA processing.

(**A**) RKO-iCas9 cells with the indicated targeted genes used for 3' QuantSeq were fractionated and subsequently analyzed by WB. (**B**) Selected top-enriched GO terms for mRNA subsets (Fig. 4A). MF, molecular function; BP, biological process. (**C**) Principal component (PC) plots of cytoplasmic or nuclear mRNA expression from 3' QuantSeq samples. (**D**) Hierarchical clustered heatmap from 3' QuantSeq data assessing similarities between knockouts based on nuclear mRNA expression. **(E)** RKO-iCas9 cells with the indicated genes targeted for 5 days and transduced with an sgRNA-resistant *ERH* cDNA expressing vector or an empty vector (Fig. S2H) were subjected to subcellular fractionation, after which the RNA was isolated and analyzed by RT-qPCR. Data represent means and sd; n = 3 biological replicates. Unpaired t-test with Welch correction (*p ≤ 0.05; **p ≤ 0.01; ***p ≤ 0.001; ****p ≤ 0.0001). **(F)** Protein levels quantified from (Fig. 4D) to determine JAK2 and c-MYC protein stability. Data represent means and sd; n = 3 biological replicates. Unpaired t-test with Welch correction (*p ≤ 0.05; **p ≤ 0.01; ***p ≤ 0.001; ****p ≤ 0.0001).

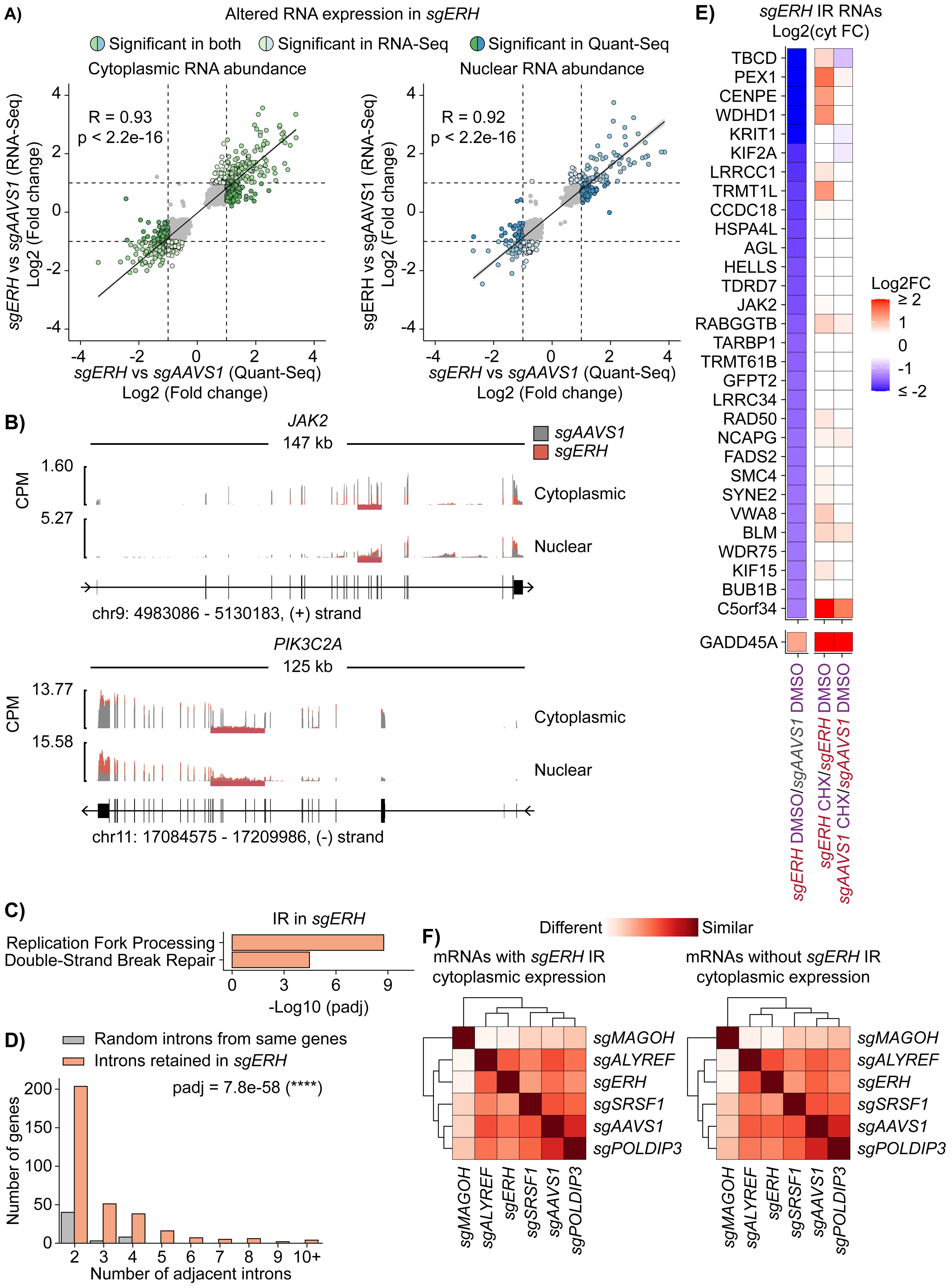
A)
Altered RNA expression in sgERH
Significant in both
Significant in RNA-Seq
Significant in Quant-Seq
Cytoplasmic RNA abundance
R = 0.93
p < 2.2e-16
Nuclear RNA abundance
R = 0.92
p < 2.2e-16
sgERH vs sgAAVS1 (RNA-Seq) Log2 (Fold change)
sgERH vs sgAAVS1 (Quant-Seq) Log2 (Fold change)
4
2
0
-2
-4
B)
JAK2
147 kb
sgAAVS1
sgERH
CPM
1.60
5.27
Cytoplasmic
Nuclear
chr9: 4983086 - 5130183, (+) strand
PIK3C2A
125 kb
13.77
15.58
chr11: 17084575 - 17209986, (-) strand
C)
IR in sgERH
Replication Fork Processing
Double-Strand Break Repair
0
3
6
9
-Log10 (padj)
D)
Random introns from same genes
Introns retained in sgERH
padj = 7.8e-58 (****)
Number of genes
200
150
100
50
0
2
3
4
5
6
7
8
9
10+
Number of adjacent introns
E)
sgERH IR RNAs
Log2(cyt FC)
TBCD
PEX1
CENPE
WDHD1
KRIT1
KIF2A
LRRCC1
TRMT1L
CCDC18
HSPA4L
AGL
HELLS
TDRD7
JAK2
RABGGTB
TARBP1
TRMT61B
GFPT2
LRRC34
RAD50
NCAPG
FADS2
SMC4
SYNE2
VWA8
BLM
WDR75
KIF15
BUB1B
C5orf34
GADD45A
Log2FC
≥ 2
1
0
-1
≤ -2
sgERH DMSO/sgAAVS1 DMSO
sgERH CHX/sgERH DMSO
sgAAVS1 CHX/sgAAVS1 DMSO
F)
Different
Similar
mRNAs with sgERH IR cytoplasmic expression
mRNAs without sgERH IR cytoplasmic expression
sgMAGOH
sgALYREF
sgERH
sgSRSF1
sgAAVS1
sgPOLDIP3

Figure S5. ERH prevents intron retention and disrupted export of *JAK2* and other select mRNAs.

RKO-iCas9 cells with the indicated knockouts were fractionated and subjected to poly(A)-enriched RNA-Seq analysis. (**A**) Comparison of altered cytoplasmic (left) or nuclear (right) RNA abundance in RKO-iCas9 cells expressing *sgERH* measured by QuantSeq or poly(A)-enriched RNA-Seq. Only mRNAs with reliably measured differences (padj ≤ 0.01) are shown. Genes with substantial expression differences are highlighted and indicated by dashed lines (absolute log2 fold change ≥ 1). Linear models with sd, and Pearson correlation coefficients and significances are reported. (**B**) *JAK2* and *PIK3C2A* full genes; mean reads per million (CPM) in *sgAAVS1* or *sgERH* are plotted. (**C**) Selected top enriched biological process GO terms for mRNAs with intron retention (IR) in *sgERH* over *sgAAVS1* in RKO-iCas9 cells. (**D**) Correlation analysis of the number of genes containing retained introns that are separated by one exon in *sgERH* samples, compared to randomly sampled introns in the same genes. Two-sided Wilcoxon Rank Sum test (****p ≤ 0.0001). (**E**) Heatmap of subcellularly fractionated poly(A)-enriched RNA-Seq, showing cytoplasmic expression differences for comparisons of RKO-iCas9 cells with indicated knockouts and treatments. The top 30 genes with reduced cytoplasmic expression in *sgERH* that have at least one increased ERH-regulated IR event are shown. (**F**) Hierarchical clustered heatmap depicting the distance between indicated knockout groups based on cytoplasmic expression.

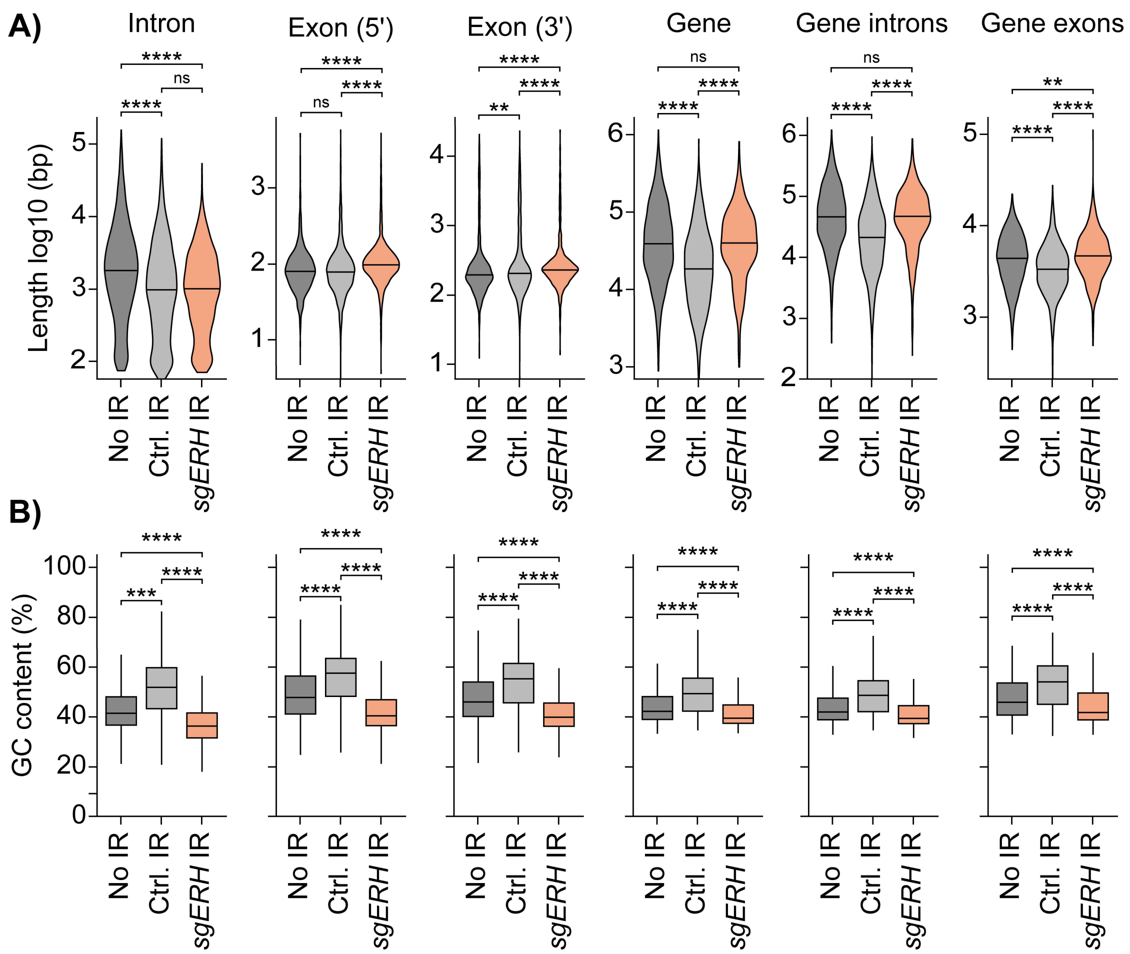
A)
Intron
Exon (5')
Exon (3')
Gene
Gene introns
Gene exons
Length log10 (bp)
No IR
Ctrl. IR
sgERH IR
ns
****
***
**
B)
GC content (%)
0
20
40
60
80
100

Figure S6. ERH-regulated retained introns and flanking exons are AU-rich.
RKO-iCas9 cells with the indicated knockouts were fractionated and subjected to poly(A)-enriched RNA-Seq analysis. (**A**) Violin plots comparing length distributions, and (**B**) box plots comparing GC content, between non-retained (No IR), normally retained (Ctrl. IR), and ERH-regulated retained introns (*sgERH* IR). Genomic regions compared from left to right: introns (Fig. 6A), flanking 5′ exon, flanking 3′ exon, the full gene, all introns in the gene, and all exons in the gene. Two-sided Wilcoxon Rank Sum test with Holm-Bonferroni correction (*p ≤0.05; **p ≤ 0.01; ***p ≤ 0.001; ****p ≤ 0.0001).
